# A metasurface-enabled green-smart window for intelligent wireless communications with high visible transparency and low infrared emissivity

**DOI:** 10.1038/s41467-026-72643-x

**Published:** 2026-05-13

**Authors:** Rui Zhe Jiang, Chuan Kui Shen, Hui Dong Li, Qun Yan Zhou, Zhi Hui Fu, Zheng Xing Wang, Terry Tao Ye, Jun Yan Dai, Lie Kun Yang, Jitong Ma, Qiang Cheng, Tie Jun Cui

**Affiliations:** 1 State Key Laboratory of Millimeter Waves, Southeast University, Nanjing, China; 2 Zhangjiang Laboratory, Shanghai, China; 3 Southern University of Science and Technology Jiaxing Research Institute, Jiaxing, China; 4 State Key Laboratory of Terahertz and Millimeter Waves, City University of Hong Kong, Hong Kong, China; 5 School of Science and Engineering, The Chinese University of Hong Kong, Shenzhen, China; 6 School of Information and Science, Dalian Maritime University, Dalian, China; 7 Suzhou Laboratory, Suzhou, China

**Keywords:** Engineering, Optical materials and structures

## Abstract

New-generation windows require advanced multi-physics modulation capability to accommodate diverse functions, including optical transparency, thermal insulation, and wireless communication compatibility. In response to these demands, metasurfaces have emerged as a promising solution owing to their versatile and energy-efficient electromagnetic (EM) wave manipulations. However, the existing prototypes suffer from fixed functionalities and limited intelligence, restricting their practicality and sustainability. Here, we show a green-smart window based on programmable metasurface to simultaneously achieve high visible transparency, low infrared (IR) emissivity, and reconfigurable EM responses. By integrating a compact RFID (radio frequency identification) tag, three intelligent functions are remotely reconfigured in a self-powering manner: EM transmission enhancement, selective EM shielding, and polarization filtering. For verification, a prototype is fabricated to validate its visible-IR-EM multi-physics controls, remote-sensing range, self-powering capability, and thermal management. Indoor-to-indoor (I2I) and outdoor-to-indoor (O2I) communication scenarios are established, demonstrating significant improvement in communication quality by using the green-smart window.

## Abstract (web summary)

The authors present exciting metasurface-enabled green-smart window that combines high visible transparency, low IR emissivity for temperature stabilization, and programmable electromagnetic functions from 0° to 80° under both transverse electric and transverse magnetic polarizations to improve the communication quality.

## Introduction

Windows are ubiquitous elements in daily life, serving as essential components in both building facades and transportation vehicles. They act as critical interfaces between indoor and outdoor environments, facilitating the exchange of various information and energies, which significantly influence human well-being and indoor environmental quality. In the context of emerging smart cities, advanced windows are increasingly expected to provide a range of valuable services that promote energy efficiency, environmental adaptability, and enhanced user comfort. In response to these requirements, remarkable progress has been made in photovoltaic windows and smart windows, which enable on-demand optical spectra modulation and solar radiation harvest and concurrently achieve daylighting, privacy protection, and energy conservation^1–8^. Despite these advancements, the majority of current advanced windows exhibit poor compatibility with the existing 4 G and 5 G wireless communication systems since their non-ideal radio-frequency (RF) properties deteriorate the quality of RF signals. Specifically, electrode-based advanced windows tend to entirely block the RF signal propagation, severely impairing the stability of wireless links established through the window^1–4^. Although dielectric-based advanced windows can offer an alternative solution, their limited capability to shield indoor-to-indoor (I2I) communications from outdoor RF interference results in degraded data transmission quality and high security risks^5,6,9–11^. In addition, their severely deteriorated RF transmission amplitudes at large incident angles lead to blind spots for indoor coverage^12–14^.

To improve the RF properties of windows, metasurface emerges as a highly promising solution due to its unprecedented capability to manipulate electromagnetic (EM) waves through planar structures^13–17^. Beyond the traditional passive metasurfaces with limited static functions, programmable metasurfaces enable dynamic engineering of the EM wavefront and adaptive optimization of the EM environment, making them a transformative platform for next-generation wireless networks^18–24^. As a special class among these works, visibly transparent metasurfaces have been highlighted due to their excellent advantages of easy deployment, little visual blocking, and multi-scenario applicability. With such merits, the visibly transparent metasurfaces become a prospective window technology to optimize the urban EM environments, especially in millimeter and terahertz bands^13,25–29^.

However, the design of metasurface-enabled windows still faces many challenges, such as trade-offs between optical and electrical properties, immaturity of manufacturing processes for transparent materials, and difficulty in metalizing vias on the transparent substrate. To address the above issues, some visibly transparent metasurfaces have been successively presented^30–32^, and some efforts have been taken to modify the multispectral characteristics of the metasurfaces^25,33^. Nevertheless, most reported works have still been confined to passive mode with static functions, and thus are incapable of adapting to complex environments actively. Limited studies are dedicated to creating tunable metasurfaces with visible transparency. For example, the optically controlled solution can eliminate the need for bulky power supplies and control circuits, but it is costly and prone to external light interference^34^; mechanically tunable technologies have been explored, but the slow switching speed and bulky configuration pose significant challenges in real applications^35^. Electrically tunable technologies represent a promising direction, and several studies showed their feasibility and encouraged further exploration^36–39^.

Although electrically tunable technologies are widely used in programmable metasurfaces, the demand for complicated control circuits and power supply impedes the deployment flexibility and operational efficiency, reducing both intelligence and sustainability of the system. Some studies have undertaken efforts to remove the cable networks by implementing optoelectronic modules^40^, acoustic-electric conversion modules^41^, and touch-sensing modules^42^. However, their limited control ranges and poor compatibility with Internet of Things (IoT) infrastructure seriously restrict their further development. In addition, the inclusion of active components presents a critical challenge: high energy costs. This issue becomes particularly acute when the programmable metasurfaces are deployed at a large scale, contradicting the principles of *green* and *low-carbon* industries. To address the energy challenge, low-power and self-powered techniques have been explored for metasurfaces^29,43–47^. Furthermore, photovoltaic windows designed to autonomously convert sunlight into electricity have attracted significant attention^7,8^. However, the electrically programmable metasurfaces are difficult to apply to windows due to their blockage of visible light, and integrating so many advanced functionalities into a single design remains a big challenge. Hence, it is essential to develop a cable-free programmable-metasurface-enabled window for green and smart cities.

In this work, we propose a metasurface-enabled green and smart window, aiming at intelligently optimizing the EM environments and minimizing energy consumption, while achieving high visible transparency and low infrared (IR) emissivity. The proposed window realizes the following visible-IR-RF multispectral capabilities to improve the user-centric services in smart cities: (1) The high visible transparency ensures natural daylighting. (2) The low IR emissivity helps stabilize indoor temperature to reduce energy expenditure from the indoor equipment. (3) The programmable RF functionalities are highlighted, including RF transmission enhancement (RFTE), selective RF shielding (SRFS), and polarization filtering, which can be operated within a large angle range from 0° to 80° under both transverse electric (TE) and transverse magnetic (TM) polarizations. Crucially, the proposed system incorporates a compact and multifunctional RFID tag, enabling cable-free, self-powered operation, remote reconfiguration, and seamless integration with existing IoT infrastructures. As a proof-of-concept, a prototype is designed, fabricated, and measured to validate the targeted multispectral features. Excellent energy-conservation capacity contributed by IR thermal insulation and efficient self-powering is also experimentally verified. The importance of the green-smart window in wireless networks is corroborated by two sets of communication experiments. This work enriches the solution to design visible-IR-RF multifunctional devices, opening up an avenue for future wireless communications and global sustainability.

## Results

### Design and operation of green-smart window

**Fig. 1:**
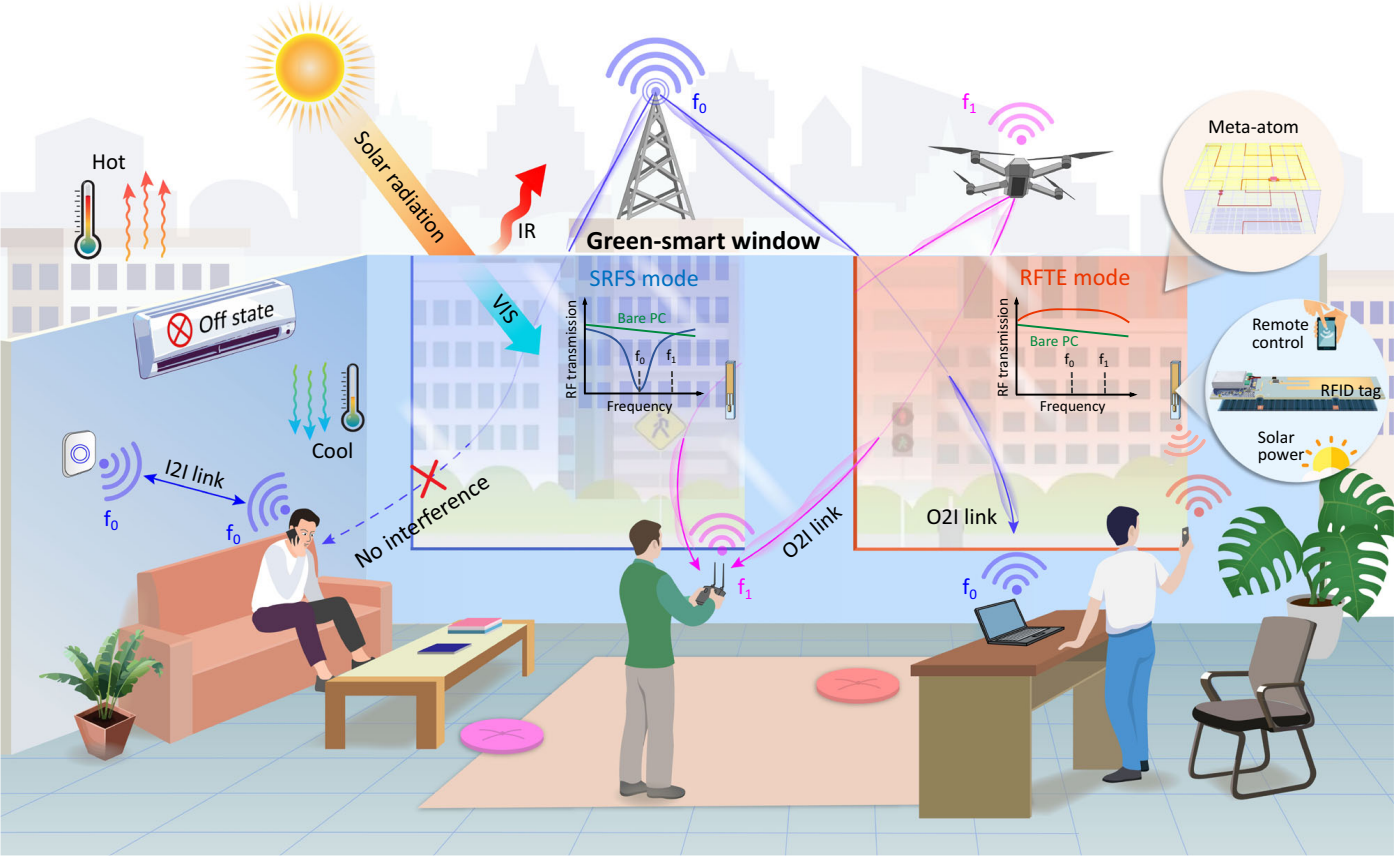
Conceptual diagram of the beneficial effects of the green-smart windows on indoor users. The windows can permit the visible light to transmit through to keep good daylighting performance; block most of the IR radiation to reduce the energy costs from the temperature-controlled systems by maintaining a suitable indoor temperature; and are reconfigured to the desired RF functional mode remotely to improve the quality of wide-angle wireless communications. The window on the right is switched to the RF transmission enhancement (RFTE) mode to enhance the O2I communication quality in a wide angular range and a wide frequency band (e.g., at *f*_0_ and *f*_1_ under oblique incidence). The window on the left is set to the selective RF shielding (SRFS) mode to protect the I2I links from the outdoor co-channel interference (e.g., at *f*_0_) without affecting the O2I links out of the band (e.g., at *f*_1_).

Figure 1 illustrates a representative application scenario of the green-smart window. To optimize the indoor wireless channel, the right-side window is remotely reconfigured as the RFTE mode to enhance wide-angle and wideband RF transmissions (e.g., at *f*_0_ and *f*_1_ under oblique incidence), thus reducing the indoor coverage blind spots and ensuring high-quality outdoor-to-indoor wireless links. The left-side window is set at the SRFS mode to prevent the indoor-to-indoor links at *f*_0_ from the outdoor co-channel interference, while enabling uninterrupted O2I links in other bands (e.g., at *f*_1_). In terms of energy conservation, the window holds the merits of the traditional low-emissivity (Low-E) window to suppress O2I thermal exchange, thereby saving the energy costs of indoor temperature-controlled devices. It can also reduce the energy consumption on the base station and user equipment sides by optimizing the wireless channel through self-sustaining solar-powered operation.

To meet the requirements of the application scenarios envisioned in Fig. 1, we target three specific design goals for multispectral manipulations: (1) reconfigure the RF responses between the RFTE and SRFS functional modes across a wide angular range under dual polarizations; (2) maintain high visible transparency; and (3) minimize the IR emissivity. For the green and smart operations, two additional goals are emphasized: (4) enable wireless and remote reconfiguration; and (5) ensure self-powering operation.

### Structure design for multispectral manipulation

**Fig. 2:**
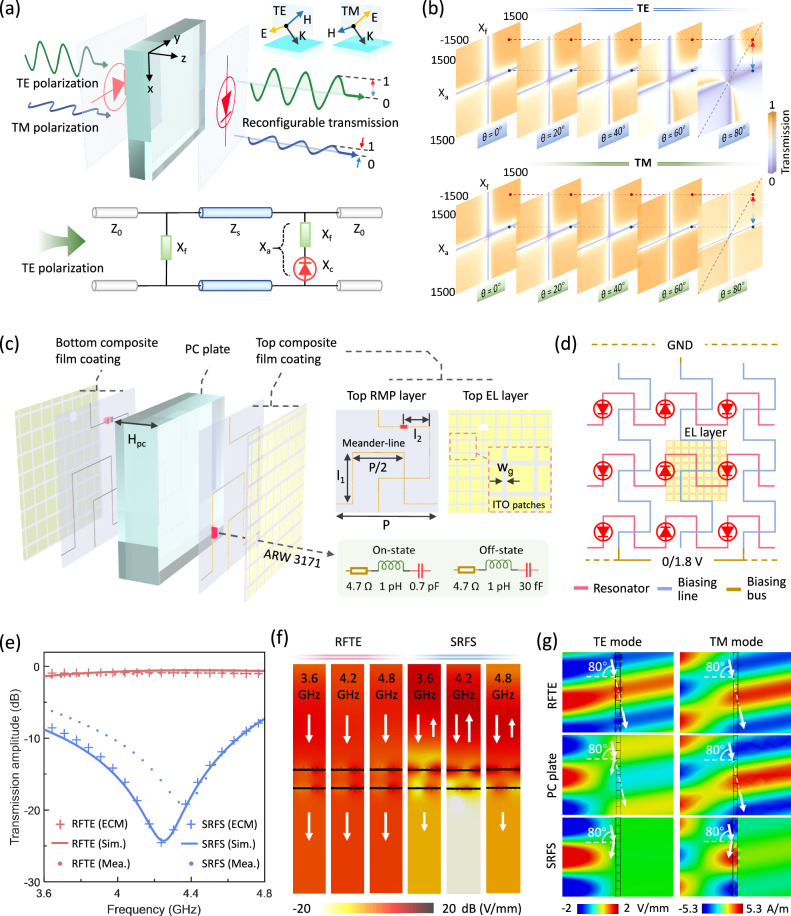
Design and performance evaluation of green-smart window metasurface. **a** The general coating-substrate-coating structure and its corresponding TL model. **b** Calculated transmission amplitudes for different *X*_*f*_ and *X*_*a*_ in the angle range from 0° to 80°. **c** The designed meta-atom and its geometric parameters. **d** The integration method between the resonators and the biasing lines. **e** The calculated, simulated, and measured transmission amplitudes of the meta-atom. **f** The time-averaged E-field intensity distribution in the RFTE and SRFS modes when the normal incident waves are at 3.6, 4.2, and 4.8 GHz. **g** The instant intensity distributions of the PC plate and meta-atom in the SRFS and RFTE modes when illuminated by 80° incidence at 4.2 GHz.

To achieve goals #1–3 for the multispectral manipulation, we propose a general coating-substrate-coating structure model to evaluate the design feasibility, as shown in Fig. 2a. For dynamically manipulating RF signals, the metasurface coatings loaded with active components are applied to both sides of the substrate in achieving independent controls of TE and TM polarizations. A transmission line (TL) model is established to quickly analyze the RF transmissions of the structure (see the subfigure on the bottom of Fig. 2a). The transmission matrix of the TL model is expressed as:

1 $$M=	 \left[\begin{array}{cc}A & B\\ C & D\end{array}\right]=\left[\begin{array}{cc}1 & 0\\ -i/{X}_{a} & 1\end{array}\right]\\ 	 \left[\begin{array}{cc}\cos ({k}_{s}{h}_{s}\cos {\theta }_{s}) & i{Z}_{s}^{{TE}/{TM}}\sin ({k}_{s}{h}_{s}\cos {\theta }_{s})\\ i\sin \left({k}_{s}{h}_{s}\cos {\theta }_{s}\right)/{Z}_{s}^{{TE}/{TM}} & \cos ({k}_{s}{h}_{s}\cos {\theta }_{s})\end{array}\right]\\ 	 \left[\begin{array}{cc}1 & 0\\ -i/{X}_{f} & 1\end{array}\right]$$

where *X*_*a*_ and *X*_*f*_ are surface reactances of the fixed and adjustable metasurface coatings, *h*_*s*_, *k*_*s*_, and *Z*_*s*_ denote the thickness, propagation constant, and intrinsic impedance of the substrate, and *θ*_*s*_ is the refraction angle in the substrate. By converting the transmission matrix to scattering matrix, the transmission coefficient *t* under TE and TM polarizations is calculated as

2 $${t}^{{TE}}=\,\frac{2}{A+B\cos {\theta }_{i}/{Z}_{0}+C{Z}_{0}/\cos {\theta }_{i}+D}$$

3 $${t}^{{TM}}=\,\frac{2}{A+B/{Z}_{0}/\cos {\theta }_{i}+C{Z}_{0}\cos {\theta }_{i}+D}$$

where *θ*_*i*_ is the incident angle. The calculated results in the angular range of 0°−80° under TE and TM polarizations are displayed in Fig. 2b. We prove that the first goal can be easily achieved by choosing appropriate values of *X*_*f*_ and *X*_*a*_ through optimization. To reach the second goal, visibly transparent dielectric materials and fine-line metallic structures can be employed in the coating. For the low IR emissivity required by the third goal, integrating the metasurface coatings with low-E materials is a viable solution. The feasibility of the proposed structure model in meeting the targeted design goals is validated in Supplementary Note 1.

Guided by the TL model, we design a dual-layer meta-atom as a fundamental building block of the green-smart window, as shown in Fig. 2c. The composite film coating is composed of a reconfigurable metal pattern (RMP) layer to enable reconfigurable RF responses and an etched low-E (EL) layer to provide thermal insulation. Two single-pole-single-throw RF switches with ultra-low power consumption are orthogonally loaded on the meta-atom to reconfigure the TE- and TM-polarized illumination independently. Considering the potential interference of biasing lines with RF performance, the biasing lines are designed in a meander-line type to seamlessly integrate with the resonators. Four biasing buses (two on top and two on bottom) are used to drive all RF switches on the metasurface-enabled window, as illustrated in Fig. 2d.

To illustrate the superior RF performance of the meta-atom, the transmission-amplitude spectra under normal incidence are depicted in Fig. 2e. We observe that the designed structure can realize nearly perfect transmission and nearly complete shielding. Remarkable frequency dispersion is also noticed, in which *t*_*RFTE*_ has a wide transmission band and *t*_*SRFS*_ has a narrow stop band. To explain this phenomenon, an equivalent circuit model (ECM) is established (see Supplementary Note 4), and the ECM-based results are in good agreement with the simulations. We remark that the transmission amplitudes can be continuously modulated in the range between *t*_*RFTE*_ and *t*_*SRFS*_, since the ARW3171 switch has an intermediate state. The calculation, simulation, and measurement results of the continuous amplitude modulations are detailed in Supplementary Note 5.

To visually illustrate the effects of RFTE and SRFS modes, the simulated time-average E-field intensity distributions at 3.6, 4.2, and 4.8 GHz are presented in Fig. 2f. For the RFTE mode, the EM waves of three frequencies can penetrate the structure without scattered waves. For the SRFS mode, the incident waves are nearly perfectly blocked at 4.2 GHz, while the waves at the out-of-band frequencies (3.6/4.8 GHz) can still transmit through the structure. The instant E- and H-field intensity distributions of the meta-atoms and a bare PC plate under the illumination at 80° are presented in Fig. 2g. When the meta-atom operates in the RFTE mode, the dominant traveling waves in full space indicate high transmission amplitudes under both TE and TM polarizations, illustrating significant transmission enhancement compared with the bare PC plate. In contrast, the spatial waves exhibit dominant standing-wave behavior in the SRFS mode due to the superposition of the incident and reflected waves.

**Fig. 3:**
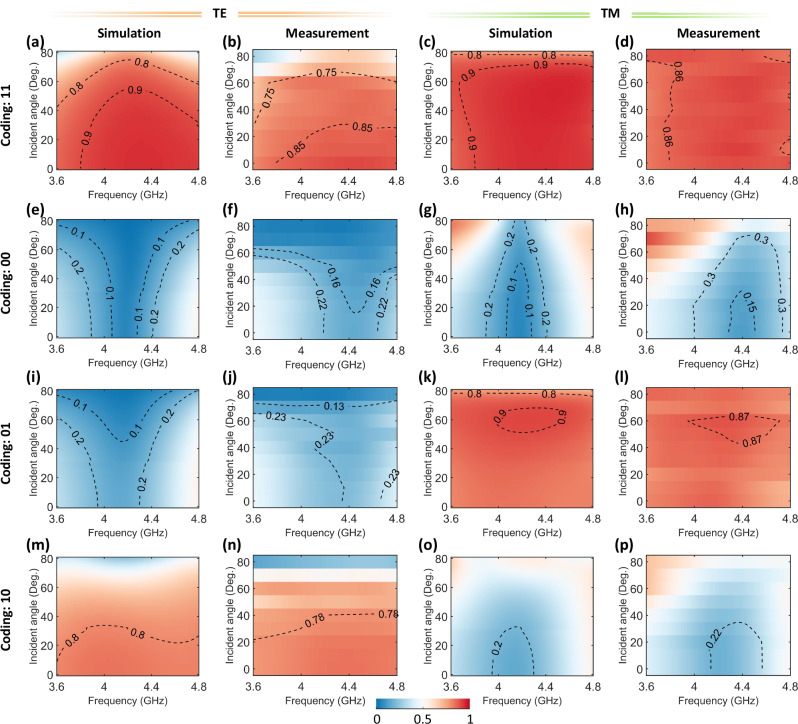
The simulated and measured transmission spectra of the green-smart window in ‘11’,’00’,’01’, and ‘11’ coding sequences. **a**, **e**, **i**, **m** The simulated results under the TE polarization from 0° to 80°. **b**, **f**, **j**, **n** The measured results under the TE polarization from 0° to 80°. **c**, **g**, **k**, **o** The simulated results under the TM polarization from 0° to 80°. **d**, **h**, **l**, **p** The measured results under the TM polarization from 0° to 80°.

To evaluate the angular stability of the green-smart window, the simulated transmission spectra in the angular range of 0°−80° under both TE and TM polarizations are illustrated in Fig. 3. When all RF switches are in the “on” state (coding sequence of ‘11’), the window exhibits high transmission amplitudes over a wide angular range, as depicted in Fig. 3a, c. In contrast, when all RF switches are in the “off” state (coding sequence of ‘00’), the window shows remarkable shielding performance, confirming the effectiveness of the SRFS mode, as illustrated in Fig. 3e, g. When the RF switches on the top and bottom are in the “off-on”/“on-off” states (coding sequences of ‘01’/‘10’), the window permits the TM/TE polarized incident waves to transmit through while blocks the TE/TM polarized incident waves, behaving as a polarization filter, as shown in Fig. 3i, k, m, and o. Therefore, through four coding sequences of ‘00’, ‘01’, ‘10’, and ‘11’, the window can modulate the transmissions of dual polarizations independently. These simulated results confirm that the proposed structure can successfully achieve the first design goal. In the following sections, we will present quantitative experimental validations of the wide-angle RF transmission, visible transmittance, and IR emissivity.

### Remote control and self-powering strategy

Large-scale deployment of smart windows faces significant challenges in operational practicality and sustainability. Conventional designs relying on complex wired connections restrict the control range and compromise architectural esthetics. Their continuous power demands increase the operational costs and contradict environmental sustainability principles. Inspired by the growing adoption of IoT technologies across various domains, the combination of smart windows with IoT systems offers a potential solution to address these problems. Among the IoT communication protocols, RFID technology emerges as a particularly promising one, offering distinct advantages including minimal power consumption, cost efficiency, and flexible deployment capabilities^48^.

**Fig. 4:**
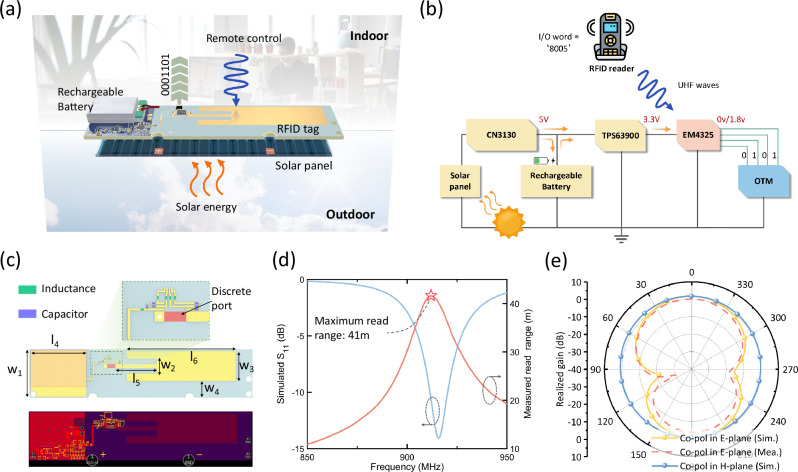
The RFID tag and its performance. **a** The application scenario of the RFID tag. **b** The system configuration for remote control and self-powering. **c** The structure of the RFID tag and its fabrication layout. **d** Simulated S_11_ parameters and measured read range. **e** Simulated and measured radiation patterns.

Here, we propose a strategy to simultaneously achieve remote control and self-powering for goals #4 and #5 through a compact RFID tag. The conceptual diagram and 3D model are shown in Fig. 4a. The tag operates by receiving control signals at UHF frequencies (915 MHz) from the indoor reader and outputting direct-current voltages at low or high levels, denoted as ‘0’ and ‘1’, respectively. A solar panel is integrated at the rear of the RFID tag to efficiently and consistently harness the natural energy for self-powering through the photoelectric conversion effect.

The detailed operational principle of the system is illustrated in Fig. 4b. Upon successful tag recognition, the I/O control word can be written in the user memory of the RFID tag integrated circuit (IC). The last 4 bits of the input/output (I/O) control word serve to configure the voltage level from the I/O ports to the biasing buses on the window. According to the RFID protocol, the functional modes of the green-smart window can be switched on the order of ten times per second, which is sufficient for many practical applications. The anode of the solar panel is connected to the input of the charging management IC to prolong the rechargeable battery’s life. The rechargeable battery can store the energy to ensure normal operation during weak or absent solar light conditions. In addition, the battery provides energy to the four I/O ports and enables the battery-assisted passive (BAP) mode of the RFID tag IC to extend its read range. An ultralow-static-power buck-boost converter with programmable output voltages is incorporated as a fore-stage circuit to regulate the supply voltage for the RFID tag IC. The system configurations are detailed in Supplementary Note 6.

To achieve high-gain and anti-metal properties, the RFID tag is designed in the form of a patch antenna. The geometric parameters of the tag are depicted in the upper panel of Fig. 4c. On the top layer, the digital circuits are densely arranged, and the digital ground plane is implemented as a large-area copper pour. Consequently, in full-wave simulations, the digital circuits are modeled as a complete metallic ground, and the battery is modeled as a metal block of equal size. A discrete port with an impedance *Z*_*EM4325*_** =** 7.6 – j114Ω representing the EM4325 IC is inserted between the patch and a short stub. The optimized geometric parameters are *l*_*4*_** =** 30 mm, *l*_*5*_** =** 22 mm, *l*_*6*_** =** 59 mm, *w*_*1*_** =** 26 mm *w*_*2*_** =** 8 mm, *w*_*3*_** =** 16 mm, and *w*_*4*_** =** 9 mm. As depicted in Fig. 4d, the −10 dB bandwidth of the simulated |S_11_| ranges from 910 to 920 MHz, indicating good impedance matching. To validate the effectiveness of the fabricated device, Voyantic^TM^ equipment is used to measure the RFID tag’s reading range in a semi-anechoic chamber. Measured results indicate that the maximum reading range is 41 m, which is sufficient to cover most of the indoor scenes. The simulated and measured gain patterns of the RFID tag exhibit excellent agreement, as shown in Fig. 4e. The experimental environment to measure the reading range and gain pattern is detailed in Supplementary Note 7.

Considering that the green-smart window may result in distorted radiation patterns of the RFID tag, we design and apply a small-area transparent absorber as an additional component to prevent the mutual coupling and improve the system stability (see Supplementary Note 8 for details). The simulated results show that the RFID tag performs well regardless of the size and functional modes of the window, thereby further improving the robustness of the proposed control and powering strategy.

### Visible performance

**Fig. 5:**
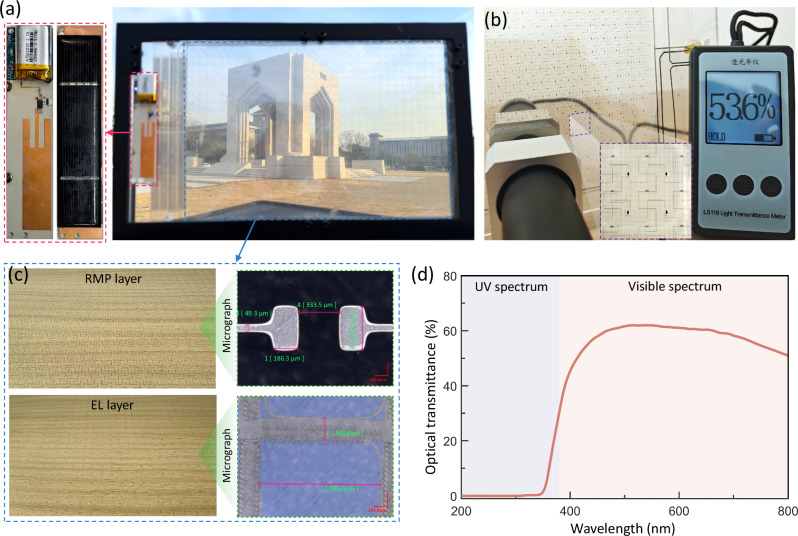
The fabricated green-smart window and its visible performance. **a** Prototype of the metasurface-enabled green-smart window. The front and back views of the RFID tag are shown in the inset. **b** The experimental setup to measure the averaged visible transmittance and the enlarged view of the prototype. **c** The photographs of fabricated RMP and EL layers (the left panel), and their microscope images (the right panel). **d** The measured optical transmittance spectra of the prototype in 200–800 nm.

Visible transmittance in the wavelength range from 380 to 780 nm is a key performance metric for evaluating the smart window functionality. In our design, the measured visible transmittance of the 5-mm-thick bare PC plate is up to 90.2% (see Supplementary Note 9 for details). To minimize the visual blockage, ultrafine meander lines are employed to form the metasurface patterns. Despite the opacity of RF switches, the small size of 0.6 × 0.3 mm² minimizes their visual impact. The excellent visible transparency of the fabricated prototype is demonstrated in Fig. 5a, where the landmark building behind is distinctly visible. The average visible transmittance of the prototype is measured as 53.6% using the LS116 light transmittance meter, as shown in Fig. 5b. To further analyze the visible spectrum, a Shimadzu UV-3600i Plus UV/Vis/NIR spectrometer is used to measure the transmittance spectrum across the wavelengths from 200 to 800 nm. As shown in Fig. 5d, the maximum visible transmittance reaches 62% at 513 nm, indicating excellent visible transparency.

### Energy efficiency performance

High energy efficiency is a fundamental pursuit in window technology, aligning with global sustainable principles. A compact solar panel is integrated to achieve self-powering, eliminating the external power dependency. Combined with the thermal regulation of the EL layer, the green-smart window stabilizes indoor environments, saving the energy consumed by the temperature-control equipment. Furthermore, the reconfigurable RF characteristics can intelligently improve the O2I/I2I communication reliability, thereby reducing the power burden on both the base station and user equipment.

The high energy efficiency of the prototype is realized due to the ultra-low-power design in both the RF switches and digital circuit in the RFID tag, with the measured dynamic power consumption of 19.8 mW during operation and the static power consumption at a microwatt level. Therefore, a compact solar panel with a rated voltage of 5.5 V is sufficient to power the whole prototype. The generated electricity passes through the charging management IC that contains a low-dropout regulator module with an output voltage of 5 V, and the buck-boost converter with a conversion efficiency of approximately 93%. Consequently, the overall efficiency of the digital circuit reaches up to 77%.

**Fig. 6:**
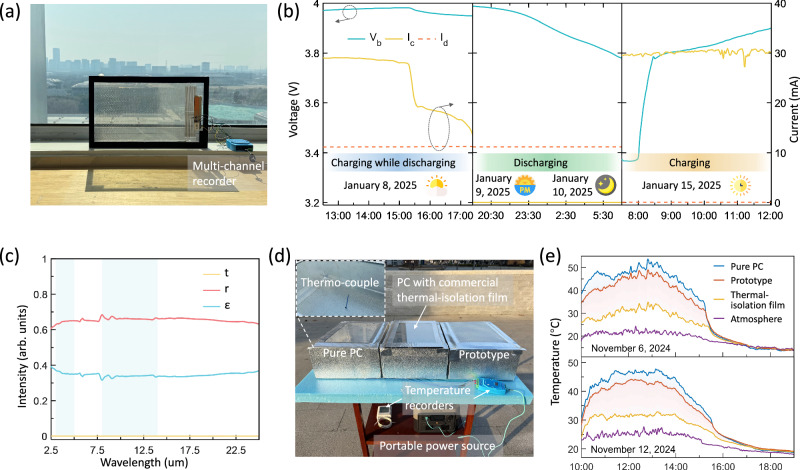
Self-powering and IR performance of the green-smart window. **a** The experimental setup to measure the battery voltage *V*_*b*_, charging current *I*_*c*_, and discharging current *I*_*d*_. **b** The measured *V*_*b*_, *I*_*c*_, and *I*_*d*_ in three periods. **c** The measured IR transmittance *t*, reflectance *r*, and emissivity *ε* from 2.5 to 25 μm. The shaded areas correspond to the results in 3–5 μm and 8–14 μm. **d** The outdoor measurement setup to measure the temperature variations. **e** The measured in-cavity temperature variations of different samples.

To verify that the proposed powering strategy can empower the green-smart window over long term, a measurement setup is constructed, as depicted in Fig. 6a. A multi-channel recorder is used to monitor the voltages across the rechargeable battery (*V*_*b*_), the charging current (*I*_*c*_), and the discharging current (*I*_*d*_). The battery capacity and *V*_*b*_ are in a positive relationship. Specifically, the open circuit voltage of the battery is 4 V when fully charged and 2.7 V when depleted. Thus, we record *V*_*b*_ to characterize the battery capacity. Three experiments are carried out to test the self-powering capability. In the first experiment, the prototype is configured in the RFTE mode while being powered by natural solar energy during the daytime. As shown in Fig. 6b, *V*_*b*_ is stable around 3.98 V, and *I*_*c*_ is larger than *I*_*d*_, indicating that the net power consumption of the prototype is nearly zero. The second measurement aims to test the battery consumption in the RFTE mode overnight. As illustrated in Fig. 6b, *V*_*b*_ drops by only 0.2 V (from 3.98 V to 3.78 V) after operation for 12 h without charging, showing that the prototype can endure long periods of weak or no sunlight. The third experiment is to measure the charging rate in the SRFS mode (no dynamic power consumption) under daylight conditions. As depicted in Fig. 6b, the battery voltage *V*_*b*_ increases by 0.6 V (from 3.3 to 3.9 V) within 4 h, demonstrating the rapid charging capability. Thus, the advantages of zero net power consumption, rapid charging, and resilience to weak or absent sunlight are effectively validated.

### Thermal regulation

Research indicates that the energy loss through doors and windows accounts for nearly half of a building’s total energy consumption and approximately 20% of the overall societal energy consumption^49^. Hence, low-E windows emerge as a green solution because they can block the external IR radiation and reduce the thermal loss in a cost-effective and easy-deployment manner.

To experimentally verify the low IR emissivity, we measured reflection, transmittance, and emissivity of the prototype in the long-wavelength infrared radiation range (LWIR, 2.5–25 μm), denoted as *r*, *t*, and *ε*, by using a Fourier transform infrared spectrometer (Nicolet IS50). Based on Kirchhoff’s law, the IR emissivity can be calculated *ε* = *α* = 1- *r*- *t*, where *r*, *t*, *α*, and *ε* are power quantities. The measured emissivity *ε* in the LWIR range is plotted in Fig. 6c. The average IR emissivity across the LWIR range is calculated as 0.34. Given that the emissivity within the atmospheric windows of 3–5 μm and 8–14 μm is more significant in the IR detection and indoor heating, the average emissivity in the two bands is calculated as *ε*_*3-5um*_ = 0.35 and *ε*_*8-14um*_ = 0.33, respectively. The results indicate that the prototype has the capability for thermal insulation and the potential for reducing indoor energy consumption.

To evaluate the thermal insulation properties of the prototype, we built up outdoor field measurement setups to measure the temperature variations on November 6, 2024, and November 12, 2024. The measurement setups are depicted in Fig. 6d, where three devices under test (DUTs), including reference device #1 (REF #1): a bare PC plate, the prototype, and reference device #2 (REF #2): a PC plate with a commercial thermal insulation film (CTI film), are placed on the top of three cavities of the same size, respectively. The bottom and lateral sides of the cavities are constructed of 10-cm-thick insulation foams. K-type thermocouples and a multi-channel recorder are used to continuously sense and record the in-cavity temperature at regular intervals.

The measured results are presented in Fig. 6e. The maximum in-cavity temperatures of the DUTs of REF #1, prototype, and REF #2 are measured as 53.7 °C, 49.0 °C, and 35.1 °C in the first set of experiments, and 47.8 °C, 44.1 °C, and 32.9 °C in the second set, respectively. The minimum in-cavity temperatures are measured as 13.6 °C, 13.9 °C, and 14.1 °C in the first set, and 18.7 °C, 19.1 °C, and 18.8 °C in the second set, respectively. The largest temperature differences between the prototype and bare PC plate are 7.7 °C and 4.3 °C, occurring at 10:40 on November 6, 2024, and 11:32 on November 12, 2024, indicating the remarkable cooling effect of the prototype upon the indoor environment. We acknowledge that the designed EL layer exhibits inferior thermal performance compared to the CTI film. However, the average visible transmittance of the CTI film is only 16.2% (see Supplementary Note 9 for details). Moreover, it substantially blocks the transmission across the entire microwave bands, thereby impeding the normal O2I wireless communications and potentially causing weak or even non-existent communication links.

### RF performance

To experimentally validate the RF performance of the prototype, we use a pair of lens antennas for transmitting and receiving Gaussian beams to measure the S-parameters (see Supplementary Note 10 for details). When the focal spots of the lens antenna align with the prototype, the measurement and simulation boundary conditions are nearly identical, enabling the measured S_21_ parameter to reliably characterize the RF transmission performance. For TE polarization, the measured results under coding sequences of ‘11’, ‘00’, ‘01’, and ‘10’ are displayed in Fig. 3a, f, j, and n, respectively; the corresponding results for TM polarization are presented in Fig. 3d, h, l, and p, respectively. The comparison between the simulated and measured results reveals a minor discrepancy, characterized by a blue shift of 0.3 GHz and slight transmission amplitude differences, as shown in Figs. 2 and 3. The average error between simulation and measurement is quantified as ${E}_{r}=\tfrac{1}{18}{\sum }_{\theta \in \{0,10,{{\mathrm{..}}}.,80\}}{t}_{mea}^{te}(\theta )+{t}_{mea}^{tm}(\theta )-{t}_{sim}^{te}(\theta )-{t}_{sim}^{tm}(\theta )(dB)$. The calculated average errors in the RFTE and SRFS modes are −0.42 dB and 6.8 dB, respectively. The error discrepancy can be explained by different resonant properties of the RFTE and SRFS modes (see Supplementary Note 11 for details). The non-ideal measured results can be attributed to the measurement limitations and imperfections during fabrication and assembly, including coating preparation, component soldering, and lamination of multilayer structures.

To validate the transmission enhancement of the RFTE mode, the transmission amplitudes of the prototype, RFF #1 (bare PC plate), RFF #2 (a PC plate coated with CTI film), and RFF #3 (PC plate coated with EL layers) are compared (see Supplementary Note 12 for details). Key transmission performance characteristics of these DUTs are summarized in Table 1. Additionally, the broadband performance of the prototype across the frequency range of 1–8 GHz is evaluated. The results indicate that, through mode switching, the designed window can maintain the transmission amplitude higher than −5 dB over 1.1–8 GHz to support diverse communication bands, and enable selective RF shielding in two narrow bands without affecting the quality of the out-of-band O2I links (see Supplementary Note 13 for details).

**Table 1:** Key RF transmission characteristics of the DUTs

DUTs		0°	80° (TE)	80° (TM)
Prototype (RFTE)	Simulation	0.94 (−0.54 dB)	0.73 (−2.73 dB)	0.81 (−1.8 dB)
	Measurement	0.90 (−0.92 dB)	0.66 (−3.6 dB)	0.86 (−1.3 dB)
Prototype (SRFS)	Simulation	0.06 (−24.4 dB)	0.011 (−39.2 dB)	0.18 (−14.9 dB)
	Measurement	0.10 (−20.1 dB)	0.036 (−28.9 dB)	0.35 (−9.1 dB)
RFF #1 (bare PC plate)	Simulation	0.94 (−0.50 dB)	0.45 (−6.97 dB)	0.83 (−1.62 dB)
	Measurement	0.92 (−0.82 dB)	0.41 (−7.74 dB)	0.84 (−1.51 dB)
RFF #2 (PC + CTI film)	Simulation	\	\	\
	Measurement	0.06 (−22.2 dB)	0.04 (−26.5 dB)	0.11 (−18.3 dB)
RFF #3 (PC + EL layer)	Simulation	0.89 (−1.03 dB)	0.31 (−10.25 dB)	0.83 (−1.63 dB)
	Measurement	0.88 (−1.11 dB)	0.38 (−8.4 dB)	0.79 (−2.05 dB)

**Fig. 7:**
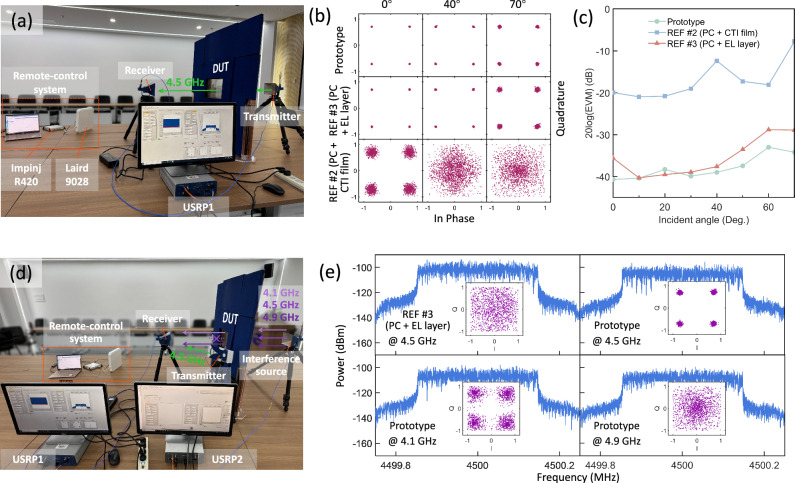
Experimental results for wireless communications. **a** The measurement setup to validate the O2I wireless communications. **b** The measured constellation diagrams at incident angles of 0°, 40°, and 70° for the prototype, REF #2, and REF #3. **c** The measured EVM in the angular range from 0° to 70° of the prototype, REF #2, and REF #3. **d** The measurement setup to validate the I2I wireless communications. **e** The measured constellation diagrams and power spectra for the prototype and REF #1.

Two sets of communication experiments are conducted to evaluate wireless enhancements achieved by the proposed green-smart window. The O2I communication scenario is presented in the first set of experiments, where two horn antennas representing an outdoor transmitter and an indoor receiver are positioned on either side of the DUT, as shown in Fig. 7a. The RF signals received by the receiving antenna characterize the communication quality. The windowed absorbing screen embedded with DUT is rotated on the horizontal plane from 0° to 70° in a step of 10°. In the meantime, the power spectrum and received constellation diagram are measured at each angle. Three DUTs are measured, namely, the prototype, REF #2, and REF #3. As illustrated in Fig. 7b, the received constellation diagrams of the prototype are tightly clustered at all angles of 0°, 40°, and 70°, while those of REF #3 gradually become scattered as the incident angle increases. Since the commercial thermal-isolation film shields the transmission in the entire RF band, the received constellation diagrams of REF #2 are severely cluttered at all incident angles. The error vector magnitude (EVM) is an indicator to evaluate the quality of the modulation signal, and the measured results are illustrated in Fig. 7c. It is found that the prototype shows a remarkable improvement in the signal quality compared with REF #2 and #3 in wide-angle communications.

The I2I communication scenario is exhibited in the second set of experiments, as shown in Fig. 7d. A receiving horn antenna and a Vivaldi antenna are placed on one side of the windowed absorbing screen, representing the indoor receiver and transmitter, respectively. A horn antenna representing the interference source is placed on the other side. The communication signals and co-channel interference share the same modulation type and bandwidth. Their transmit powers are set to 20 dBm and −15 dBm, respectively, with different baseband information. When REF #3 is under test, the received constellation diagrams at 4.5 GHz are greatly distorted, as shown in Fig. 7e. When DUT is the prototype, the received constellation diagram at 4.5 GHz exhibits very high quality, as required for normal communications. However, at 4.1 and 4.9 GHz, the diagram becomes cluttered, demonstrating the effective SRFS capability. Notably, the average of received powers across four cases shown in Fig. 7e remains nearly identical, suggesting that even weak co-channel interference can significantly affect the quality of I2I communication. Fortunately, the proposed green-smart window can effectively protect the I2I communication from outdoor co-channel interference, while maintaining strong O2I wireless links outside the frequency band.

## Discussion

This study presents a metasurface-enabled green-smart window designed for wide-angle wireless communications and energy conservation. The prototype achieves external-power-free operation through a compact solar panel, enabling environment-friendly support for three reconfigurable functions (SRFS, RFTE, polarization filtering) across wide incidence angles from 0° to 80° under dual polarizations. A 41-m control range is realized via backscattering of an RFID tag, permitting wireless function switching through reader-initiated commands. Experimental results demonstrate high visible transparency, low IR emissivity, and reconfigurable RF responses that align well with the simulation outcomes. The capabilities of self-powering and thermal regulation highlight the potential of green-smart windows for energy-efficient and green cities. Two sets of communication measurements indicate that the green-smart window can maintain high O2I communication quality even in large incident angles, and protect I2I communications from outdoor co-channel interference without compromising the out-of-band signal integrity. The proposed design extends the scope of green-smart windows, positioned as pivotal technologies for future communication networks and global sustainability.

## Methods

### Meta-atom design

The meta-atom consists of two composite film coatings and a 5-mm-thickness polycarbonate (PC) plate with dielectric constant *ϵ*_*r*_ = 2.65(1- j0.001) sandwiched between them, as shown in Fig. 2c. The composite film coating is composed of a reconfigurable metal pattern layer (RMP layer) that enables switchable RF responses, and an etched low-E layer (EL layer) that provides the thermal insulation. The metasurface patterns of the RMP layer are printed on a 0.125-mm-thickness polyethylene terephthalate (PET) film with dielectric constant *ϵ*_*r*_ = 3(1- j0.03). Created from the metallic meander lines with a linewidth of 50 μm, the metasurface patterns can be modeled as large-value inductors, and the inductance values can be flexibly adjusted by changing the geometric parameters. The ARW3171 switch, a single-pole single-throw switch using complementary metal oxide semiconductor technology, is selected as the active component due to its small size, low cost, high isolation, and ultra-low power consumption. Each RMP layer on the top and bottom incorporates an ARW3171 switch connected in series with the meander-line resonators.

To realize the RF characteristics as expected by the TL model, a comprehensive parameter analysis is conducted through full-wave simulation using commercial simulation software CST Microwave Studio 2022. The central operation frequencies of both the RFTE and SRFS functional modes are mainly determined by the geometric parameters of the RMP layer, including the periodic size *P*, the substrate thickness *H*_*pc*_, and the meander-line lengths *l*_*1*_ and *l*_*2*_. With appropriate geometric parameters, the operational bandwidths of the two functional modes achieve spectral overlap, and this capability can adapt to diverse substrate thicknesses via geometric parameter tuning. Additionally, the sheet resistance *R*_*ITO*_ and the width of the etched gap *w*_*g*_ of the EL layers are analyzed and optimized. Detailed parameter analysis of the RMP and EL layers can be found in Supplementary Note 2. Under the optimized parameters of *P* = 9 mm, *l*_*1*_ = 4.5 mm, *l*_*2*_ = 2.25 mm, *w*_*g*_ = 0.16 mm, *H*_*pc*_ = 5 mm, *R*_*ITO*_ = 12 Ω/sq, the extracted surface impedance of the meta-atom approaches the theoretical expectation (see Supplementary Note 3 for details).

### Fabrication prototype

To evaluate the performance of the green-smart window, a prototype consisting of 23 × 35 meta-atoms and covering an area of 238 × 410 mm^2^ is fabricated, as shown in Fig. 5a. The photographs of the fabricated RMP and EL layers are displayed in Fig. 5c. The RMP layers are fabricated by the flexible printed circuit technology, and the EL layers are created from the etched ITO film with square holes arranged periodically. The microscope images in the right panel of Fig. 5c demonstrate the structural parameters of the prototype in detail.

Surface-mounted technology is utilized to precisely solder the ARW3171 switches onto the pads on the RMP layer. Low-temperature solder is employed to ensure that the PET film is not deformed or warped during the soldering process. Subsequently, the RMP and EL layers are stacked to form a composite film coating. Then, the composite film coatings are attached to both sides of the PC plate carefully. The RFID tag and the solar panel are compactly co-integrated within a shared aperture and positioned along the edge of the window, as shown in the insets in Fig. 5a.

### Measurement setup

Two measurement setups are constructed to evaluate the enhancement in O2I communication and the anti-interference performance in I2I communication achieved by the prototype, as shown in Fig. 7a, d, respectively. In both experiments, the DUTs are embedded in a windowed absorbing screen to mitigate the non-line-of-sight transmissions, thereby ensuring measurement accuracy. All antennas (e.g., transmitter, receiver, and interference source) are positioned 1 meter from the windowed absorbing screen. A remote-control system, consisting of a UHF RFID reader (Impinj R420), a personal computer, and a circular-polarity UHF antenna (Laird 9028), is situated away from the DUTs to facilitate wireless remote control. Prior to the measurement, I/O control words are transmitted to the RFID tag to configure the prototype into the desired functional mode. During the measurement, a universal software radio peripheral (USRP-2974, National Instruments Corp.) is used to modulate and demodulate the RF signals transmitted and received by the horn antennas. The QPSK signals, with a bandwidth of 100 MHz, are generated by the USRP and subsequently received by the receiving antenna after passing through the DUTs. The power spectral density and constellation diagram are used as the primary metrics to assess the communication link quality.

## Supplementary information

Supplementary Information

Transparent Peer Review file

## Data Availability

The data supporting the findings of this study are presented in the paper and in the Supplementary information, or can be made available upon request by contacting the corresponding authors.
