# Supplementary material for: A metasurface-enabled green-smart window for intelligent wireless communications with high visible transparency and low infrared emissivity
Source: Nat Commun. 2026 May 13;17:6414. doi: 10.1038/s41467-026-72643-x (PMC13376189; doi:10.1038/s41467-026-72643-x)
Supplement: Supplementary file 1 — Supplementary Information [file 41467_2026_72643_MOESM1_ESM.pdf]

Supplementary Information for

A metasurface-enabled green-smart window for intelligent wireless communications with high visible transparency and low infrared emissivity

Rui Zhe Jiang[1,2†], Chuan Kui Shen[4†], Hui Dong Li[1], Qun Yan Zhou[1], Zhi Hui Fu[1], Zheng Xing Wang[3], Terry Tao Ye[4,5], Jun Yan Dai[1], Lie Kun Yang[4], Jitong Ma[6], Qiang Cheng[1,*] and Tie Jun Cui[1,7,*]

[1] State Key Laboratory of Millimeter Waves, Southeast University, Nanjing 210096, China

[2] Zhangjiang Laboratory, 100 Haike Road, Pudong, Shanghai 201210, China

[3] State Key Laboratory of Terahertz and Millimeter Waves, City University of Hong Kong, Hong Kong 999077, China

[4] Southern University of Science and Technology Jiaxing Research Institute, Jiaxing 314031, China

[5] School of Science and Engineering, The Chinese University of Hong Kong, Shenzhen 518172, China

[6] School of Information and Science, Dalian Maritime University, Dalian 116026, China

[7] Suzhou Laboratory, Suzhou 215004, China

[†] These authors contributed equally: Rui Zhe Jiang, Chuan Kui Shen.

E-mail: qiangcheng@seu.edu.cn and tjcui@seu.edu.cn

The Supplementary Information includes:

Supplementary Note 1. Feasibility of Multispectral Manipulation
Supplementary Note 2. Parameter Analysis of the RMP and EL Layers
Supplementary Note 3. Extracted Surface Impedance of the Meta-Atom
Supplementary Note 4. Equivalent Circuit Model of the Meta-Atom
Supplementary Note 5. Capacity of Amplitude Modulation
Supplementary Note 6. System Configurations for Remote Control and Self-Powering
Supplementary Note 7. Experimental Setup for the Designed RFID Tag
Supplementary Note 8. Effectiveness of the Transparent Absorber
Supplementary Note 9. Visible Transmittance of the Bare PC Plate and CTI Film
Supplementary Note 10. Experimental Setups to Measure the RF Transmissions
Supplementary Note 11. Resonant Properties of the RFTE and SFRS Modes
Supplementary Note 12. Transmission Enhancement in the RFTE Mode
Supplementary Note 13. Broadband characteristics across 1-8 GHz

Supplementary Note 1. Feasibility of Multispectral Manipulation

1.1 Feasibility of Switching Between the RFTE and SRFS Modes

Achieving high RF transmission amplitude under wide-angle incidence using planar structures remains a fundamental challenge in electromagnetic (EM) engineering, particularly in passive operation regimes[1-4]. In parallel, there has been growing interest in the development of planar structures capable of static and selective RF shielding, especially in the field of flexible/transparent materials[5-7]. To the best of our knowledge, this is the first time that the dynamically switchable functions between wide-angle high transmission and wide-angle selective shielding have been systematically explored and experimentally validated.

To begin with, we assume that the top and bottom layers of the meta-atom are loaded with orthogonal active components to realize independent control of dual polarization, since it holds great practical value in urban environments, where the spatial waves are arbitrarily polarized due to the multipath fading. The corresponding general dual-layered TL model is established, as depicted in **Fig. S1**a. Under illumination of the TE-polarized waves, the top-layer coating, comprising a passive structure and tunable components, is modeled as an ideally adjustable and lossless shunt impedance (Z_a = R_a + jX_a, where R_a = 0 and X_a is adjustable). The bottom-layer coating is modeled as a fixed lossless shunt impedance (Z_f = R_f + jX_f, where R_f = 0), since the tunable components on the bottom layer are inactive under TE-polarized. For TM polarization, the order of the reactances Z_a and Z_f is reversed; however, the transmission coefficients remain unchanged due to the reciprocal nature of the structure.

The detailed configuration information of the TL model is shown follows. The substrate is equivalent to a TL with impedance $Zs = Z_0/\epsilon_s$, where $Z_0 \approx 377\ \Omega$ is the impedance of the air and ϵ_s is the relative dielectric constant of the substrate. Based on the well-established transfer matrix method, the transmission coefficient $t(f_0, H_s, \epsilon_s, X_a, X_f, \theta_i)$ of the TL model can be quickly calculated[8,9]. For the convenience of analysis, the central operating frequency f_0 is set to 4.2 GHz as a typical frequency for 5G networks in the Sub-6 band and satellite communication in the C-band. The thickness H_s and dielectric constant ϵ_s are set to 5 mm and 2.65, respectively, typical for high-strength PC boards. The incident angle is denoted as θ_i. In

this case, the independent variables of $t(f_0, H_s, \epsilon_s, X_a, X_f, \theta_i)$ are narrowed down to X_a, X_f, and θ_i.

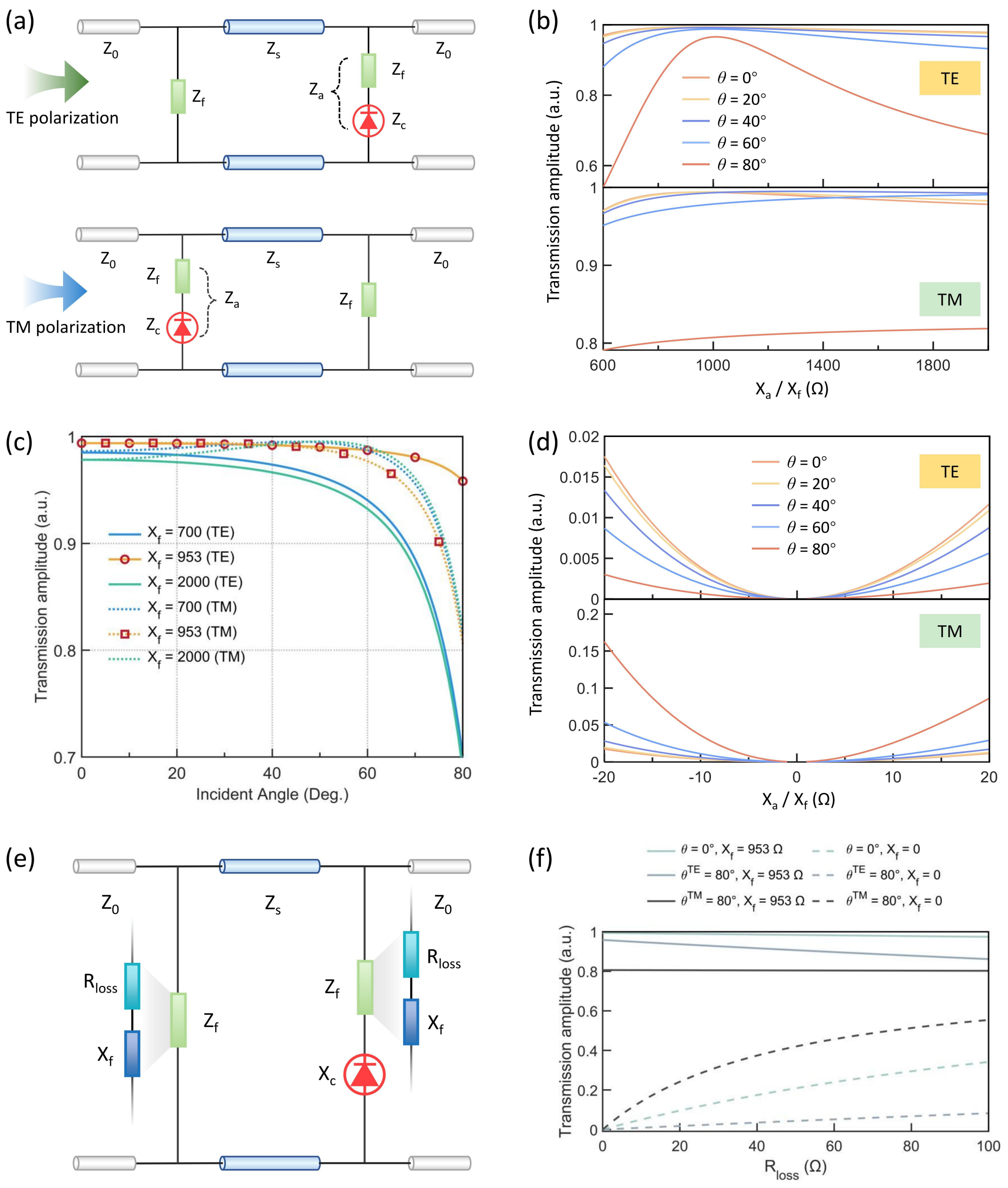

Fig. S1. (a) The TL model of the proposed meta-atom under x- and y-polarizations. (b) The transmission amplitudes when X_a ($X_a = X_f$) is from 600 Ω to 2000 Ω. (c) Angular performance regarding X_a ($X_a = X_f$) is at 700 Ω, 953 Ω, and 2000 Ω, respectively. (d) Transmission amplitudes when X_a ($X_a = X_f$) is from -20 Ω to 20 Ω. (e) TL model with the introduction of the loss factor. (f) Transmission amplitudes regarding various R_{loss}.

The transmission amplitudes under angles θ_i = 0°, 20°, 40°, 60°, and 80° are calculated when the reactances X_a and X_f vary froam -1500 to 1500 Ω, as shown in **Fig. 2**b (main text).

Firstly, due to the reciprocity of the structure, the transmission amplitudes are symmetric about the line X_a = X_f, and the maximal transmission amplitude occurs on this line. This indicates that similar passive structures can be adopted in the top and bottom layers to ensure near-perfect transmission. Additionally, it is found that the reactance values required for near-perfect transmission and near-total shielding are almost unchanged against incident angles, which indicates that wide-angle insensitivity is theoretically achievable. In addition, near-perfect transmission and near-total shielding can be switched by only changing X_a while X_f is fixed, as shown by the two-way arrow, indicating the feasibility of the reconfigurable transmission.

Next, we begin to explore the appropriate reactance values of the fixed coating, the adjustable coating for RFTE, and the adjustable coating for SRFS, respectively denoted as X_f, X^a_{RFTE} , and X^a_{SRFS}, to ensure the wide-angle transmission t_{RFTE} (X_f, X^a_{RFTE}, θ_i) is as high as possible and t_{SRFS} (X_f, X^a_{SRFS}, θ_i) is near zero. The reactance X^a_{RFTE} = X_f + X^c_{RFTE}, where X^c_{RFTE}, represents the reactance of the tunable components. As discussed earlier, $X^c_{RFTE} \approx 0$ ($X_f \approx X^a_{RFTE}$) is necessary to maximize the transmission amplitude, and t_{RFTE} (X_f, X^a_{RFTE}, θ_i) is hereafter simplified as t_{RFTE} (X_f, θ_i). The relationship of t_{RFTE} (X_f, θ_i) between angles and polarizations is plotted in **Fig. S1**b. The search for X_f to maximize t_{RFTE} (X_f, θ_i) can be formulated as an optimization problem, defined by:

$$\mathrm{X}_f^* = \arg\max_{X_f} \sum_{\theta_i=0}^{80} (t_{RFTE}^{TE}(X_f, \theta_i) + t_{RFTE}^{TM}(X_f, \theta_i)), \tag{1}$$

where X_f^* represents the optimized solution, and the optimization results indicate that X_f^* is 953 Ω. To verify the effectiveness of the optimization, **Fig. S1**c compares the tra nsmission amplitudes against different incident angles when X_f is set as 700, 953, and 2000 Ω, respectively. It is found that t_{RFTE} (X_f^* = 953 Ω, $\theta^{TE/TM}$) achieves the highest t ransmission amplitude across the metrics of incident angle and polarization mode. For t he SRFS mode, the condition of $X^{a\ S}_{RFS}$ = 0 is required to realize total reflection, as sho wn in **Fig. S1**d. According to TL theory, this condition induces series resonance with near-zero reactance at the target frequency, effectively mimicking a metal ground that blocks incident waves and suppresses transmission. Due to the inherent frequency-dispe

rsive characteristics of the series resonance, frequency-selective RF shielding is theoreti cally achievable.

In practical implementations, the loss factors are inevitable due to the non-ideality of the materials, components, and fabrication. To characterize the impact of the loss factors on the RF transmission, series resistors R_{loss} are introduced into the TL model, as shown in **Fig. S1**e. The calculated transmission amplitudes regarding various R_{loss} are illustrated in **Fig. S1**f. It is found that both t_{RFTE} and t_{SRFS} suffer slight deterioration as R_f increases. Therefore, to minimize the loss level, the metal structure is made of tinned copper lines, and the low-loss tunable components (ARW3171 switches) are employed in our design.

1.2 Feasibility of Achieving High Visible Transparency

The visible transparency level of the proposed green-smart window is mainly determined by two independent factors: material selection and metasurface structure design.

1) **Material selection**

Substrate materials: To balance cost-effectiveness and fabrication simplicity, commercially available transparent polymers and glass are prioritized as metasurface substrates, such as polycarbonate (PC), polymethyl methacrylate (PMMA), quartz, and borosilicate. Among them, PC stands out in security-critical applications due to its exceptional impact resistance, lightweight nature (density ~1.2 g/cm^3), and excellent UV/weathering stability.

Transparent conductive materials: Conventional transparent resistive films such as indium tin oxide (ITO), metal meshes, metallic nanowires, and dielectric-metal-dielectric (DMD) multilayers are widely adopted for transparent electrodes. However, these materials inherently exhibit an inverse correlation between electrical conductivity and visible transparency. This trade-off originates from free-carrier absorption and plasma resonance effects—increasing charge density enhances electrical conductivity but simultaneously elevates photon-electron interactions, leading to significant light attenuation in the visible spectrum.

2) **Metasurface structure**

As discussed in Note 1.1, the proposed TL model indicates that the passive structure of the metasurface coating should possess a high inductance value ($Z_f = R_f + jX_f$, X_f is expected to be 953 Ω). According to frequency selective surface theory, periodic metallic-line structures

demonstrate high inductive reactance characteristics when illuminated by EM waves polarized parallel to their longitudinal axis[10]. Directly replacing the metallic-line structure with transparent conductive materials will lead to suboptimal performance in both RF and visible bands. To address this problem, the metallic portion of the meta-atom is redesigned as a sparsely arranged ultrafine meandering structure. This approach enables excellent performance in both RF and visible spectra simultaneously. High conductivity can be achieved through the flexible printed circuit technology, in which the metal thickness is 18 μm (far larger than the skin effect). Although the existing tunable components are all opaque, the miniature size of the 0201 packaging can be selected to minimize their impact on visible transparency.

1.3 Feasibility to Achieve Low IR Emissivity

A cost-effective approach for achieving low IR emissivity utilizes the low-E layers, with the ITO film emerging as the primary option due to the good balance between electrical conductivity, optical transparency, and IR emissivity[11-13]. To evaluate the effect of the ITO films on RF performance, shunt impedances Z_{ITO} representing the ITO film are incorporated into the TL model, as shown in **Fig. S2**a. As plotted in **Figs. S2**b,c, the calculation results indicate that the ITO film with a low resistance component R_{ITO} ($Z_{ITO} = |Z_{ITO}|e^{j\phi_{ITO}}$, $R_{ITO} = Z_{ITO} \cos\phi_{ITO}$) significantly degrades the RF performance of the meta-atom. Conversely, an ITO film with high R_{ITO} typically exhibits poor thermal isolation properties. It is noted that the condition of $R_{ITO} < 30$ Ω/sq is necessary to ensure low IR emissivity, while $R_{ITO} < 10$ Ω/sq is particularly beneficial for efficient thermal regulation[14]. Consequently, the raw ITO films must be precisely processed to get the optimal tradeoff between RF and IR performance.

Etching the raw ITO films with narrow slots can reduce the surface impedance effectively with little sacrifice on the IR performance. To quantify this, a surface with a sheet impedance of 12 Ω/sq is modelled in full-wave simulations to examine the relationship between the extracted surface impedance $|Z_{ITO}|$ and etched gap width w_g. The method to extract the surface impedance is detailed in Note 3. As illustrated in **Fig. S2**d, when w_g becomes wider, $|Z_{ITO}|$ greatly increases since the continuous surface currents are cut off by the narrow slots, and the filling ratio gradually decreases. The effective emissivity of the low-E film is calculated by:

$$\varepsilon_{effective} = \varepsilon_{PC} f_{PC} + \varepsilon_{ITO} f_{ITO}, \tag{2}$$

where ε_{PC} and ε_{ITO} are the emissivities of the PC plate and ITO film, respectively, and f_{PC} and f_{ITO} are their corresponding area fractions after etching. As a compromise, the etched ITO film with R_{ITO} = 12 Ω/sq and w_g = 0.16 mm is selected in the design, corresponding to Z_{ITO} = 17 - j2578 Ω (2578 $e^{-j89.6}$ Ω) and the filling ratio of 74%.

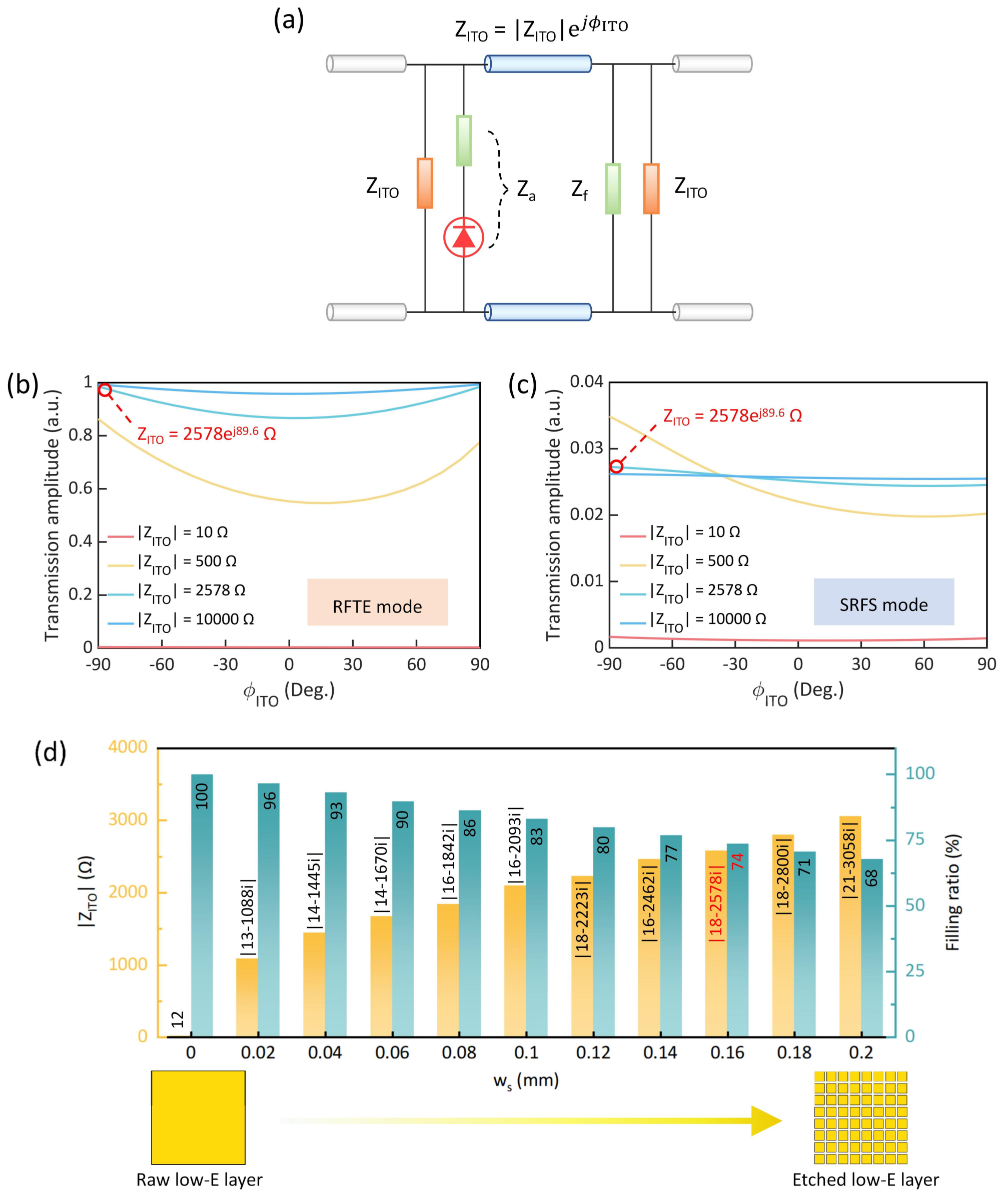

Fig. S2. (a) TL model of the proposed meta-atom with the introduction of ITO film. (b,c) Transmission amplitudes regarding various impedance Z_{ITO} ($Z_{ITO} = |Z_{ITO}| e^{j\phi_{ITO}}$)when the meta-atom is in the modes of (b) RFTE and (c) SRFS, respectively. (d) Relationship between the extracted Z_{ITO} through full-wave simulation, the filling ratio of the ITO materials, and the etched gap width w_g.

Supplementary Note 2. Parameter Analysis of the RMP and EL Layers

To accelerate the parametric optimization, parameter analysis on the meta-atom is conducted. For the RMP layer, key geometric parameters, includingthe periodic size P, the thickness of the substrate H_{pc}, and the sizes of the meandering lines (l_1 and l_2), are analyzed. **Figs. S3**a-d demonstrate the simulated transmission amplitudes under normal incidence when one of the geometric parameters is changed while the others are fixed. It can be observed that the central frequencies of the RFTE and SRFS modes can be effectively changed by tuning the geometric parameters. This flexible tunability offers two main advantages: 1) The central operation frequency of the green-smart window can be tuned as required by different communication bands. 2) The design can be applied to various substrate thicknesses. As illustrated in **Fig. S3**e, the simulation results indicate that the green-smart window performs well with PC substrate thicknesses of 5 mm, 7 mm, and 9 mm. The corresponding geometric parameters for each case are detailed in **Table S1**.

For the EL layer, the etched gap width (w_g) and the sheet resistance of ITO film (R_{ITO}) are elaborately analyzed and optimized to ensure superior multispectral performance. Parameters of w_g and R_{ITO} that correspond to the best RF performance are remarkably decreased for low IR emissivity through joint optimization of the EL and RMP layers. The transmission amplitude spectra under normal incidence at 4.2 GHz against R_{ITO} and w_g are depicted in **Fig. S4**. The results show that the meta-atom with w_g = 0.16 mm and R_{ITO} = 40 Ω/sq exhibits the best performance in both RFTE and SRFS modes. However, the ITO with R_{ITO} = 40 Ω/sq has underperformed low-E ability. Therefore, a design with w_g = 0.16 mm and R_{ITO} = 12 Ω/sq (see the analysis results in Note 1.3) is ultimately selected, providing low IR emissivity with negligible sacrifice on the RF performance.

Table S1. The geometric parameters of the RMP layer for different H_{pc}

	H_{pc} (mm)	P (mm)	l_1 (mm)	l_2 (mm)
Case 1	5.00	9.00	2.25	2.25
Case 2	7.00	8.50	2.15	1.19
Case 3	9.00	8.00	1.95	0.25

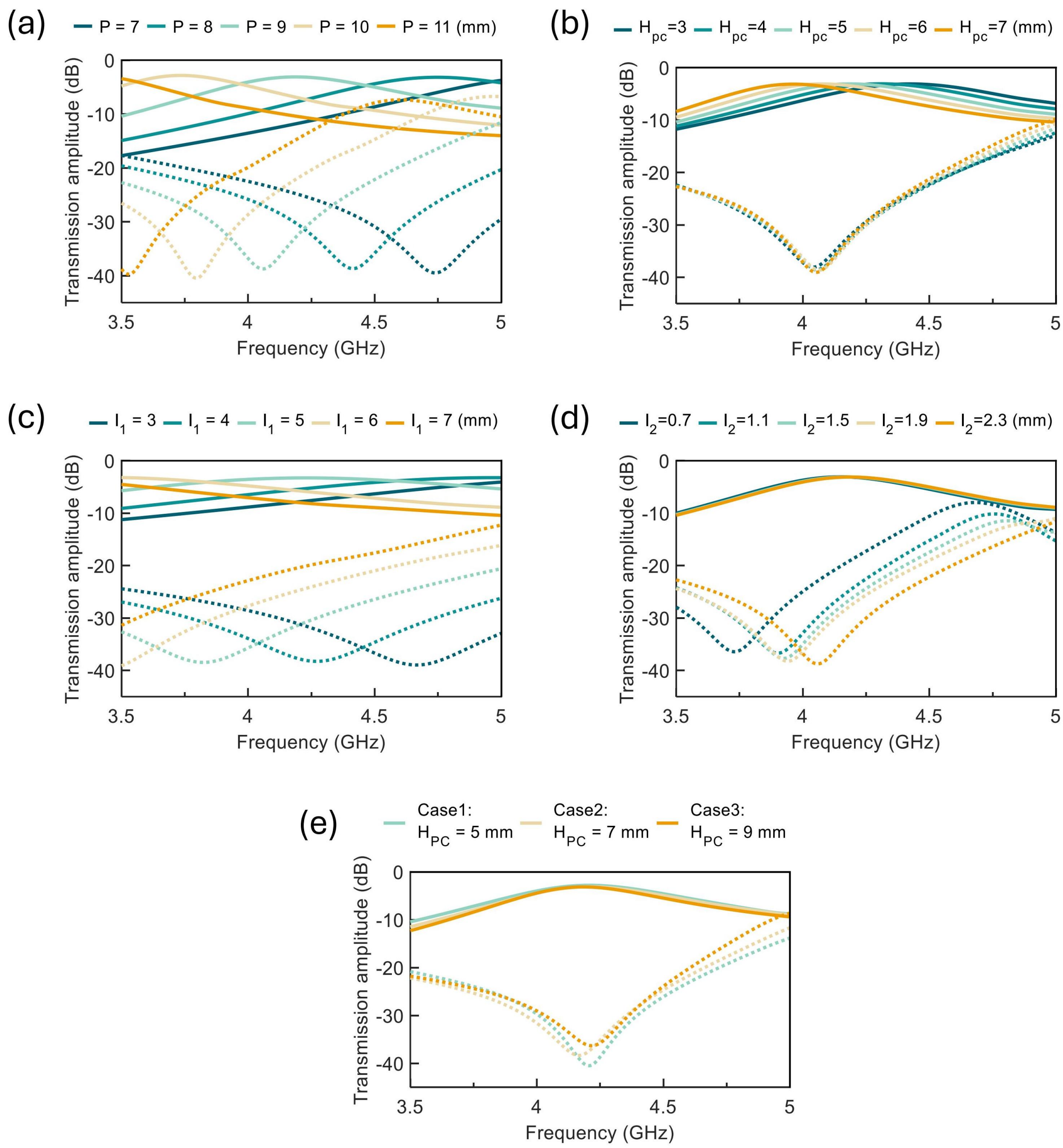

Fig. S3. Parameter analysis on (a) periodic size *P*, (b) PC thickness H_{pc}, (c,d) length of the meandering line l_1 and l_2, respectively. (e) Transmission amplitude spectra in the cases of the H_{pc} = 5, 7, and 9 mm. Solid and dashed lines represent the RFTE and SRFS modes, respectively.

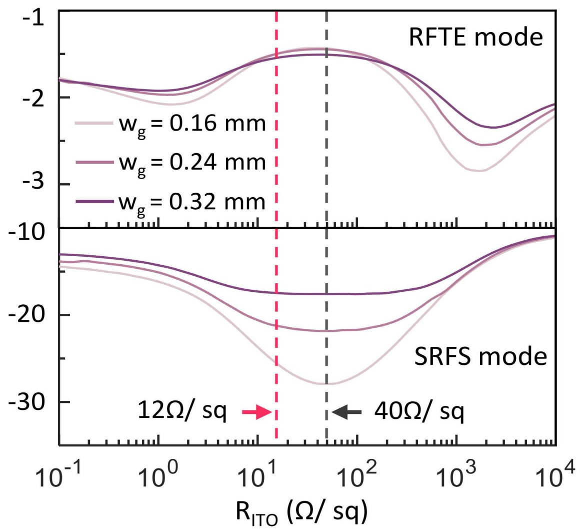

Fig. S4. Simulated transmission amplitude spectra in the normal incidence with different R_{ITO} and w_g in the modes of RFTE and SRFS, respectively.

Supplementary Note 3. Extracted Surface Impedance of the Meta-Atom

In full-wave simulations, the surface impedance of composite film coating $Z_{extracted} = R_{extracted} + jX_{extracted}$ is extracted for parametric optimization. The detailed procedures for extracting the surface impedance of the meta-atom are listed below:

1. Obtain the complete scattering matrix $S_{meta}^{sim} = \begin{pmatrix} S_{11} & S_{12} \\ S_{21} & S_{22} \end{pmatrix}$ of the single-layered meta-atom (PC plate and a single-layered composite coating on the top layer) using full-wave simulation.

2. Transform the scattering matrix $S_{meta}^{sim} = \begin{pmatrix} S_{11} & S_{12} \\ S_{21} & S_{22} \end{pmatrix}$ to the corresponding transfer matrix $M_{meta}^{sim} = \begin{pmatrix} A & B \\ C & D \end{pmatrix}$, where

$$A = \frac{((1+S_{11})(1-S_{22})+S_{12}S_{21})}{2S_{21}}, \tag{3}$$

$$B = \frac{((1+S_{11})(1+S_{22})-S_{12}S_{21})Z_0}{2S_{21}}, \tag{4}$$

$$C = \frac{((1-S_{11})(1-S_{22})-S_{12}S_{21})}{2S_{21}Z_0}, \tag{5}$$

$$D = \frac{((1-S_{11})(1+S_{22})+S_{12}S_{21})}{2S_{21}}. \tag{6}$$

Specifically, Z_0 is the intrinsic impedance of the air.

3. Calculate the transfer matrix of the substrate layer M_{sub}^{cal} using the equivalent TL model, which is determined by the permittivity and thickness of the PC plate.

4. Derive the transfer matrix of the composite film using $M_{film} = M_{meta}^{sim}\,(M_{sub}^{cal})^{-1}$, where $M_{film} = \begin{pmatrix} 1 & 0 \\ Y_p & 1 \end{pmatrix}$. Thus the extracted admittance Y_p can be obtained, and the extracted impedance Z_p can be calculated as $\frac{1}{Y_p}$.

The extracted surface impedances ($Z_{extracted} = R_{extracted} + jX_{extracted}$) are plotted in **Fig. S5**. Both $X_{extracted}$ of the top and bottom coatings at RFTE mode are close to the expected value X_f* =953Ω, showing that the structure design meets theoretical expectations. The slight difference between the $Z_{extracted}$ of the top and the bottom layers results from the fact that the ON-state ARW3171 is not perfectly conductive. Although the loss factors represented by $R_{extracted}$ are nonzero, their effects on the RF performance are minimal (see Fig. S1f in Note 3).

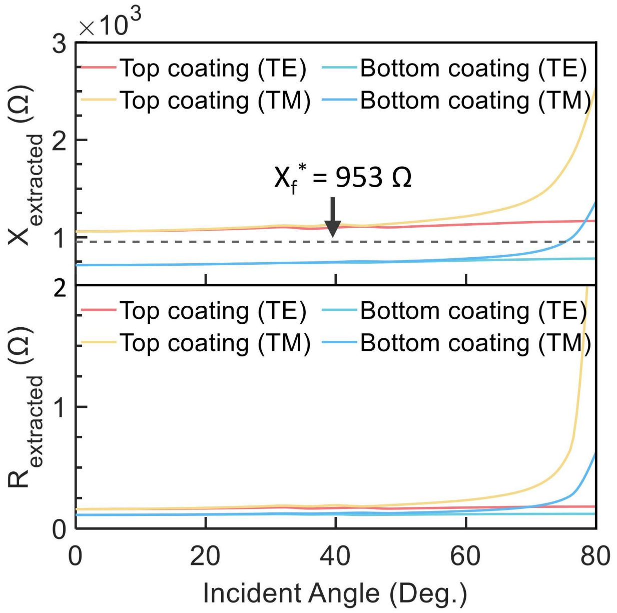

Fig. S5. Extracted reactance $X_{extracted}$ and resistance $R_{extracted}$ of the designed composite film coating under different incident angles.

Supplementary Note 4. Equivalent Circuit Model of the Meta-Atom

An equivalent circuit model is developed to explain the phenomenon of frequency dispersion of the proposed meta-atom. The model structure is illustrated in **Fig. S6**. As marked within the red dashed box, the ARW3171 switch is modelled as a combination of the lumped elements,

where R_d is the tunable resistor. By tuning the R_d from 0 to 50 kΩ, the meta-atom transforms from the SRFS to the RFTE mode. The intrinsic impedances of the air and the PC plate are represented as Z_0 and Z_s. The detailed circuit parameters are R_{ito} = 5 kΩ, R_1 = 13 Ω, R_2 = 4.7 Ω, C_1 = 0.4 pF, C_2 = 0.67 pF, C_3 = 30 fF, C_4 = 68 fF, L_1 = 13 nH, L_2 = 15 nH, L_3 = 1 pH. The calculation results are demonstrated in Fig. 2e in the main text. The close agreement between the calculation and simulation results confirms the accuracy of the proposed model.

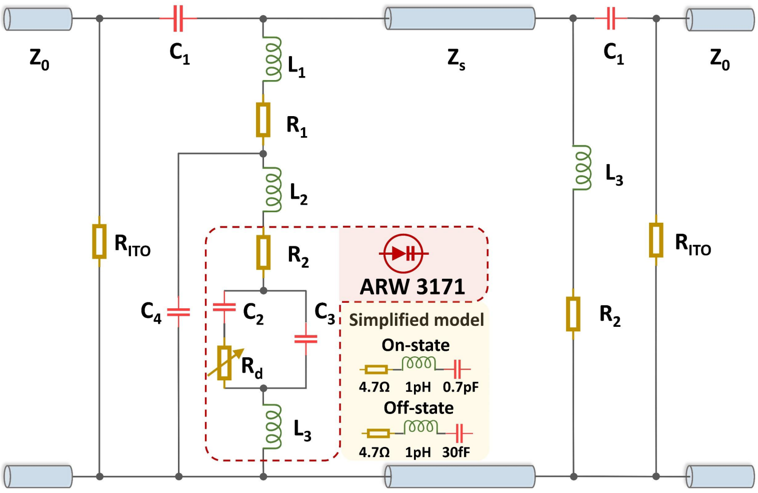

Fig. S6. Equivalent circuit model of the proposed meta-atom.

Supplementary Note 5. Capability of Amplitude Modulation

Measurement results indicate that the ARW3171 switch exhibits an intermediate state (a transition between the 'On' and 'Off' states) when the voltage ranges from 0 V to 1.5 V. Consequently, the RF transmission amplitude can be continuously modulated. As shown in **Fig. S7**, the modulated amplitudes are calculated, simulated, and experimentally measured. **Figs. S7**a-c present the reflection amplitudes, and **Figs. S7**d-f illustrate the transmission amplitudes. All the calculation, simulation, and measurement results consistently validate the capacity of continuous amplitude modulation for both reflection and transmission; thus, indicating the proposed structure can be used as a spatial power divider to allocate the ratio of the reflected and transmitted power in the full space.

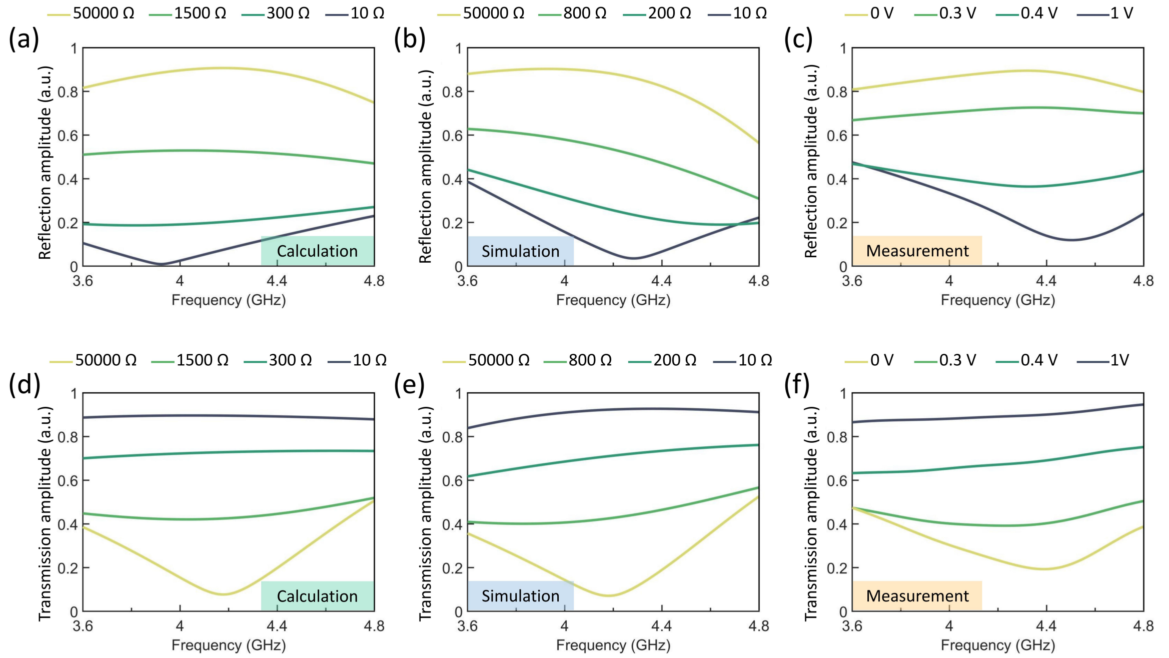

Fig. S7. (a) Calculated, (b) simulated, and (c) measured results of the reflection amplitudes. (d) Calculated, (e) simulated, and (f) measured results of the transmission amplitudes.

Supplementary Note 6. System Configurations for Remote Control and Self-Powering

In our experiments, the key components of the remote-control systems include the UHF RFID reader (Impinj R420) for data interaction with the RFID tag, a personal computer for controlling the RFID reader through the software ItemTest^{TM}, and a circular polarity UHF antenna (Laird 9028) for transmitting and receiving the UHF waves, as displayed in **Fig. S8**a. For ease of debugging, the control system employed in our experiment is not highly integrated at present. However, we also mention that in the subsequent development, handheld RFID readers or even smartphones with control applications can be adopted to enhance portability.

Once the components are properly connected and network interfaces are correctly configured, a Query Command can be sent from the personal computer to establish a communication link between the RFID reader and the EM4325 IC embedded in the RFID tag. Upon successful tag recognition, we can send the I/O control words into the user memory of the EM4325 IC, where the last 4 bits of the word configure the levels of the I/O ports. Take the I/O control word of ‘0x8005’ as an example, the four I/O ports of the EM4325 IC are encoded

in a binary sequence of ‘0101’ (0x5 = 0B0101), where ‘0’ denotes 0 V and ‘1’ denotes 1.8 V, to control the states of the ARW3171 switches on the green-smart window. The software operator interface of ItemTest^{TM} is shown in **Fig. S8**b.

To supply the power consumed by the dynamic components, a solar panel is fixed to the rear of the tag to harvest solar energy. The anode of the solar panel is electrically connected to the input of the CN3130 IC for charge management. With few peripheral circuits, CN3130 IC can adaptively switch charging modes based on charging current and battery voltage. The rechargeable battery 502030 with a nominal voltage of 4 V is chosen to store the energy due to its mini type (30×20×5 mm^3), large discharge rate (0.5 C), and large nominal capacity (250 mAh). TPS63900 IC, an ultralow-static-power buck-boost converter is added as a front-end circuit to regulate the supply voltage for the EM4325 IC. The output voltages of the TPS63900 IC can be programmed by external resistors. Consequently, the supply voltage of the EM4325 IC and its output voltage can be tuned flexibly, enabling the green-smart window to realize full-space amplitude modulation.

The innovations of the controlling and powering system can be briefly summarized as: 1) a single-layered metal-substrate-metal structure (0.08×0.37λ^2) that minimizes the cost, weight, and visual obstruction; 2) full and compact integration of the IoT components, including the multispectral metasurface, microcontroller unit, operating circuit, wireless communication module, mobile application, and power supply; and 3) strategic integration of silicon solar cells to the RFID tag in a “face-to-face” configuration, achieving power supply, energy storage, and accessibility of IoT, while mitigating the visibility loss from opaque components.

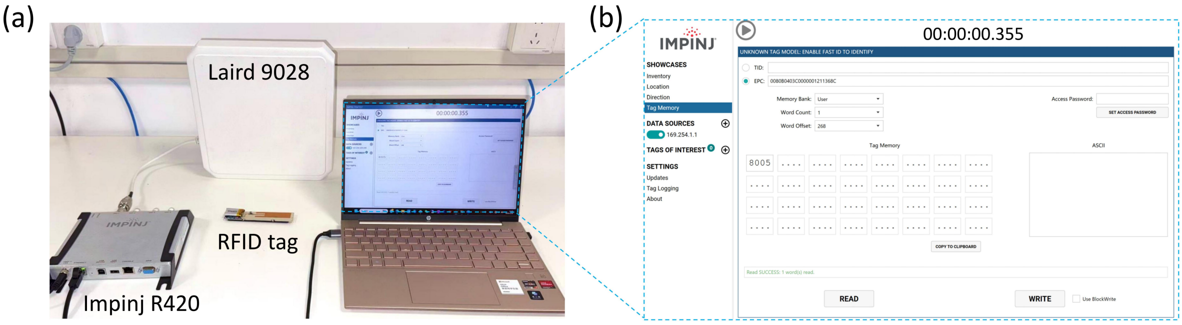

Fig. S8. (a) System configurations for remote control. (b) Software operator interface of ItemTest^{TM}.

Supplementary Note 7. Experimental Setup for the Designed RFID Tag

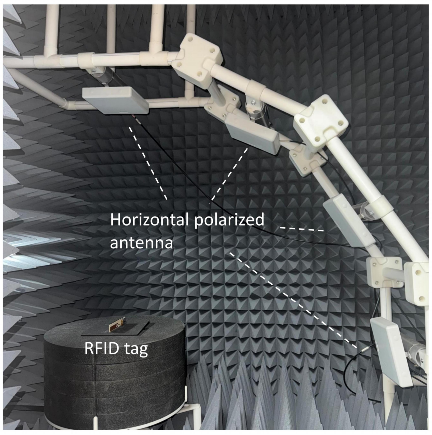

Fig. S9. Experimental setup to measure the read range and the radiation pattern of the designed RFID tag.

Supplementary Note 8. Effectiveness of the Transparent Absorber

As shown in **Fig. S10**a, the designed UHF transparent absorber has an ultra-thin thickness of 2 mm (0.006 λ_0, where λ_0 is the wavelength at the operational frequency of 915 MHz) and consists of a 2×3 array of elements. Each element is composed of a metal mesh strip with a sheet resistance of 0.2 Ω/sq on the top layer, a 2-mm-thick PC plate as the substrate, and a complete metal mesh ground with a sheet resistance of 0.1 Ω/sq on the bottom layer, as illustrated in **Fig. S10**b. In the full-wave simulation, the element is modelled with periodic boundary conditions. As depicted in **Fig. S10**c, the simulation results indicate that the element can achieve nearly perfect absorption at 915 MHz. Therefore, it can effectively absorb the spatial UHF waves to reduce the coupling between the green-smart window and the RFID tag.

To further validate this effect, we compare the RFID tag performance in four simulation scenarios, as shown in **Figs. S11**a,e,i,m. Regardless of the presence of the transparent absorber, the operational modes of the window, or the value of the window length (l_{window}), the S_{11} parameter of the tag remains below −10 dB at 915 MHz, demonstrating stable impedance characteristics, as depicted in **Figs. S11**b,f,j,n. The corresponding radiation patterns under the four scenarios are illustrated in **Figs. S11**c,g,k,o. Without the transparent absorber, the radiation pattern is significantly affected by the variation of the window length l_{window} (see **Figs. S11**g). In contrast, after introducing the absorber, the stability of the radiation pattern is

significantly improved (see **Figs. S11**c). The working principle can be revealed from the E-field distribution shown in **Figs. S11**d,h,l,p. The E-field intensity distribution on the transparent window is effectively mitigated by absorbing the surface and spatial waves, thus preventing undesirable distortion in the radiation characteristic of the RFID tag.

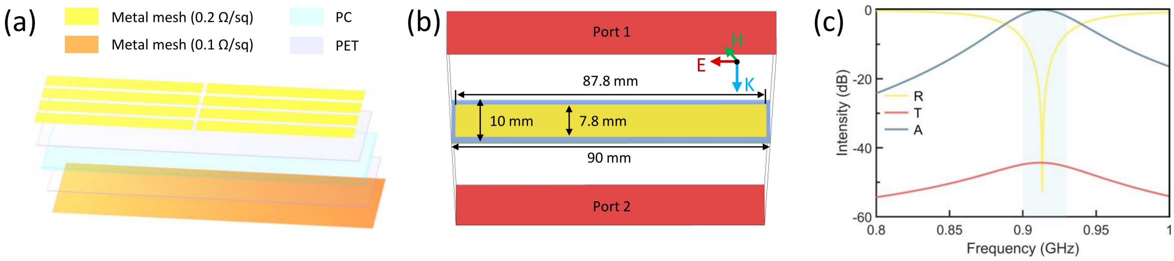

Fig. S10. (a) Structure of the designed transparent absorber. (b) Geometrical parameters of the element and the simulation boundary conditions. (c) Simulated transmission, reflection, and absorption spectra of the element.

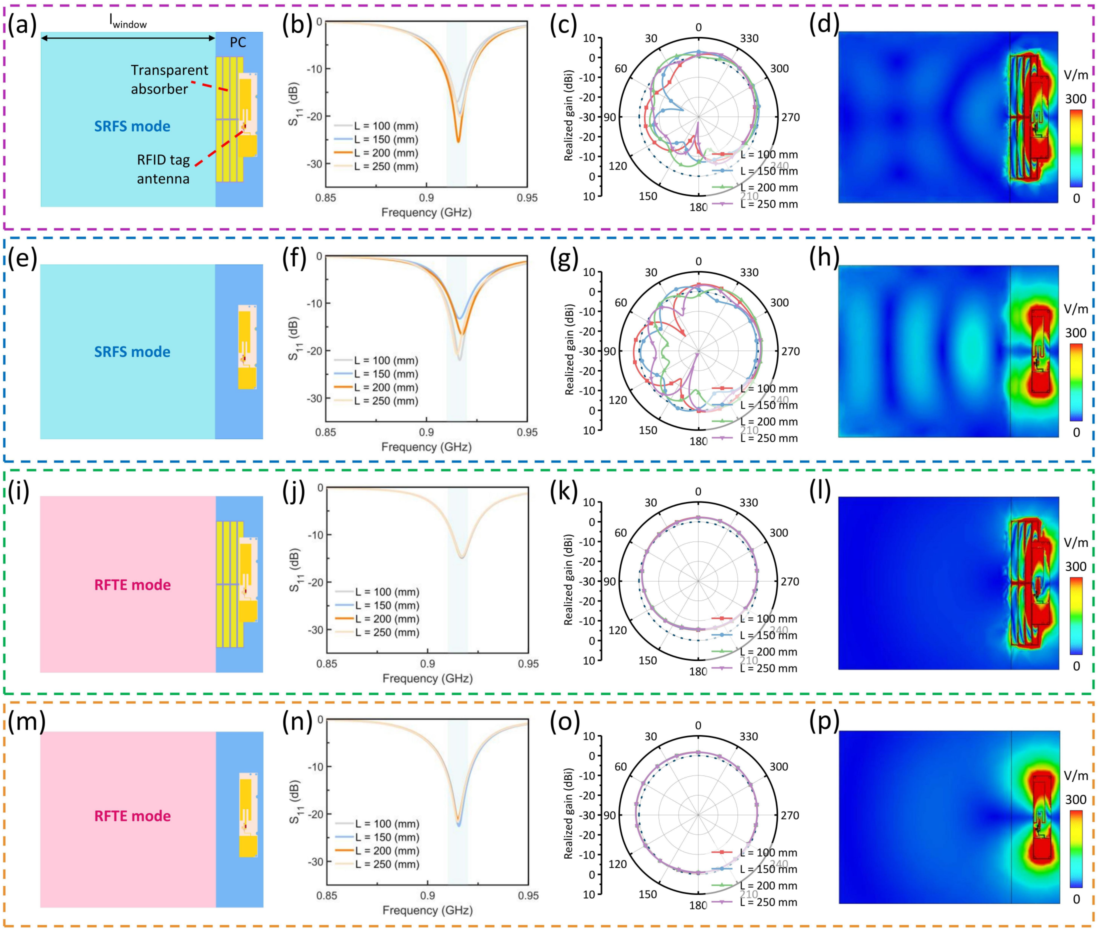

Fig. S11. (a,e,i,m) Four simulation scenarios in the full-wave simulation; (b,f,j,n) S_{11} parameters regarding l_{window}; (c,j,k,o) radiation patterns regarding l_{window}; and (d,h,l,p) E-field intensity distribution. The results

under the cases of (a-d) with the transparent absorber in the SRFS mode, (e-h) without the transparent absorber in the SRFS mode, (i-l) with the transparent absorber in the RFTE mode, and (m-p) without the transparent absorber in the RFTE mode.

Supplementary Note 9. Visible Transmittance of the Bare PC Plate and CTI Film

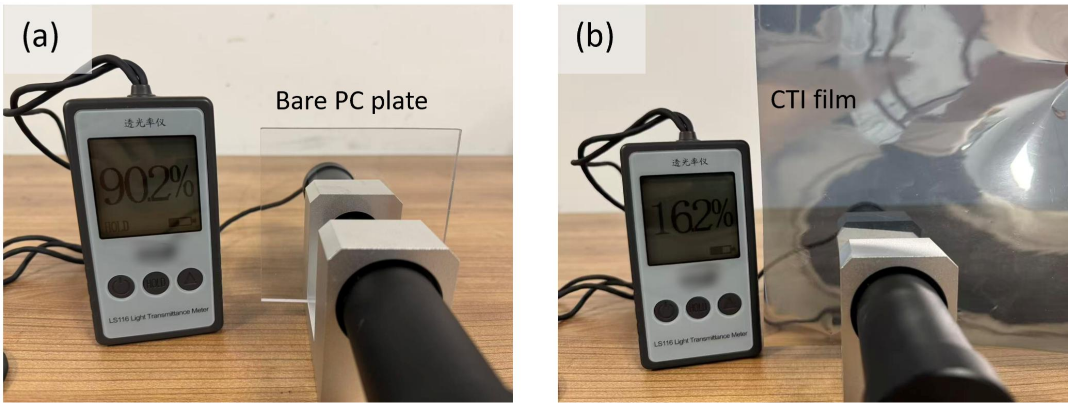

Fig. S12. The experimental setup to measure the average visible transmittance of (a) the bare PC plate and (b) the CTI film, which are measured as 90.2% and 16.2%, respectively.

Supplementary Note 10. Experimental Setups to Measure RF Transmissions

The experimental setups for measuring the RF performance are shown in **Fig. S13**. Two lens antennas are connected to the ports of the vector network analyzer (N5230C) by phase stable cables. By monitoring the transmitted and received continuous swept-frequency signals, the S-parameters of the DUTs can be measured. A pair of 3D-printed resin rotators is used to rotate the DUTs along the vertical plane with 10° angular resolution across full 360° positioning. The rotators have little effect on the measurement results since they are away from the focal spot of the lens antenna. The angular performance of the DUTs can be tested by recording the measured transmission spectra at each rotation angle.

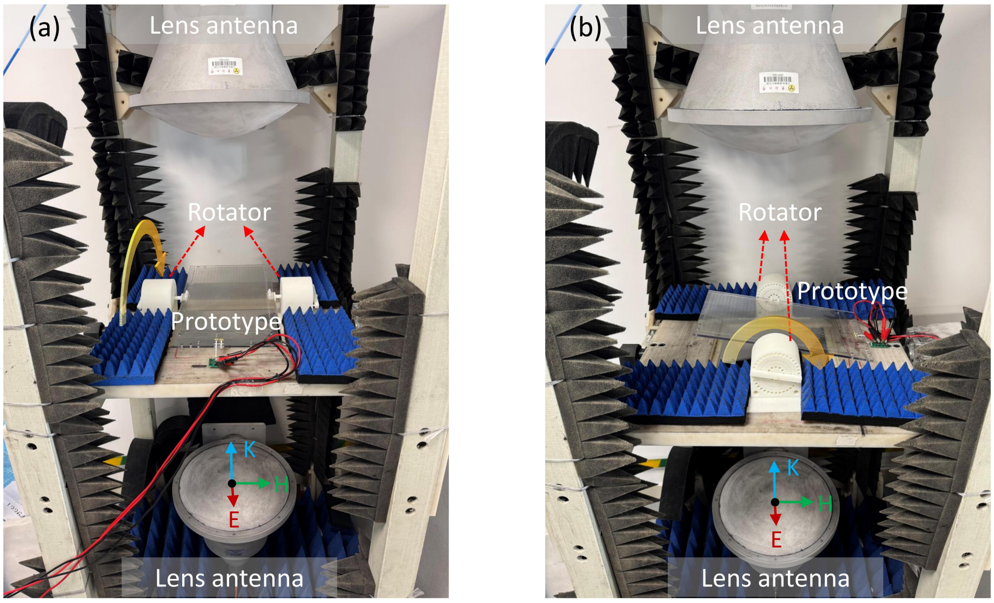

Fig. S13. Experimental setups to measure the angular performance of the DUTs under polarization modes of (a) TM and (b) TE, respectively.

Supplementary Note 11. Resonant Properties of the RFTE and SFRS Modes

Due to the frequency-dispersive nature of the metasurface, each functional mode of our design is supported by a specific resonant mode. For the SFRS mode, the simulated surface current distribution is shown in **Figs. S14**a,b. It is found that the current density exhibits significant non-uniformity across the meander-line resonators, with pronounced attenuation observed near the RF switches, which indicates the strong resonant property associated with the SRFS mode. The strong resonance may increase the parasitic loss in the prototype, lowering the Q factor of the LC resonator and thus reducing the measured peak resonance value.

The simulated surface current distribution of the meta-atom operating under the RFTE mode is displayed in **Figs. S14**c,d. The results illustrate a smooth and continuous current distribution without abrupt transitions, revealing the characteristics of the weak resonance of the RFTE mode. This property mitigates the adverse effects of loss factors on the prototype, ensuring its excellent performance in the RFTE mode during measurements.

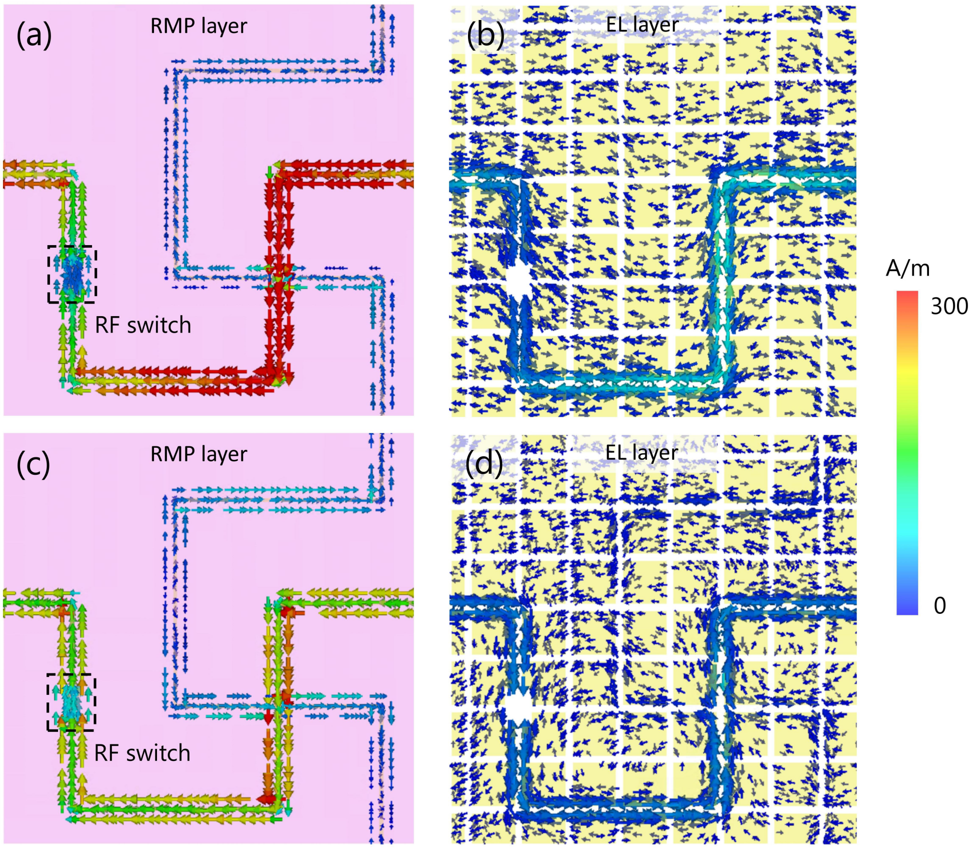

Fig. S14. (a,b) The simulated surface current distribution of the RMP and EL layer in the SRFS mode, respectively. (c,d) The simulated surface current distribution of the RMP and EL layer in the RFTE mode, respectively.

Supplementary Note 12. Transmission Enhancement in the RFTE Mode

To quantify the transmission enhancement performance, we compare the transmission amplitudes of the metasurface-based window in the RFTE mode with a bare PC plate (REF #1) and a PC plate coated with EL layers on both sides (REF #3). The thicknesses of the REF #1 and #3 are the same as the metasurface-based window. The simulation and measurement results of the two REFs are shown in **Figs. S15**a-d and e-h, respectively. The transmission enhancement (EN), defined by EN = $20\log_{10}(t_{RFTE}^{window}/t^{sample})$ (dB), is quantitatively demonstrated in **Figs. S15**i-p, respectively. The results reveal that the designed metasurface-based window in the RFTE mode can pronouncedly enhance the TE-polarized transmission amplitudes compared with both REFs. In addition, the TM-polarized transmission amplitudes of the prototype, REF #1, and RFE #3 remain nearly identical, suggesting that the Brewster effect of

the PC material (isotropic natural materials) at oblique incidence in TM polarization is well preserved.

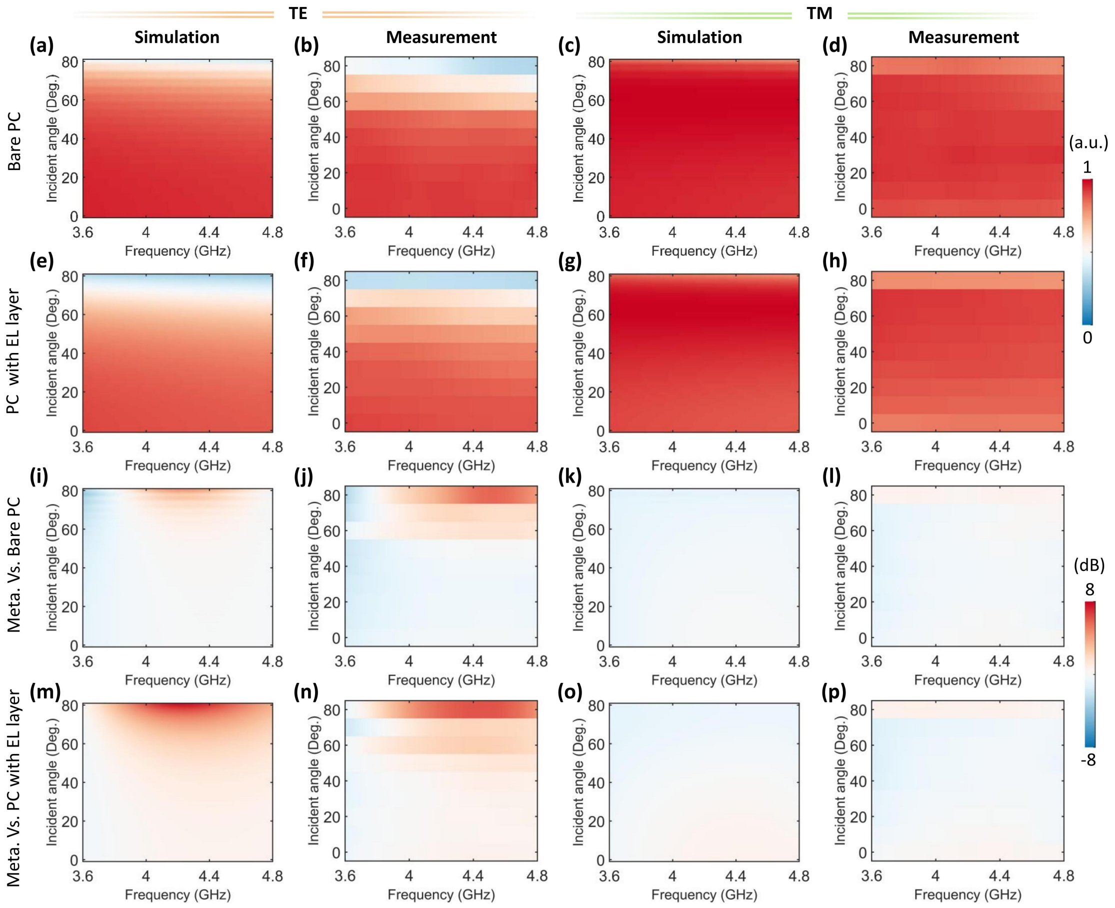

Fig. S15. (a-h) Simulated and measured transmission amplitude spectra of RFE #1 and #3. (i-p) The simulated and measured transmission enhancement of the metasurface-based window. The transmission enhancement is defined as EN = $20\log_{10}(t_{RFTE}^{prototype}/t^{REF})$.

Supplementary Note 13. Broadband Characteristics Across 1-8 GHz

The simulated transmission amplitude spectra of the designed green-smart window over the 1-8 GHz range are shown in **Fig. S16**. For the SRFS mode, the -10 dB transmission bandwidth is 3.8-4.7 GHz, with the relative bandwidth of 21%. In the frequency ranges of 1.1-3.1 GHz and 5-8 GHz, the transmission amplitudes are higher than -5 dB, ensuring good transmission performance out of the band. During the measurement, a pair of Vivaldi antennas is used to transmit and receive the continuous waves to measure the $|S_{21}|$ curves of the prototype. The

measured and simulated results are in good agreement, verifying that the prototype only shields the EM waves in a narrow band rather than the whole band.

It is worth mentioning that the upper envelopes in **Fig. S16** are higher than -5 dB across the entire 1.1-8 GHz frequency band, where it involves 5G primary communication frequency bands of n1(1.92-2.17 GHz), n41 (2.515-2.675 GHz), n77(3.3-4.2 GHz), n78(3.3-3.8 GHz), and n79(4.4-5 GHz), and commercial wireless bands of Wi-Fi (2.4-2.48 GHz and 5.15-5.85 GHz) and Bluetooth (2.4-2.48 GHz). This indicates that the metasurface can maintain good O2I links in an ultra-wide band to support diverse communication scenarios through mode switching. The previously proposed passive-metasurface-enabled glass can only enhance the RF transmission in a limited operating band, while severely attenuating the transmission out of band[8,9,13,15]. This work overcomes the inherent bandwidth limitation of conventional designs by using the reconfigurable metasurface.

Additionally, the meta-atom actually presents dual-band RF responses. In the upper frequency band (3.6-4.8 GHz), the ‘00’ and ‘11’ coding states function as narrow stopband and wide passband spatial filters, respectively, as completely demonstrated in Fig. 3 in the main text. Conversely, in the lower frequency band (1.2-2.4 GHz), the ‘00’ and ‘11’ coding states function as wide passband and narrow stopband spatial filters, respectively, as shown in **Fig. S16**. The angular performance in the lower frequency band is thoroughly inspected using the full-wave simulation. As illustrated in **Fig. S17**, the results indicate that the wide-angle high RF transmission (‘00’ coding state) is achieved near 2 GHz, and the selective RF shielding mode (‘11’ coding state) operates around 1.6 GHz with good angular stability. The dual-band capability of the designed metasurface allows flexible manipulation of both dual-band and broadband RF signals, demonstrating good adaptability to various communication bands.

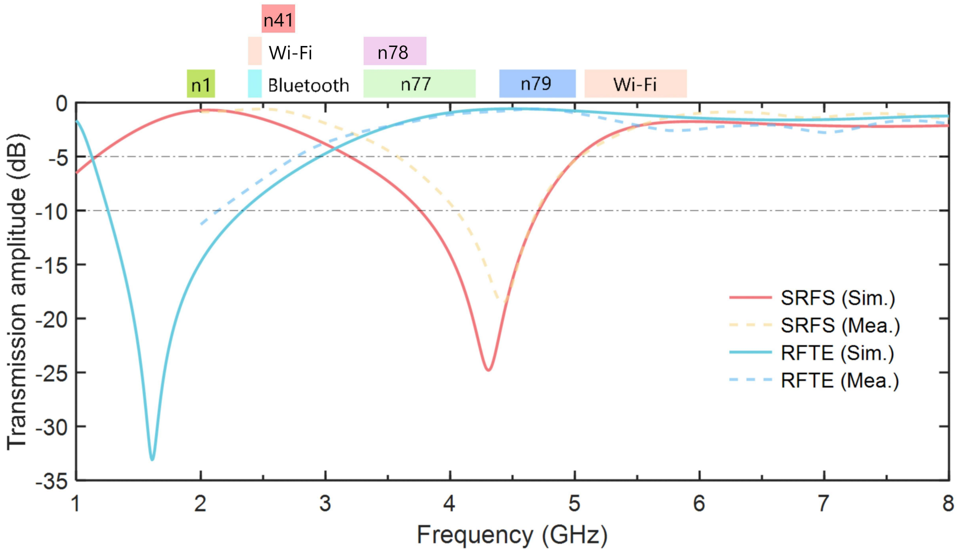

Fig. S16. The simulated and measured transmission amplitude spectra of the designed metasurface over a 1-8 GHz frequency band.

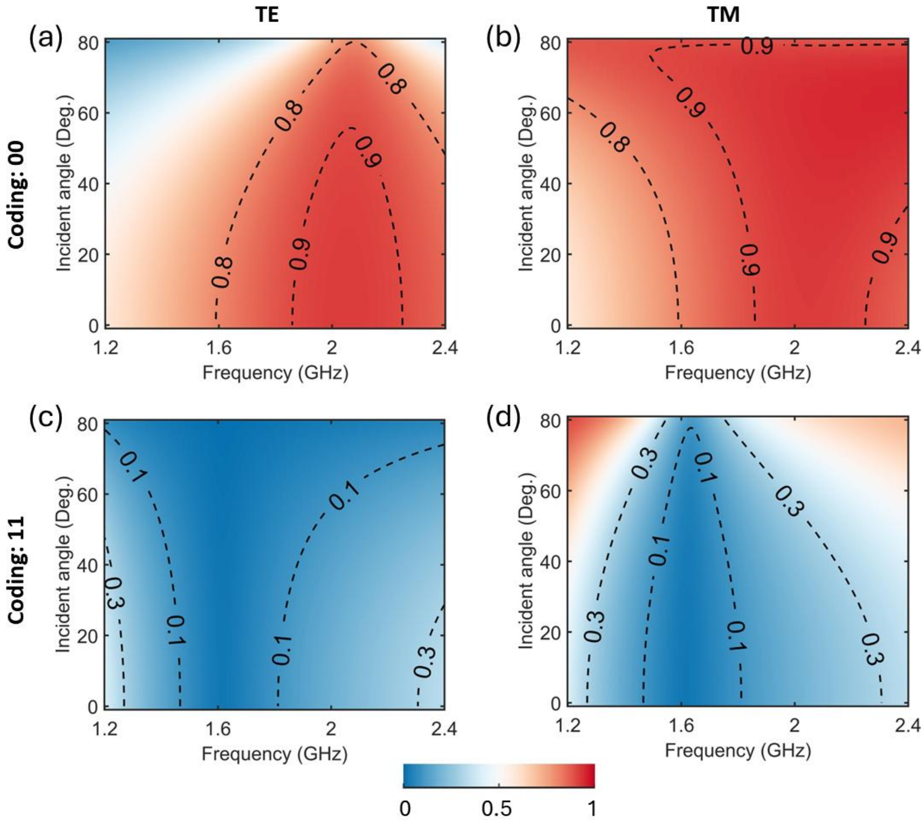

Fig. S17. The simulated transmission spectra of the green-smart window in the angle range from 0° to 80°. (a,b) The results in ‘00’ coding state under TE and TM polarizations, respectively. (c,d) The results in ‘11’ coding state under TE and TM polarizations, respectively.

Supplementary References

1 Horodynski, M., Kühmayer, M., Ferise, C., Rotter, S. & Davy, M. Anti-reflection structure for perfect transmission through complex media. *Nature* **607**, 281-286 (2022).

2 Luo, H. *et al.* Dielectric metamaterials with effective self-duality and full-polarization omnidirectional brewster effect. *Light-Sci. Appl.* **13**, 262 (2024).

3 Im, K., Kang, J.-H. & Park, Q. H. Universal impedance matching and the perfect transmission of white light. *Nat. Photonics* **12**, 143-149 (2018).

4 Du, C. *et al.* Radiofrequency Transparent Uniaxial Dual‐Polarized Metasurface with Ultrawide Brewster Angle Stability. *Laser Photon. Rev.* **19**, 2500190 (2025).

5 Yang, Y., Li, W., Salama, K. N. & Shamim, A. Polarization Insensitive and Transparent Frequency Selective Surface for Dual Band GSM Shielding. *IEEE Trans. Antennas Propag.* **69**, 2779-2789 (2021).

6 Farooq, U., Shafique, M. F. & Mughal, M. J. Polarization Insensitive Dual Band Frequency Selective Surface for RF Shielding Through Glass Windows. *IEEE Trans. Electromagn. Compat.* **62**, 93-100 (2020).

7 Darvish, A. & Kishk, A. A. X-Band Nearfield Shielding Metasurface With Adjustable Reflection/Transmission Zeroes. *IEEE Trans. Electromagn. Compat.* **64**, 1602-1613 (2022).

8 Jiang, R. Z. et al. Optically Transparent Metasurface with High RF Transmittance and Wide-Angle Stability for Dual Bands and Dual Polarizations. *Adv. Opt. Mater.* **11**, 2300553 (2023).

9 Jiang, R. Z. et al. A Single-Layered Wideband and Wide-Angle Transparent Metasurface for Enhancing the EM-Wave Transmissions Through Glass. *IEEE Trans. Antennas Propag.* **71**, 6593-6605 (2023).

10 MUNK & Ben, A. *Frequency Selective Surfaces: Theory and Design*. (Frequency Selective Surfaces: Theory and Design, 2005).

11 Zheng, J., Zheng, H., Pang, Y., Qu, B. & Xu, Z. A Metasurface Glass for Energy Saving and 5G Mobile Communication Signal Enhancement. *Small* **21**, 2408598 (2024).

12 Zheng, J., Zheng, H., Pang, Y., Qu, B. & Xu, Z. Metasurface Glass for Wireless Communication and Energy Saving. *Small* **20**, 2309050 (2024).

13 Safari, M., Kherani, N. P. & Eleftheriades, G. V. Multi-Functional Metasurface: Visibly and RF Transparent, NIR Control and Low Thermal Emissivity. *Adv. Opt. Mater.* **9**, 2100176 (2021).

14 Zhang, W. J., Wang, T. M., Zhong, L. Z., Wu, X. W. & Cui, M. Theoretical study of infrared emissivity of indium tin oxide films. *Acta Phys. Sin.* **54**, 4439-4444 (2005).

15 Safari, M., He, Y., Kim, M., Kherani, N. P. & Eleftheriades, G. V. Optically and radio frequency (RF) transparent meta-glass. *Nanophotonics* **9**, 3889-3898 (2020).
