# Supplementary material for: A metasurface-enabled green-smart window for intelligent wireless communications with high visible transparency and low infrared emissivity
Source: Nat Commun. 2026 May 13;17:6414. doi: 10.1038/s41467-026-72643-x (PMC13376189; doi:10.1038/s41467-026-72643-x)
Supplement: Supplementary file 2 — Transparent Peer Review file [file 41467_2026_72643_MOESM2_ESM.pdf]

nature portfolio

Peer Review File

A metasurface-enabled green-smart window for intelligent wireless communications with high visible transparency and low infrared emissivity

Corresponding Author: Professor Tie Jun Cui

This file contains all reviewer reports in order by version, followed by all author rebuttals in order by version.

Version 0:

Reviewer comments:

Reviewer #1

(Remarks to the Author)
The manuscript from Jiang and coworkers reports a green-smart window based on programmable metasurface with several superiority including high visible transparency,low infrared emissivity, and reconfigurable electromagnetic responses in a wide angular range and dual polarizations. The potential applications of such smart window are demonstrated by several models including remote-sensing range, self-powering capability, and thermal management. The manuscript is well-written and all the conclusions can be supported by the experimental results. I think this manuscript can be accepted after some minor revisions.
(1) The abbreviation should be unified. For example, there is no explain about the RFID in abstract
(2) The transparency of the final window is not high according to the real image and spectra data
(3) The low IR emissivity is not clear, the author is recommend to define the range of IR light and provide data in main manuscript.

Reviewer #2

(Remarks to the Author)
This manuscript presents excellent work on integrating dynamic metasurfaces into a green-smart window platform, achieving high visible transparency, low infrared emissivity, and dynamic functionality for wireless communication. The physics and logic are clearly explained, and the results represent a significant breakthrough with strong implications for sustainable and intelligent building technologies. Overall, I find this manuscript to be of exceptionally high quality. I only have a few minor comments for the authors, which are listed below.

1. The discussion of all experimental results shown in Fig. 3 is incomplete. The authors should ensure that every panel and dataset is thoroughly addressed in the manuscript. In addition, the noticeably lower intensity observed in the measured results compared to the simulations should be briefly discussed, with possible reasons such as fabrication imperfections or measurement limitations.
2. The description of the design concept and resonant properties of the meta-atom is not clear. The presence of multiple horizontal and vertical metallic lines in the structure suggests some polarization-selective mechanism, but this is not explained in detail. The authors should clarify whether this geometry is specifically intended to realize polarization-selective responses or if it serves another design purpose.
3. The labels in Fig. 5c are unclear and difficult to read. All figure labels and annotations should be carefully revised to ensure clarity and readability.
4. The manuscript does not mention the switching speed of the developed metasurface, which is a critical parameter for evaluating its potential in practical applications. The authors should provide at least an estimate or discussion of the switching time, supported by experimental data if available.
5. For clarity, the authors should provide an enlarged image of the fabricated metasurface so that the structural details of the unit cells can be clearly seen.

Reviewer #3

(Remarks to the Author)
Please refer to the attachment.

Version 1:

Reviewer comments:

Reviewer #2

(Remarks to the Author)
The authors have carefully addressed all of the comments and suggestions raised in the previous review round. The revisions are thorough, and the responses clearly explain how each issue was resolved. The manuscript is now technically sound, well organized, and the presentation has been significantly improved. I have no further concerns, and I am satisfied that the current version fully meets the standards of the journal. I therefore recommend the manuscript for acceptance in its present form.

Reviewer #3

(Remarks to the Author)
In the revised manuscript, the concerns have been addressed to a good extent. Concrete data is supplemented. The comparison tables and related discussion are sound.

Open Access This Peer Review File is licensed under a Creative Commons Attribution 4.0 International License, which permits use, sharing, adaptation, distribution and reproduction in any medium or format, as long as you give appropriate credit to the original author(s) and the source, provide a link to the Creative Commons license, and indicate if changes were made.
In cases where reviewers are anonymous, credit should be given to 'Anonymous Referee' and the source.
The images or other third party material in this Peer Review File are included in the article's Creative Commons license, unless indicated otherwise in a credit line to the material. If material is not included in the article's Creative Commons license and your intended use is not permitted by statutory regulation or exceeds the permitted use, you will need to obtain permission directly from the copyright holder.
To view a copy of this license, visit https://creativecommons.org/licenses/by/4.0/

This study presents a green-smart metasurface window enabling RF-IR-visible multi-band control. Its core functionalities include high visible-light transparency, low thermal emissivity, and reconfigurable RF responses tailored for wireless communications. Specifically, the RF responses can be switched between three distinct modes via embedded RF switches: wide-angle-enhanced transmission, frequency-selective shielded transmission, and polarization filtering. Notably, the reconfiguration of these modes is remotely achieved using RFID technology, with power supplied by an integrated solar panel for self-sustainability.

Green smart windows are undoubtedly key components in the future of information-driven living, and a multi-band integrated design is therefore highly imperative. While the wireless communication experiments, thermal insulation tests, and other characterizations in this work do confirm that the proposed window outperforms pure PC counterparts and some reference samples, I think the RF shielding effect is still not good enough. The reasons are stated below with other issues.

1. In the RFTE mode, the transmission is enhanced as compare to pure PC. The performance at large incident angle is still very good. The main issue is in the SRFS mode. Besides of shielding a particular frequency (around 4.3 GHz), it also introduces large loss to other frequencies, at least more than 5 dB from Fig. 2e and Fig.3. It is more like shielding over the whole band, not a narrow band. The shielding effect deteriorates with the incident angle.
2. In the wireless communication experiment, the I2I communication can effectively isolate the co-channel interference from O2I. But does it help to maintain strong O2I links outside the frequency band at the same time? Especially when the window introduces more insertion loss in the whole band in the SRFS mode, there is no test to verify this conclusion.
3. The window integrates the RFID and solar panel for self-powering. This part is more like an engineering issue. However, it is not clearly explained whether this is a direct superposition of existing technologies or involves technological innovation.
4. Does the design compromise performance in the visible and IR bands to facilitate RF modulation? The visible transparency is 53.6% (average) and 62% (maximum). How is it compared to the PC? The IR emissivity is 0.35, which is not low enough. From the cavity temperature experiment, it is only a little better than pure PC, and still far from the thermal isolation film.
5. From the supplementary details, the EL layer and RMP layer are separately designed from the circuit model. Is there any coupling between them?
6. Minor comments

(1) In Fig. 2g, the area is not large enough above and below the window to observe the field distribution. From the colorbar, what is the reason that the maximum values in TE and TM modes are very different?

(2) In the reference, there are a lot of literature information is missing.

(3) Fig. 4(d) the unit should be changed from GHz to MHz.

(4) In Fig. 7b, the PC+EL already shows good improvement in communication enhancement in oblique incidence. What is the reason? And how to accurately account for the contribution of the metasurface?

(5) The REF number in the figure caption, main text and inside the figure are not consistent in Fig. 7b,e.

RE: Manuscript #NCOMMS-25-58103-T "A metasurface-enabled green-smart window for intelligent wireless communications with high visible transparency and low infrared emissivity," by Rui Zhe Jiang, *et* al.

Responses to Reviewers' Comments

We sincerely appreciate the constructive comments and suggestions from all reviewers, which help us improve the quality of the manuscript significantly and make the manuscript more complete. Below, we provide our detailed responses (in blue font) to each comment (in regular font). We have revised the manuscript and supplementary information accordingly and highlighted all changes (in yellow background) in the revised documents. We hope that the response letter has clarified all aspects concerned by the reviewers.

To Reviewer #1:

Comment:

The manuscript from Jiang and coworkers reports a green-smart window based on programmable metasurface with several superiority including high visible transparency,low infrared emissivity, and reconfigurable electromagnetic responses in a wide angular range and dual polarizations. The potential applications of such smart window are demonstrated by several models including remote-sensing range, self-powering capability, and thermal management. The manuscript is well-written and all the conclusions can be supported by the experimental results. I think this manuscript can be accepted after some minor revisions.

Our Response:

We are deeply grateful for your encouraging feedback, which is greatly appreciated and has been a significant source of motivation. All raised points have been addressed, and the manuscript has been revised accordingly.

Comment 1:

The abbreviation should be unified. For example, there is no explain about the RFID in abstract.

Our Response:

Thank you very much for your valuable suggestion. We acknowledge the reviewer's comment regarding the definition of RFID. The full terminology “radio frequency identification (RFID)” has been explicitly defined at its first mention in the revised version. In addition, we have also carefully reviewed the entire manuscript to ensure that no similar abbreviation omissions remain.

Our Action:

We have added the full terminology of the RFID in the **abstract**, also given as follows:

“By integrating the window with a compact RFID (radio frequency identification) tag…”

Comment 2:

The transparency of the final window is not high according to the real image and spectra data.

Our Response:

Thank you very much for your valuable comment. We fully understand the reviewer’s concern regarding the overall visible transparency. Actually, the transparency of our window is good and completely acceptable for a building window. As shown in Fig. R1 (see Fig. 5a in the manuscript), the building behind the prototype can be clearly seen, verifying the superior visible transparency. The measured visible transparency of the fabricated prototype is 53.6% (average) and 62% (maximum). According to the Industry Standard Documents (Technical Specification for Application of Architectural Glass, JGJ 113-2015), the visible transparency of the low-emissivity (low-E) glass should be higher than 40%. Therefore, the transparency of our design fully meets the industry standard for architectural glass.

In addition, we also compare the prototype with other previously reported reconfigurable transparent metasurfaces, as listed in Table R1. References [1] and [2] have made good efforts in achieving high visible transparency through special materials and processing technologies, but both the sapphire substrate and micrometer-scale VO2 adopted in their work are extremely costly and difficult to mass-produce. The meticulously engineered ultrathin meander-line structure enables high average visible transparency, even when employing commonly available commercial materials, such as PC plates and PET films, and standard flexible printed circuit fabrication processes. This results in superior visible transparency compared to other works [3] and [4] utilizing similar processing technologies.

Though the visible transparency of the current-stage prototype can fully meet the industry requirements, to further address the reviewer's concern, we propose an alternative strategy employing a pre-installation process to enhance the optical clarity. The fabrication process is illustrated in Fig. R2, which mainly consists of the following key steps: 1) ITO deposition using magnetron sputtering technology. 2) Patterning ITO patches using laser etching technology. 3) Forming metallic structures via an electric field-oriented micro-jet printing technique[5]. 4) Mounting RF switches using the reflow soldering process (the glass transition temperature is higher than 400 °C). Thus, visible losses from the PET films and adhesive layer are eliminated by this fabrication process, resulting in a visible-light transmittance of at least 60%.

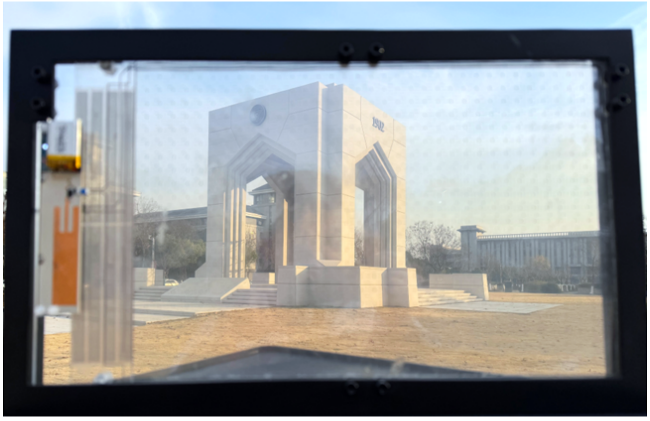

Fig. R1. The photograph of the proposed prototype.

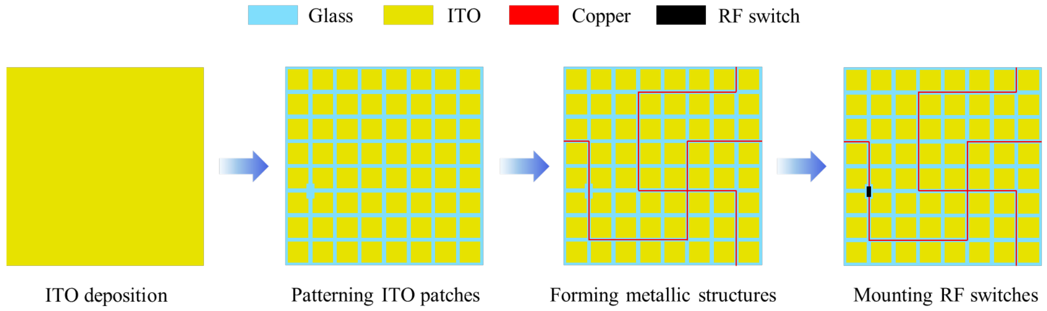

Fig. R2. Fabrication process of the pre-installation method to improve the visible transparency of the green-smart window.

Table R1. Comprehensive comparison between this work and other works on reconfigurable transparent metasurfaces

Refer-ence	Substrate	Processing technology	Active component	Trans-parency	Thermal management	Cost	Layer number

[1]	Sapphire H_s = 2 mm	Laser etching and lithography	Micrometer-sacle VO_2	80%	No	High	3
[2]	Sapphire H_s = 1.6 mm	Laser etching and lithography	Micrometer-sacle VO_2	88%	No	High	3
[3]	PC H_s = 3 mm	Flexible printed circuit	rheostatic diodes	52%	No	Medium	3
[4]	PC H_s = 2.5 mm	Flexible printed circuit	Varactor	49.3%	No	Medium	2
This work	PC H_s = 5 mm	Flexible printed circuit	Low-power RF switch	53.6%	Yes	Medium	4

References:

1 Xia, C., Lu, Z., Zhang, Y. & Tan, J. Broadband High Optical Transparent Intelligent Metasurface for Adaptive Electromagnetic Wave Manipulation. Research 7, 0334 (2024).

2 Lu, Z., Xia, C., Zhang, Y. & Tan, J. Metasurface with Electrically Tunable Microwave Transmission Amplitude and Broadband High Optical Transparency. ACS Appl. Mater. Interfaces 15, 29440-29448 (2023).

3 Cai, X. et al. Metasurface Window With Customized Transparency for Sunlight and Switchable Transmission for Microwaves. IEEE Trans. Microw. Theory Tech. 7, 3670-3682 (2024).

4 Liang, J. C. et al. An Optically Transparent Reconfigurable Intelligent Surface with Low Angular Sensitivity. Adv. Opt. Mater. 12, 2202081 (2024).

5 Zhang, H. et al. Electric field oriented deposition manufacturing of low loss, high gain flexible transparent antenna utilizing the skin effect. Int. J. Extreme Manuf. 73, 055103 (2025).

Comment 3:

The low IR emissivity is not clear, the author is recommend to define the range of IR light and provide data in main manuscript.

Our Response:

Thank you very much for your valuable suggestion. The reflection r, transmittance t, and the emissivity ε over the 2.5-25 μm wavelength range have been measured to verify the low-E property, as shown in Fig. R3. The average IR emissivity in the whole band (2.5-25 μm) is calculated as 0.34. Given that the emissivity within the atmospheric windows of 3-5 μm and 8-14 μm is more significant in the IR detection and indoor heating, we also calculate the average emissivity in the two bands, which are $\varepsilon_{3\text{-}5um}$

= 0.35 and $\varepsilon_{8\text{-}14um}$ = 0.33, respectively. Benefit from the low-E property, the prototype can regulate heat transfer, thus saving energy consumption from the building's temperature control system.

In detail, the IR spectra can be divided into near-infrared range (NIR, 0.75-2.5 μm), long-wavelength infrared range (LWIR, 2.5-25 μm), and far-infrared range (FIR, 25-1000 μm). The emissivity of our prototype is low in the LWIR and FIR ranges, but may increase to some extent in the NIR band due to the intrinsic IR property of the Indium Tin Oxide (ITO). Specifically, the epsilon near zero point of the ITO occurs in the NIR range (around 1.8 μm), where it exhibits dielectric behavior at shorter wavelengths and metallic conductor properties at longer wavelengths. Owing to its low cost, high visible transparency, and low emissivity in the LWIR range, ITO remains the preferred and mainstream material for applications in infrared stealth and low-emissivity (low-E) glass.

Our Action:

The IR performance across the entire LWIR range, previously shown in Fig. 6c, has been replotted as Fig. R3 in the revised manuscript for clarity. We have added the description of the wavelength range in the measurement and the average IR emissivity in the whole LWIR band in the second paragraph of the "Thermal Regulation" Section in the revised manuscript.

"To experimentally verify the low IR emissivity, we measured reflection, transmittance, and emissivity of the prototype in the long-wavelength infrared radiation range (2.5-25 μm)……"

"The average IR emissivity across the LWIR range is calculated as 0.34."

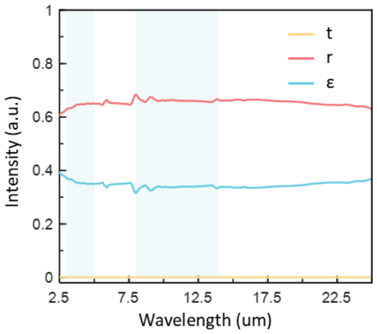

Fig. R3. The measured IR transmittance *t*, reflectance *r*, and emissivity *ε* from 2.5 to 25 μm.

To Reviewer #2:

Comment:

This manuscript presents excellent work on integrating dynamic metasurfaces into a green-smart window platform, achieving high visible transparency, low infrared emissivity, and dynamic functionality for wireless communication. The physics and logic are clearly explained, and the results represent a significant breakthrough with strong implications for sustainable and intelligent building technologies. Overall, I find this manuscript to be of exceptionally high quality. I only have a few minor comments for the authors, which are listed below.

Our Response:

Thank you very much for your recognition of our work. We are also impressed by your professional and enlightening suggestions, which help us improve the manuscript's quality significantly. We have carefully revised the manuscript according to your suggestions.

Comment 1:

The discussion of all experimental results shown in Fig. 3 is incomplete. The authors should ensure that every panel and dataset is thoroughly addressed in the manuscript. In addition, the noticeably lower intensity observed in the measured results compared to the simulations should be briefly discussed, with possible reasons such as fabrication imperfections or measurement limitations.

Our Response:

Thank you very much for your good comment and valuable suggestion. As suggested by the reviewer, we have added detailed discussions on every panel and dataset in Fig. 3 (in the manuscript) to improve the clarity of the manuscript. Additionally, we have analyzed the difference between measured and simulated results, which has been succinctly summarized in the revised manuscript due to space limitations. Here, we demonstrate a detailed analysis of the difference between the simulation and the measurement, which can be mainly attributed to the following reasons:

1) ***Parasitic effect of the active components***

According to the reported work, for the passive metasurface, the measured and simulated reflection and transmission efficiencies are in high agreement[7-9]. However, for most of the reconfigurable metasurfaces with the active components (PIN diode, varactor diode, etc.), the measured amplitudes at the resonant frequencies decreased at least 1 dB (nearly 2 dB on average) compared to the simulated results[10-12].

Fortunately, the RFTE mode operates in a weak resonance so that the efficiency loss is slight, leading to sufficiently high transmission amplitudes to realize transmission enhancement.

2) ***Error in the fabrication and assembly process***

In our design, the prototype is fabricated by a film application solution, in which a PC substrate is sandwiched by two composite film coatings, each containing an EL and RMP layer. The fabrication tolerance of the metallic pattern on the EL and RMP layers is within 10%, as shown in Fig. 5c in the manuscript. The RMP and EL layers have been carefully attached to both sides of the PC plate with little air bubbles and good alignment. As a prospect, here, we develop a pre-installation method for large-scale deployment to further minimize the assembly errors, as shown in Fig. R2.

3) ***Measurement limitations***

For passive band-stop frequency selective surfaces (FSSs), a 5–15 dB discrepancy between the measured and simulated resonance peak magnitudes is routinely reported in the previous literature[13-16]. This divergence suggests that excessively sharp band-stop resonance peaks are challenging to measure accurately due to measurement limitations.

Our Action:

We have added the analysis of all the panels and the dataset in Fig.3 (in the manuscript), and supplemented the discussions on the error analysis in the first paragraph of **“RF performance” Section** in the revised manuscript.

“For TE polarization, the measured results under coding sequences of ‘11’, ‘00’, ‘01’, and ‘10’ are displayed in Figs. 3a,f,j, and n, respectively; the corresponding results for TM polarization are presented in Figs. 3d,h,l, and p, respectively. The comparison between the simulated and measured results reveals a minor discrepancy, characterized by a blue shift of 0.3 GHz and slight transmission amplitude differences, as shown in Figs. 2 and 3. The average error between simulation and measurement is quantified as $\mathrm{Er} = \frac{1}{18}\sum_{\theta\in\{0,10,\dots,80\}} t^{te}_{mea}(\theta) + t^{tm}_{mea}(\theta) - t^{te}_{sim}(\theta) - t^{tm}_{sim}(\theta)$ (dB). The calculated average errors in the RFTE and SRFS modes are -0.42 dB and 6.8 dB, respectively. The error discrepancy can be explained by different resonant properties of the RFTE and SRFS modes (see Supplementary Note 11 for details). The non-ideal measured results can be attributed to the measurement

limitations and imperfections during fabrication and assembly, including coating preparation, component soldering, and lamination of multilayer structures."

Comment 2:

The description of the design concept and resonant properties of the meta-atom is not clear. The presence of multiple horizontal and vertical metallic lines in the structure suggests some polarization-selective mechanism, but this is not explained in detail. The authors should clarify whether this geometry is specifically intended to realize polarization-selective responses or if it serves another design purpose.

Our Response:

Thank you very much for your valuable comments. The discussions on the design concept, resonant properties, and reasons for the polarization-selective responses of the proposed meta-atom are shown below in detail.

1) Design concept

In the Supplementary Note 1, we have clarified the underlying mechanism to realize RFTE and SFRS, wherein the equivalent reactance of the metasurface pattern X_f must align with theoretical expectations (i.e., $X_f = X_{expect}$). Given that meta-atom structures that satisfy the requirement at a specific frequency are not unique, we prioritize the selection of structures with high angular and frequency stability among multiple candidates for practical values. To interpret this point, two candidate structures (structures #A and #B) are developed, as shown in Fig. R4a. For analytical convenience, EL layers are not incorporated into either structure #A or #B. As illustrated in Figs. R4b,c, the central operating frequency of structure #A shifts from 4.3 GHz to 3.95 GHz within a 0°-80° angular range, while that of structure #B only shifts from 4.3 GHz to 4.2 GHz.

In addition, for structure #A, a sharp transmission drop occurs near 4.25 GHz when illuminated by the oblique TM-polarized waves due to the trapped mode (a specific high-order resonance that converts most incident wave energy into surface waves), thereby inducing an abrupt transmission drop within the operating band. Therefore, though both structures achieve high RF transmission at 4.3 GHz, structure #B reveals a superior angular sensitivity and wideband stability compared with structure #A. In our prior research, we found that meta-atoms smaller than $\lambda_0/5$ (where λ_0 denotes wavelength in the air) have good

angular stability, and the onset frequency of the trapped mode can be lifted out of the operating band[8]. To achieve this compact scale, the straight-line structure was compressed into meandered configurations, thereby reducing the meta-atom periodicity.

2) Resonant properties

Due to the frequency-dispersive nature of the metasurface, each functional mode of our design is supported by a specific resonant mode. For the SFRS mode, the simulated surface current distribution is shown in Figs. R5a,b. It is found that the current density exhibits significant non-uniformity across the meander-line resonators, with pronounced attenuation observed near the RF switches, which indicates the strong resonant mechanism associated with the SRFS mode.

The simulated surface current distribution of the meta-atom operating under the RFTE mode is displayed in Figs. R5c,d. The results illustrate a smooth and continuous current distribution without abrupt transitions, revealing the characteristics of the weak resonant mechanism of the RFTE mode.

3) Polarization-selective responses

In this study, dynamical EM wave manipulation is highlighted. It is noted that an active component (e.g., PIN diode, varactor, photodiode…) can only modulate a single polarized wave and remains ineffective for the orthogonal polarization. To enable full control of dual polarizations, a minimum of two tunable elements per meta-atom is required. Therefore, two orthogonally oriented RF switches are loaded onto the top and bottom of the RMP layers to manipulate TE- and TM-polarized waves. The biasing lines (e.g., vertical metallic lines) are seamlessly integrated into the metasurface resonators (e.g., vertical metallic lines). By applying the voltage on the four biasing buses, four coding states of '00', '01', '10' can be achieved. Among them, the '00' and '11' states are polarization-insensitive, while the '01' and '10' states are polarization-sensitive.

For further details, the reviewer may refer to our previous study on passive transparent metasurfaces[7,8], where we already thoroughly investigated polarization-insensitive designs for enhancing transmission passively.

Our Action:

We have added brief discussions on the design concept of the metasurface in the second paragraph of **Supplementary Note 1.1**.

"To begin with, we assume that the top and bottom layers of the meta-atom are loaded with orthogonal active components to realize independent control of dual polarization, since they hold greater practical value in urban environments, where the spatial waves are arbitrarily polarized due to the multipath fading. The corresponding general dual-layered TL model is established, as depicted in **Fig. S1**a."

We have added **Supplementary Note 11** on the resonant properties of the metasurface. Fig. S14 is plotted as Fig. R5.

"Due to the frequency-dispersive nature of the metasurface, each functional mode of our design is supported by a specific resonant mode. For the SFRS mode, the simulated surface current distribution is shown in **Figs. S14**a,b. It is found that the current density exhibits significant non-uniformity across the meander-line resonators, with pronounced attenuation observed near the RF switches, which indicates the strong resonant property associated with the SRFS mode. The strong resonance may increase the parasitic loss in the prototype, lowering the Q factor of the LC resonator and thus reducing the measured peak resonance value. The simulated surface current distribution of the meta-atom operating under the RFTE mode is displayed in **Figs. S14**c,d. The results illustrate a smooth and continuous current distribution without abrupt transitions, revealing the characteristics of the weak resonance of the RFTE mode. This property mitigates the adverse effects of loss factors on the prototype, ensuring its excellent performance in the RFTE mode during measurements."

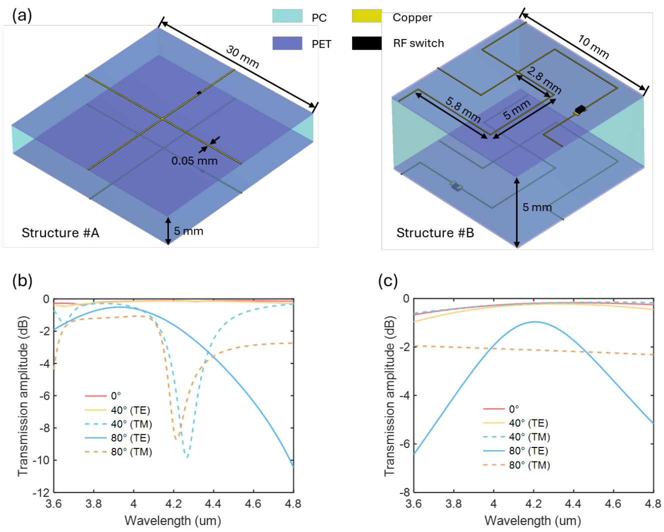

Fig. R4 (a) The geometric parameters of two candidate structures, structures #A and #B, to realize RFTE performance. (b) The simulated transmission amplitudes of structure #A. (c) The simulated transmission amplitudes of structure #B.

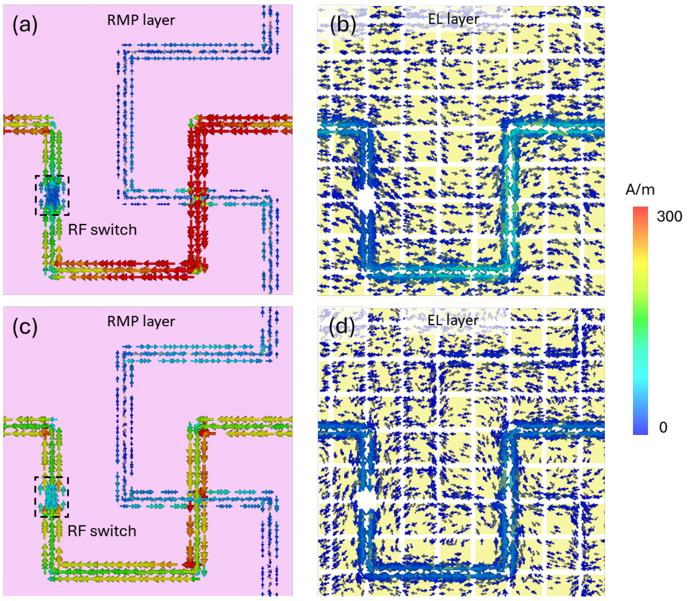

Fig. R5. (a,b) The simulated surface current distribution of the RMP and EL layer in the SRFS mode, respectively. (c,d) The simulated surface current distribution of the RMP and EL layer in the RFTE mode, respectively.

Comment 3:

The labels in Fig. 5c are unclear and difficult to read. All figure labels and annotations should be carefully revised to ensure clarity and readability.

Our Response:

Thank you very much for your good suggestion. The labels and annotations of the original Fig. 5c have been carefully modified. In addition, we have also carefully reviewed the entire manuscript to ensure that no similar mistakes remain.

Our Action:

We have replaced the original Fig. 5c with Fig. R6 in the revised manuscript.

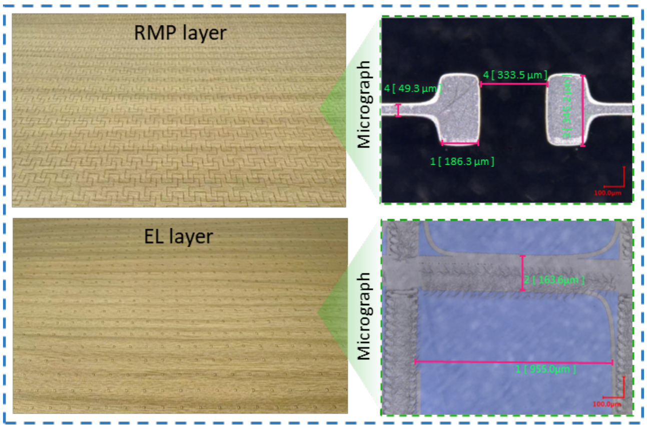

Fig. R6. The photographs of fabricated RMP and EL layers (left), and their microscope images (right).

Comment 4:

The manuscript does not mention the switching speed of the developed metasurface, which is a critical parameter for evaluating its potential in practical applications. The authors should provide at least an estimate or discussion of the switching time, supported by experimental data if available.

Our Response:

Thank you for your valuable comment regarding the switching speed of the metasurface. As suggested by the reviewer, we have added the discussion on the switching time of the prototype. The prototype is controlled by a remote-control system based on the RFID protocol with a switching speed of about 28.8 ms, which means that the functional modes of the prototype can be switched at the order of tens of times per second. Compared with the reported electrochromic smart windows that switch at the second level, the switching speed of our prototype is sufficiently fast for most application scenarios.

According to the EPC™ Radio-Frequency Identity Protocols Generation-2 UHF RFID specification (see Fig. 6.18 and Table 6.16 in the document “Gen2_Protocol_Standard”), a complete read–write operation requires the reader to send a sequence of commands, including Select, Query, Ack, QueryRep, Read, and Write. Meanwhile, the tag responds with RN16, EPC, Read data, and Write response.

We experimentally measured the read–write processes of a single and multiple tags access. With a sampling rate of about 14 Msps, the whole read-write operation of a single tag access consumed about 28.8 ms (see Fig. R7a). To manage multiple tag access and resolve signal collisions, the reader often issues a series of QueryRep commands before acquiring a correct RN16 from a tag. Thus, the complete procedure lasts about 100 ms, which is illustrated in Fig. R7b. Consequently, simultaneous control of

multiple green-smart windows leads to a slower switching speed, which nevertheless persists at the millisecond level.

Taken together, these results indicate that the metasurface can switch between the RFTE and SRFS modes on the order of tens of times per second, which is sufficient for many practical applications.

Our Action:

We have added brief discussions on the switching speed of the metasurface in the third paragraph of **"Remote control and self-powering strategy" Section**.

"According to the RFID protocol, the functional modes of the green-smart window can be switched on the order of ten times per second, which is sufficient for many practical applications."

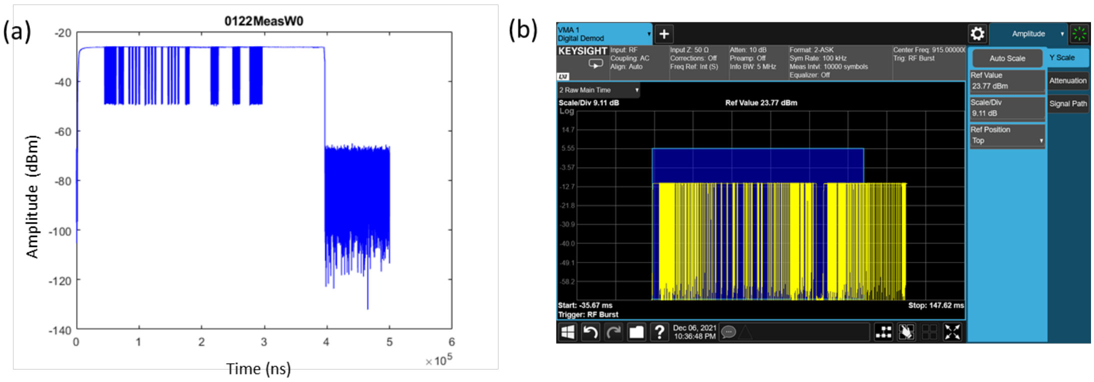

Fig. R7. The measured demodulated signal strength in the time domain in the cases of (a) a single tag access and b) multiple tags access.

Comment 5:

For clarity, the authors should provide an enlarged image of the fabricated metasurface so that the structural details of the unit cells can be clearly seen.

Our Response:

Thank you for your good comment. We agree that an enlarged image of the fabricated metasurface is necessary.

Our Action:

The viewport of the photograph has been carefully expanded to provide a broader context while retaining all key elements. Additionally, we have incorporated a zoomed-in image of the prototype (Fig. R8) into Fig. 5b of the revised manuscript to enhance clarity.

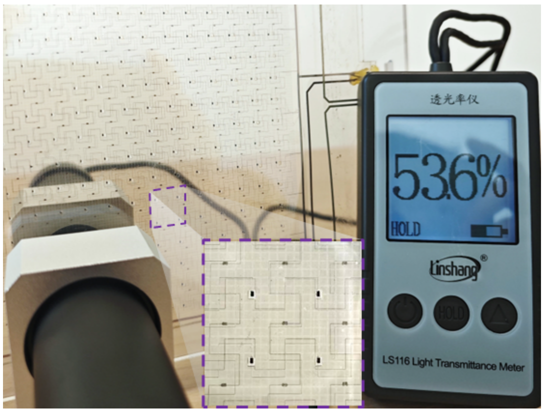

Fig. R8. The experimental setup to measure the averaged visible transmittance and the enlarged view of the prototype.

To Reviewer #3:

Comment:

This study presents a green-smart metasurface window enabling RF-IR-visible multi-band control. Its core functionalities include high visible-light transparency, low thermal emissivity, and reconfigurable RF responses tailored for wireless communications. Specifically, the RF responses can be switched between three distinct modes via embedded RF switches: wide-angle-enhanced transmission, frequency-selective shielded transmission, and polarization filtering. Notably, the reconfiguration of these modes is remotely achieved using RFID technology, with power supplied by an integrated solar panel for self-sustainability.

Green smart windows are undoubtedly key components in the future of information-driven living, and a multi-band integrated design is therefore highly imperative. While the wireless communication experiments, thermal insulation tests, and other characterizations in this work do confirm that the proposed window outperforms pure PC counterparts and some reference samples, I think the RF shielding effect is still not good enough. The reasons are stated below with other issues.

Our Response:

We sincerely appreciate your thorough review and constructive review, which have been invaluable in enhancing the quality of our manuscript. In response to your comments, we have diligently revised the manuscript, with particular emphasis on expanding the analysis of the RF shielding performance beyond the

band of interest. We believe these revisions address your concerns comprehensively and hope our responses are satisfactory.

Comment 1:

In the RFTE mode, the transmission is enhanced as compare to pure PC. The performance at large incident angle is still very good. The main issue is in the SRFS mode. Besides of shielding a particular frequency (around 4.3 GHz), it also introduces large loss to other frequencies, at least more than 5 dB from Fig. 2e and Fig.3. It is more like shielding over the whole band, not a narrow band. The shielding effect deteriorates with the incident angle.

Our Response:

Thank you very much for your professional and valuable comment. We would like to point out that the proposed prototype provides effective RF shielding in a narrow band, not over the entire frequency band. As suggested by the reviewer, RF responses across a sufficiently wide frequency band (1-8 GHz) have been simulated and measured to verify the shielding effects of the SRFS mode.

The simulated transmission amplitude spectra of the designed metasurface over 1-8 GHz are shown in Fig. R9. For the SRFS mode, the -10 dB transmission bandwidth is 3.8–4.7 GHz, with the relative bandwidth of 21%. In the frequency range of 1.1–3.1 GHz and 5-8 GHz, the transmission amplitude is higher than -5 dB, ensuring good transmission performance out of the band. In addition, a measurement setup is constructed to test the $|S_{21}|$ curves of the prototype, where a pair of Vivaldi antennas is used to transmit/receive the continuous waves, as illustrated in Fig. R10. The measured and simulated results are in good agreement, verifying that the prototype only shields the EM waves in a narrow band rather than the whole band. Table R2 compares the -10 dB transmission bandwidth of the proposed metasurface with the previously reported stopband FSSs. It is verified that the bandwidth of the designed structure is above the average level.

The selective RF shielding performance can be further improved if we relax the constraints of low IR emissivity. The -10 dB transmission bandwidth is largely determined by the Q factor; specifically, a higher loss factor results in a lower Q factor, leading to an undesirable wide bandwidth. Apart from the parasitic resistance of the RF switches, the main loss factor is the EL layer, on which the sheet resistance of the ITO is 12 Ω/sq. To interpret this point, we simulate the transmission amplitude spectra of the

meta-atom without the EL layer (only the PC plate with RMP layers). The results show that the -10 dB transmission bandwidth is 4.41-5.25 GHz with the relative bandwidth of 17%, as displayed in Fig. R11.

As noted by the reviewer, the angular stability of the SRFS mode is also important. Under the SRFS mode at an incident angle of 80°, the simulated and measured transmission amplitudes were 39.2 dB and 28.9 dB under TE polarization, while they were -14.9 dB and -9.1 dB under TM polarization, respectively. Therefore, the proposed prototype still performs well in wide-angle under dual polarizations. The angular stability of the SRFS mode is distinct in the TE and TM polarizations, which is a common problem suffered by most previously reported bandstop FSSs[14,16,18-20]. This phenomenon is primarily due to the wave impedance variation as the incident angle increases. Under TE and TM polarizations, the equivalent wave impedances for oblique incidence are given by $\eta/\cos\theta$ and $\eta\cos\theta$, respectively, where $\eta \approx 377\ \Omega$ denotes the wave impedance in free space. Since the metasurface exhibits near-zero impedance in the SRFS mode, the impedance mismatch between free space and the metasurface is aggravated under TE polarization, leading to a better shielding effect. In contrast, this mismatch is mitigated under TM polarization, resulting in reduced shielding effects under TM polarization. Given that the simulated and measured results at an 80° TM-polarized incident waves are -14.9 and -9.1 dB, respectively, the shielding performance is still applicable in numerous applications.

Our Action:

We added discussions on the broadband characteristics of the metasurface in the new **Supplementary Note 13,** titled **"Broadband characteristics across 1-8 GHz"**. Fig. S16 is plotted as Fig. R9.

"The simulated transmission amplitude spectra of the designed green-smart window over the 1-8 GHz range are shown in **Fig. S16**. For the SRFS mode, the -10 dB transmission bandwidth is 3.8-4.7 GHz, with the relative bandwidth of 21%. In the frequency ranges of 1.1-3.1 GHz and 5-8 GHz, the transmission amplitudes are higher than -5 dB, ensuring good transmission performance out of the band. During the measurement, a pair of Vivaldi antennas is used to transmit and receive the continuous waves to measure the $|S_{21}|$ curves of the prototype. The measured and simulated results are in good agreement, verifying that the prototype only shields the EM waves in a narrow band rather than the whole band……"

Table R2. Comparison of bandwidth between this work and other bandstop FSSs

Reference	Central operating frequency (GHz)	-10 dB transmission bandwidth and relative bandwidth (GHz, %)

[13]		2.4 and 5.4	1.8-2.8 (42%),<br>4.6-6.1 (28%)
[14]		0.95 and 1.84	0.6-1.2 (63%),<br>1.6-2.1 (27%)
[15]		2.45 and 5.5	2.21-2.84 (25%),<br>4.81-6.24 (26%)
[19]		26	25-30 (19%)
[16]		2	1.3-2.7 (70%)
[18]		3.1	2.8-3.4 (19%)
This work	PC + RMP + EL	4.2	3.8-4.7 (21%)
	PC + RMP	4.9	4.41-5.25 (17%)

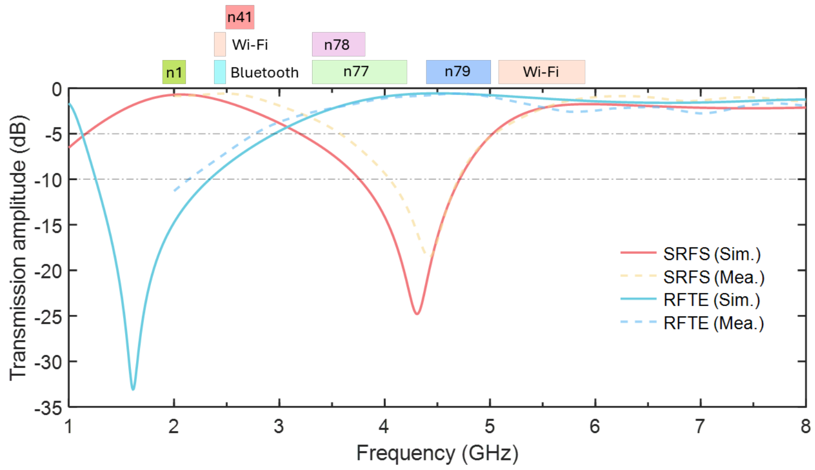

Fig. R9. The simulated and measured transmission amplitude spectra of the designed metasurface over a 1-8 GHz frequency band.

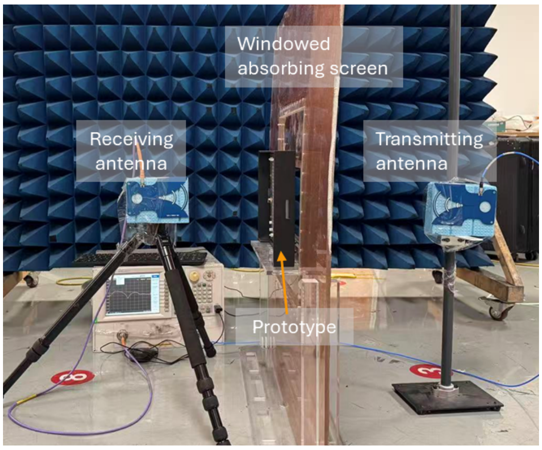

Fig. R10. The measurement setup to measure the transmission amplitude spectra over a wide frequency band.

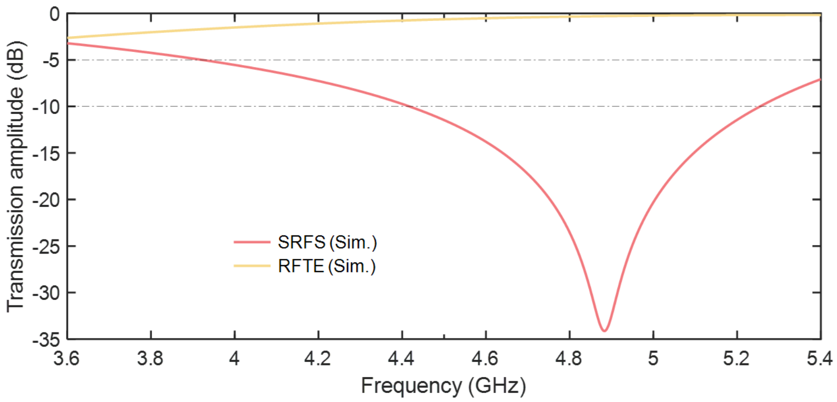

Fig. R11. The simulated transmission amplitude spectra of the metasurface without the EL layer (only the PC plate with RMP layers).

Comment 2:

In the wireless communication experiment, the I2I communication can effectively isolate the co-channel interference from O2I. But does it help to maintain strong O2I links outside the frequency band at the same time? Especially when the window introduces more insertion loss in the whole band in the SRFS mode, there is no test to verify this conclusion.

Our Response:

Thank you very much for your good comment and question. We appreciate the reviewer's concern about the O2I performance beyond the stopband of the SRFS mode. Firstly, we intend to define a signal amplitude threshold to qualify passable O2I links. According to the 3GPP (Third-Generation Partnership Project, 3GPP) TR38.901 and other research on the RF insertion loss of the buildings, the RF insertion losses of the building/transportation components have a great impact on the O2I communications[21-24]. The typical RF insertion losses of different building/transportation components are listed in Table R3. It is noted that an RF insertion loss lower than -5 dB has a negligible effect on the O2I communications. Therefore, here we rationally define a threshold of -5 dB to qualify the reliable O2I links.

Fig. R9 demonstrates the simulated transmission amplitude of the designed meta-atom under normal incidence within the frequency range of 1-8 GHz. It is found that the SRFS mode can shield the 3.8-4.7 GHz incident waves with higher than 10 dB shielding efficiency, and allow the out-of-band waves (1.1-3.1 GHz and 5-8 GHz) to transmit through with the insertion loss lower than 5 dB. The measured results

are consistent with the simulated ones, proving the window maintains strong O2I links outside the frequency band.

The meta-atom actually presents dual-band RF responses. In the upper frequency band (3.6-4.8 GHz), the ‘00’ and ‘11’ coding states function as a narrow stopband and wide passband spatial filter, respectively, as completely demonstrated in Fig. 3 in the manuscript. Conversely, in the lower frequency band (1.2-2.4 GHz), the ‘00’ and ‘11’ coding states function as a wide passband and a narrow stopband spatial filter, respectively, as shown in Fig. R9. The angular performance in the lower frequency band is inspected using full-wave simulation. As illustrated in Fig. R12, the results indicate that the wide-angle high RF transmission (‘00’ coding state) is achieved near 2 GHz, and the selective RF shielding mode (‘11’ coding state) operates around 1.6 GHz with good angular stability. The dual-band capability of the designed metasurface allows flexible manipulation of both dual-band and broadband RF signals, demonstrating good adaptability to various communication bands.

Benefiting from the dual-band property, the upper envelopes of the transmission curves (see Fig. R9) are higher than -5 dB across the entire 1.1-8 GHz frequency band, where it involves 5G primary communication frequency bands of n1(1.92-2.17 GHz), n41 (2.515-2.675 GHz), n77(3.3-4.2 GHz), n78(3.3-3.8 GHz), n79(4.4-5 GHz), and commercial wireless bands of Wi-Fi (2.4-2.48 GHz and 5.15-5.85 GHz) and Bluetooth (2.4-2.48 GHz). This indicates that, through mode switching, the metasurface can maintain good O2I links in an ultra-wide band to support diverse communication scenarios. The previously proposed passive-metasurface-enabled glass can only enhance the RF transmission within a limited operating band, while severely attenuating the transmission out of the band[7,8,25,26]. This work overcomes the inherent bandwidth limitation of conventional designs by using a reconfigurable metasurface.

Our Action:

We have added discussions of the broadband characteristics to the second paragraph in the Section **“RF Performance”** in the revised manuscript.

“……Additionally, the broadband performance of the prototype across the frequency range of 1-8 GHz is evaluated. The results indicate that, through mode switching, the designed window can maintain the transmission amplitude higher than -5 dB over 1.1-8 GHz to support diverse communication bands, and

enable selective RF shielding in two narrow bands without affecting the quality of the out-of-band O2I links (see Supplementary Note 13 for details)."

We have added discussions on the out-of-band O2I links performance of the metasurface in the new **Supplementary Note 13,** titled **"Broadband characteristics across the 1-8 GHz range"**. Fig. S16 and Fig. S17 are plotted as Fig. R9 and Fig. R12, respectively.

"……It is worth mentioning that the upper envelopes in **Fig. S16** are higher than -5 dB across the entire 1.1-8 GHz frequency band, where it involves 5G primary communication frequency bands of n1(1.92-2.17 GHz), n41 (2.515-2.675 GHz), n77(3.3-4.2 GHz), n78(3.3-3.8 GHz), and n79(4.4-5 GHz), and commercial wireless bands of Wi-Fi (2.4-2.48 GHz and 5.15-5.85 GHz) and Bluetooth (2.4-2.48 GHz). This indicates that the metasurface can maintain good O2I links in an ultra-wide band to support diverse communication scenarios through mode switching. The previously proposed passive-metasurface-enabled glass can only enhance the RF transmission in a limited operating band, while severely attenuating the transmission out of band[8,9,13,15]. This work overcomes the inherent bandwidth limitation of conventional designs by using the reconfigurable metasurface.

Additionally, the meta-atom actually presents dual-band RF responses. In the upper frequency band (3.6-4.8 GHz), the '00' and '11' coding states function as narrow stopband and wide passband spatial filters, respectively, as completely demonstrated in Fig. 3 in the main text. Conversely, in the lower frequency band (1.2-2.4 GHz), the '00' and '11' coding states function as wide passband and narrow stopband spatial filters, respectively, as shown in **Fig. S16**. The angular performance in the lower frequency band is thoroughly inspected using the full-wave simulation. As illustrated in **Fig. S17**, the results indicate that the wide-angle high RF transmission ('00' coding state) is achieved near 2 GHz, and the selective RF shielding mode ('11' coding state) operates around 1.6 GHz with good angular stability. The dual-band capability of the designed metasurface allows flexible manipulation of both dual-band and broadband RF signals, demonstrating good adaptability to various communication bands."

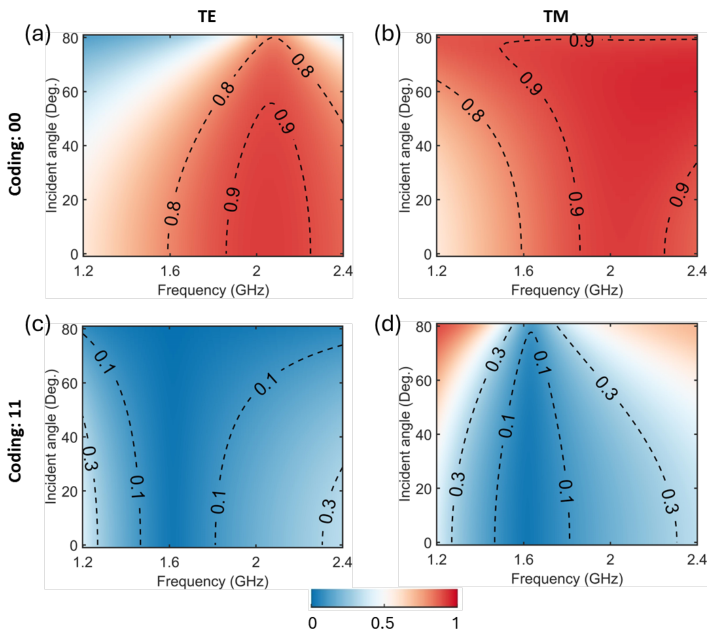

Fig. R12. The simulated transmission spectra of the green-smart window in the angular range of 0°-80°. (a,b) The results in ‘00’ coding state under TE and TM polarizations, respectively. (c,d) The results in ‘11’ coding state under TE and TM polarizations, respectively.

Table R3. RF insertion loss of typical building/transportation components

Typical building/transportation components	Frequency (GHz)	Insertion loss (dB)
Low-loss materials: Gypsum, bare glass, wooden door, building’s interior wall	0.8-28	5-10 dB
Medium-loss materials: building’s exterior wall, concrete	0.8-28	8-16 dB
High-loss materials: high-speed train window, reinforced concrete	0.8-28	23-33 dB

Comment 3:

The window integrates the RFID and solar panel for self-powering. This part is more like an engineering issue. However, it is not clearly explained whether this is a direct superposition of existing technologies or involves technological innovation.

Our Response:

Thank you very much for your insightful comment. We would like to point out that our work focuses on realizing the green-smart window at the system level. The green-smart window aperture, composed of

a multispectral metasurface, is the key innovation in our work. The highly integrated RFID tag and the solar panel, as a functional module, are designed to solve the thorny engineering issues of control method and power supply.

To ensure system-level realization of the green-smart window, we develop a novel RFID tag design with three key advantages: 1) A single-layered metal-substrate-metal structure ($0.08\times0.37\lambda^2$) minimizing cost, weight, and visual obstruction; 2) Full integration of IoT components (excluding the mobile RFID reader) into a miniature device; 3) Strategic integration of silicon solar cells to the RFID tag in a "face-to-face" configuration, achieving power supply, energy storage, and accessibility of IoT, while mitigating visibility loss from opaque components. To the best of our knowledge, the integration between the RFID tag and the solar panel has not been reported before.

To be more specific, the advantages of the novel RFID tag contain the incorporation of advanced technologies, highly integrated implementations, and system robustness, as illustrated below.

1) Selection of advanced technologies

To eliminate the external power dependency, a direct approach is to utilize the low-cost commercial silicon-based solar cells[27,28]. However, using conventional photovoltaic systems (e.g., rooftop solar arrays) results in extensive cabling, increasing the deployment complexity. While large-scale silicon solar cells integrated with windows can provide energy supplies, their opacity severely compromises visible transparency. Recent advances in perovskite solar cells and luminescent solar concentrators offer alternatives, yet these technologies remain exploratory due to high costs, low energy efficiency, and inconsistent performance at scale[29-32]. Therefore, for the low cost, easy implementation, high energy efficiency, and negligible light blocking, we prioritize integrating a miniature solar cell panel into the smart window with low power consumption.

To overcome the limitations of the cumbersome control cables, various remote control technologies have been explored, including those based on voice, touch, infrared, ultraviolet, RF waves, and so on. Among them, the remote control via RF waves is particularly suitable for smart devices, since the emerging IoT technologies can regulate the massive users' terminals orderly and adaptively. Among the prevailing IoT technologies, the RFID protocol is highly compatible with the smart windows due to its long-range control and extremely low power consumption.

2) Highly integrated implementations

Although IoT-enabled smart windows have emerged in proof-of-concept studies, their integration with IoT systems remains suboptimal. Specifically, effective integration requires compact unification of these components: 1) a window device for on-demand optical control, 2) a microcontroller unit for environmental data processing and I/O management, 3) an operating circuit for currents/voltage regulation. 4) a wireless communication module linking the MCU and mobile devices. 5) a mobile application for remote control. 6) A power supply for the MCU and I/O ports.

To address these limitations, we integrate all the necessary IoT components (excluding the mobile RFID reader) into a miniature RFID tag. This development paves the way for fully cable-free smart windows, significantly boosting their commercial practicality and user appeal.

3) System robustness

For most reported work, the sensors are just placed near a large-scale metasurface array without considering the mutual effects between them, leading to a worrying system robustness. In this work, considering that the prototype may result in distorted radiation patterns of the RFID tag, we design and apply a small-area transparent absorber as an additional component to prevent the mutual coupling and improve the system stability. As shown in Fig. R13 (see Fig. S11 in the Supplementary Note 8), the simulated results show that the RFID tag performs outstandingly regardless of the size and functional modes of the window, thereby further improving the robustness of the proposed control and powering strategy.

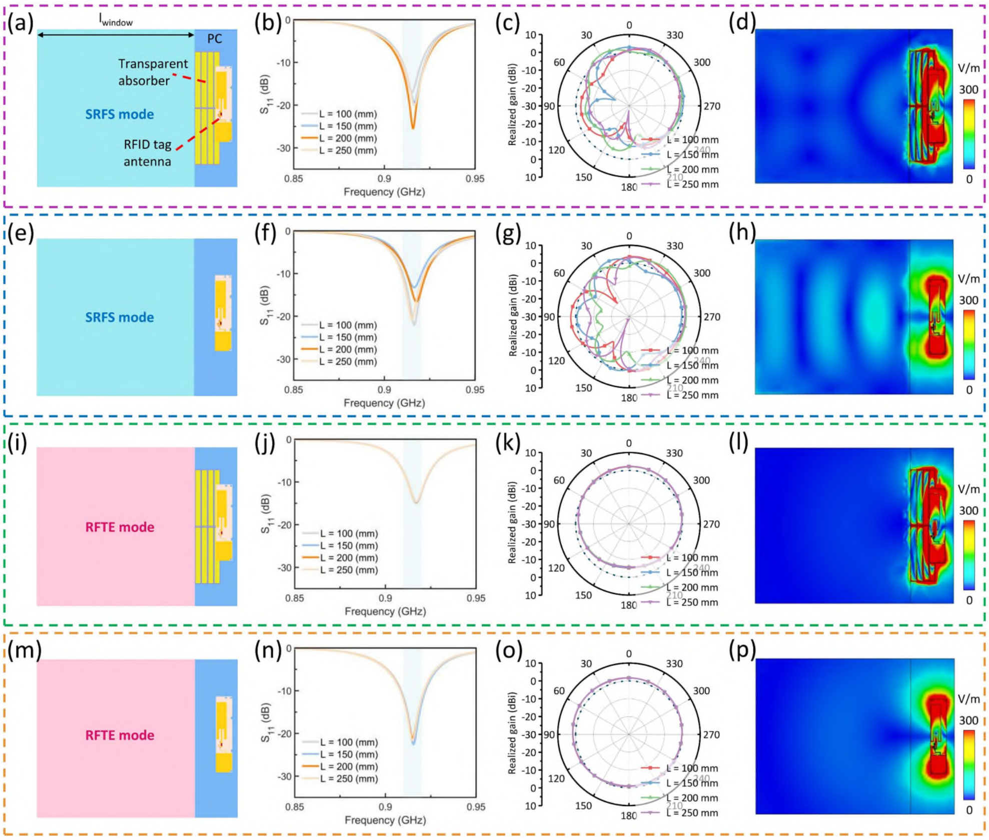

Fig. R13. (a,e,i,m) Four simulation scenarios in the full-wave simulation; (b,f,j,n) S11 parameters regarding lwindow; (c,j,k,o) radiation patterns regarding lwindow; and (d,h,l,p) E-field intensity distribution. The results under the cases of (a-d) with the transparent absorber in the SRFS mode, (e-h) without the transparent absorber in the SRFS mode, (i-l) with the transparent absorber in the RFTE mode, and (m-p) without the transparent absorber in the RFTE mode.

Our Action:

We have added discussions on the novelty of the designed RFID tag in the last paragraph in Supplementary Note 6 **"System Configurations for Remote Control and Self-Powering"**.

"The innovations of the controlling and powering system can be briefly summarized as: 1) a single-layered metal-substrate-metal structure (0.08×0.37λ^2) that minimizes the cost, weight, and visual obstruction; 2) full and compact integration of the IoT components, including the multispectral metasurface, microcontroller unit, operating circuit, wireless communication module, mobile application, and power supply; and 3) strategic integration of silicon solar cells to the RFID tag in a "face-to-face" configuration, achieving power supply, energy storage, and accessibility of IoT, while mitigating the visibility loss from opaque components."

Comment 4:

Does the design compromise performance in the visible and IR bands to facilitate RF modulation? The visible transparency is 53.6% (average) and 62% (maximum). How is it compared to the PC? The IR emissivity is 0.35, which is not low enough. From the cavity temperature experiment, it is only a little better than pure PC, and still far from the thermal isolation film.

Our Response:

Thank you very much for your valuable comments. As correctly noted by the reviewer, the visible and IR performances are actually slightly compromised due to the RF modulation. We would like to emphasize that the proposed green-smart window is designed for the dynamic manipulation of multispectral EM waves, featuring long-range wireless control and high energy efficiency. Therefore, the prototype is not a fixed single-function or single-spectral material, but rather a versatile green-smart system. The measured transparency of the prototype is 53.6% on average and reaches a maximum of 62%, fully complying with industry standards for architectural glass (see the Industry Standard Documents of Technical Specification for Application of Architectural Glass, JGJ 113-2015). Furthermore, outdoor field measurements demonstrate that the prototype, with an IR emissivity of 0.35, achieves a significant thermal isolation effect compared to a bare PC plate. We acknowledge that the visible transmittance and IR emissivity are not at the highest levels compared with the single-function/single-spectral materials. Specifically, for the bare PC plate, the average visible transmittance is measured as 90.2%, as shown in Fig. R14a. But its IR emissivity is usually higher than 0.8, leading to limited thermal isolation capacity. While the commercial thermal isolation (CTI) film provides excellent thermal insulation, it reduces visible light transmittance to only 16.2% (see Fig. R14b) and nearly completely blocks RF transmission throughout the entire radio frequency band.

To show the superiority of our current design, we compare the performance of the prototype with previously reported multispectral materials, as summarized in Table R4. The proposed design demonstrates superior RF performance while maintaining competent properties in the visible and IR regimes. In addition, when evaluated against any single performance metric, all reported multispectral materials exhibit certain performance compromises relative to their fixed single-function or single-spectral counterparts.

Due to the distinct modulation mechanisms of the visible, IR, and RF bands, the multispectral performances usually have mutual restriction, as interpreted in detail below:

1) The relationship between RF and IR performance

For qualified IR performance, the window surface should be covered by low-E materials, such as the EL layer in our design. For the EL layer, a lower ohmic sheet or a higher filling ratio leads to a lower surface impedance $|Z_{ITO}|$, which severely impairs the RF performance, as shown in Figs. S2b,c in the Supplementary Note 1.3. To further interpret this point, we redesign a meta-atom without the EL layer (only PC plate with RMP layers), as displayed in Fig. R15. The simulated RF performances of the original meta-atom and the redesigned one are compared in Fig. R16. It is observed that both the RFTE and SRFS performances can be further improved without considering the IR performance.

2) The relationship between IR and visible performance

The inverse correlation between the IR emissivity and the visible transmittance is a classic contradiction problem in the material field[25,33,34]. The reason can be concluded as high free electron density (essential for low IR emissivity, ε) inevitably enhances visible light absorption, at the expense of reduced visible transmittance, while conversely, low free electron density (required for high visible transmittance) fails to effectively reflect IR radiation.

3) The relationship between visible and RF performance

We emphasize that the visible and RF performance in our design is not in a contradictory relationship, breaking the limitations of the most previously reported transparent metasurface. If the IR performance is not considered, the removal of the EL layer can improve the performance in both the RF and visible spectra, as compared in Figs. R9 and R11. In addition, for the RMP layer, the RF performance regarding the linewidth w of the meander-line resonators is evaluated using full-wave simulation. As illustrated in Fig. R17, the maximum transmission amplitudes in the RFTE mode are lifted when the linewidth w gets thinner, and the peak resonance values of the SRFS mode are hardly affected by linewidth w. Since the visible transparency will obviously improve when the linewidth w gets thinner, the RF and visible performance can be jointly enhanced by using ultra-fine metallic lines.

Our Action:

We have added brief discussions on the visible transparency of the bare PC plate in the Section **"Visible performance"**. Fig. S12 in Supplementary Note 9 is plotted as Fig. R14.

"In our design, the measured visible transmittance of the 5-mm-thick bare PC plate is up to 90.2% (see Supplementary Note 9 for details). To minimize the visual blockage, ultrafine meander lines are employed to form the metasurface patterns."

We have added brief discussions on the visible transparency of the CTI film in the Section **"Thermal regulation"**.

"However, the average visible transmittance of the CTI film is only 16.2% (see Supplementary Note 9 for details)."

Table R4. Comparison of state-of-the-art multispectral materials in the literature

Reference	RF performance			IR emissivity	Visible transmittance
	Reconfigur-ability	Functions	Angular and polarization sensitivity		
[35]	No	Polarization conversion and absorption	0°-60°, single polarizations	0.3	opaque
[36]	No	Tuning reflection amplitude and phase	0°-60°, dual polarizations	0.3	50%
[37]	No	Polarization conversion	0°, single polarizations	0.6~0.8	opaque
[38]	No	EM shielding in the entire RF band (like metal ground)	N.A., dual polarizations	N.A.	0.1%~81.7% (dynamically tunable)
[39]	No	RF absorption	0°-60°, dual polarizations	0.35~0.63	60% (average)
[40]	No	N.A.	N.A.	0.247	50%
This work	Yes	Switching between SRFS and RFTE	0°-80°, dual polarizations	$\varepsilon_{3\text{-}5um} = 0.35$<br>$\varepsilon_{8\text{-}14um} = 0.33$	53.6% (average)<br>62% (maximum)

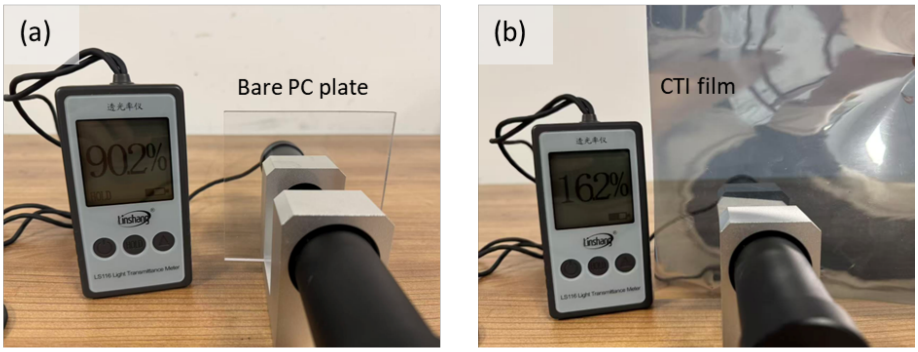

Fig. R14. The experimental setup to measure the average visible transmittance of (a) the bare PC plate and (b) the CTI film, which are measured as 90.2% and 16.2%, respectively.

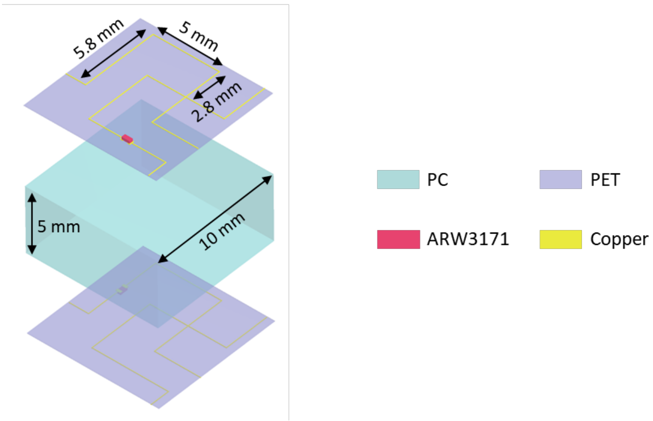

Fig. R15. The geometric parameters of the redesigned meta-atom without EL layers.

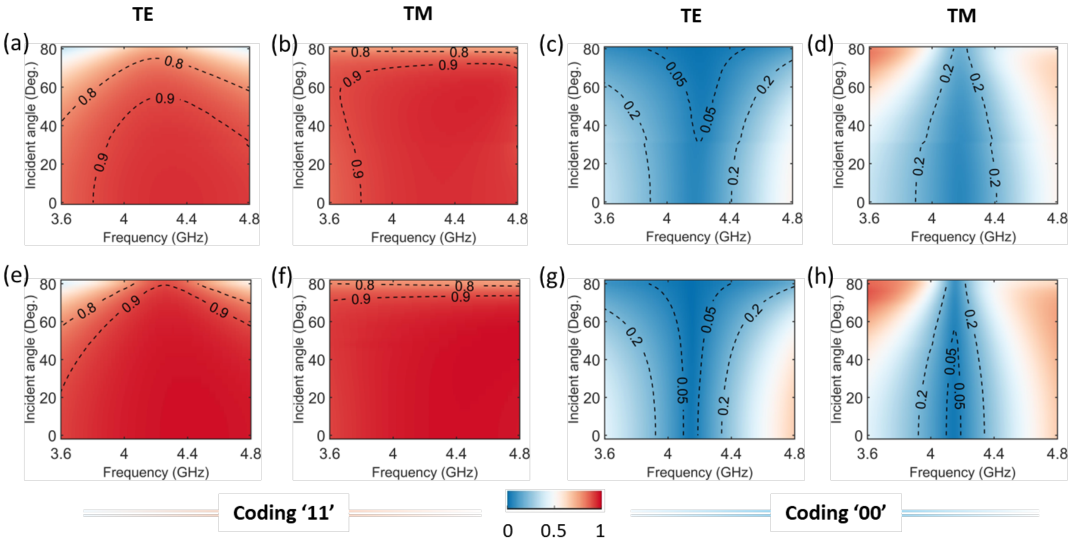

Fig. R16. (a-d) The simulated transmission spectra of the proposed meta-atom in SRFS and RFTE modes under dual polarizations. (e-h) The simulated transmission spectra of the redesigned meta-atom without EL layers in SRFS and RFTE modes under dual polarizations.

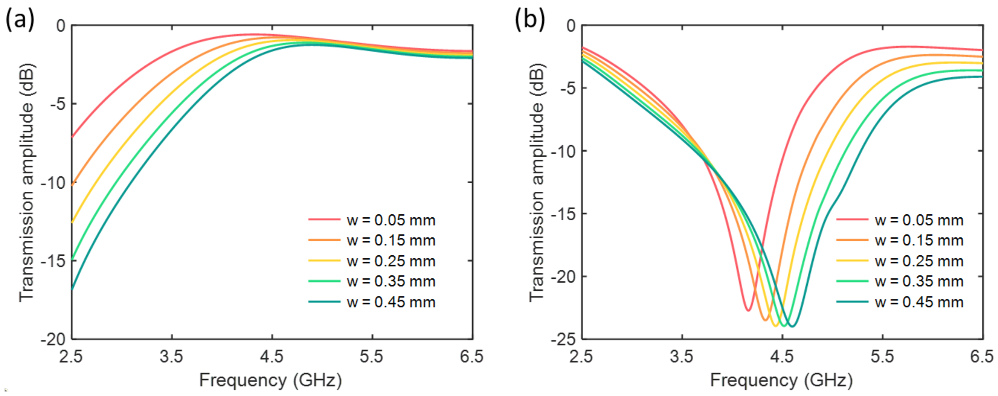

Fig. R17. The simulated transmission amplitudes of the meander-line resonators against different linewidths *w* in the modes of (a) RFTE and (b) SRFS, respectively.

Comment5:

From the supplementary details, the EL layer and RMP layer are separately designed from the circuit model. Is there any coupling between them?

Our Response:

Thank you very much for your insightful question. As suggested by the reviewer, we test the coupling between the EL and RMP layers. The surface current distributions of the EL and RMP layers of the proposed meta-atom are simulated, and the results are shown in Fig. R5. For clarity, Fig. R5 is copied as Fig. R18 to show in this response. It is found that the RMP has a slight impact on the surface current distribution of the EL layer, and vice versa. There arises a coupling between the EL and RMP layers due to their conductive structures and a small interlayer spacing of 0.125 mm. As a result, the transmission amplitude spectra of the meta-atoms with and without the EL layer are distinct, as shown in Fig. R9 and Fig. R11.

In the supplementary Note 1, we demonstrate the feasibility of switching between the RFTE and SRFS modes, high visible transparency, and low IR emissivity, respectively. The analysis indicates that the superior RF performance requires the composite film coatings (RMP and EL layers) that provide high shunt inductance of X_c = 953 Ω, as shown in Fig. S5 in the Supplementary Note 3. In contrast, desired IR performance is attained through metal/metal-oxide patches with a high filling ratio exhibiting capacitance characteristics (X_e = 18 - 2578i Ω), as illustrated in Fig. S2d in the Supplementary Note 1.3. This implies that the RMP and EL layers should be integrated elaborately. Specifically, the equivalent inductance of the RMP layer should be sufficiently high to overwhelm the capacitance of the EL layer.

Despite the inevitable coupling between these structures, the designed meta-atom achieves superior multispectral performance through parameter optimization, attaining the optimal trade-off.

Our Action:

We have added the discussion on the surface current distribution of the designed meta-atom in the **Supplementary Note 11** titled **"Resonant Properties of the RFTE and SFRS Modes"**. Fig. S14 in the Supplementary Note 11 is plotted as Fig. R18.

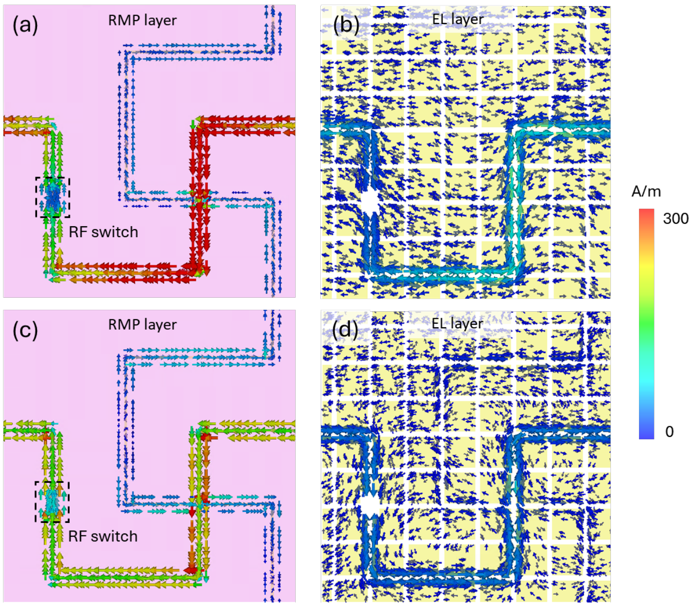

Fig. R18. (a,b) The simulated surface current distribution of the RMP and EL layer in the SRFS mode, respectively. (c,d) The simulated surface current distribution of the RMP and EL layer in the RFTE mode, respectively.

Comment 6.1:

In Fig. 2g, the area is not large enough above and below the window to observe the field distribution. From the colorbar, what is the reason that the maximum values in TE and TM modes are very different?

Our Response:

We appreciate the reviewer's careful inspection of the original Fig. 2g. The difference between maximum values in TE and TM modes is caused by the limited viewport of the E- and H-field distributions. In the revised manuscript, we have expanded the viewport to fully capture the field profiles. As shown in Fig. R19, both E- and H-field distributions are clearly visible. To standardize the field density representation across different polarization modes, only the in-plane components of the field vectors are recorded.

Specifically, the E-field vectors are captured in the TE mode, while the H-field vectors are recorded in the TM mode. The maximum values of the colorbar in the E-field and H-field distributions are set to 2 V/mm (equivalent to 2000 V/m) and 5.3 A/m, respectively, consistent with the free-space wave impedance (2000 / 5.3 ≈ 377 Ω).

Our Action:

We have replotted the original Fig. 2g as Fig. R19 in the revised manuscript.

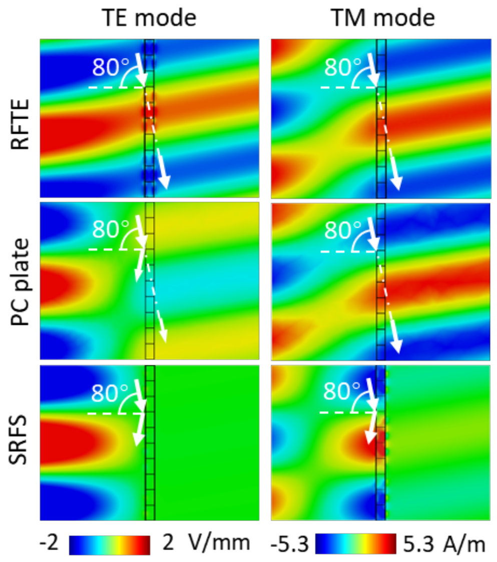

Fig. R19. The instant intensity distributions of the PC plate and meta-atom in the SRFS and RFTE modes when illuminated by 80° incidence at 4.2 GHz.

Comment 6.2:

In the reference, there are a lot of literature information is missing.

Our Response:

Thank you very much for your good comment. We apologize for the oversights in our initial literature citations and confirm that all references have been meticulously verified and updated in the revised manuscript, supplementary information, and the response letter.

Our Action:

We have ensured that all references now comply with the required formatting guidelines in the revised manuscript.

Comment 6.3:

Fig. 4(d) the unit should be changed from GHz to MHz.

Our Response:

Thank you very much for your good comment. As suggested by the reviewer, we have revised the typing error in the original Fig. 4d and reviewed the entire manuscript to ensure no similar mistakes remain.

Our Action:

We have corrected the unit in Fig. 4(d) from GHz to MHz in the revised manuscript.

Comment 6.4:

In Fig. 7b, the PC+EL already shows good improvement in communication enhancement in oblique incidence. What is the reason? And how to accurately account for the contribution of the metasurface?

Our Response:

Thank you very much for your valuable comment and questions. While the RF transmission amplitude of the PC plate with EL layers (REF #3) is moderate relative to the CTI film (REF #2), as noted by the reviewer, it is noteworthy that its performance remains reduced compared to the bare PC plate, as illustrated in Figs. S15a-h in Supplementary Note 12. To further interpret this point, we have simulated transmission amplitude spectra of the PC with EL layers and the bare PC plate, as shown in Fig. R20.

To quantitatively account for the contribution of the metasurface, a transmission line model is established to calculate the transmission amplitudes of the structure. The substrate and air can be regarded as transmission lines with the intrinsic impedance of Z_s and Z_0, respectively, and the metasurface coatings attached to the substrate can be modeled as a parallel admittance X_f, as illustrated in the inset in Fig. R21. By introducing the extracted surface reactance X_f of the proposed composite film coating (RMP and EL layers) and the EL layer, with $X_f = 953\ \Omega$ and $2578\ \Omega$, respectively, into the transmission line model, the transmission amplitudes of the proposed meta-atom and the PC plate with EL layers can be calculated, as marked by red and blue dotted lines in Fig. R21, respectively. The simulated and calculated results are in good agreement, despite the slight deviation due to the loss factors that are not considered in the transmission line model.

In summary, the designed metasurface in the RFTE mode can enhance the wide-angle RF transmission compared with both the bare PC plate and the PC with EL layers, as shown in Table R5.

Table R5. Comparison of transmission amplitudes at 4.2 GHz for different samples

Samples	Polarizations	Transmission amplitudes under different incident angles				
		0°	20°	40°	60°	**80°**
Meta-atom (Sim.)	TE	0.94	0.93	0.92	0.89	**0.72**
	TM	0.94	0.94	0.95	0.94	**0.76**
PC (Sim.)	TE	0.94	0.93	0.91	0.81	**0.44**
	TM	0.94	0.95	0.98	0.99	**0.83**
PC+EL (Sim.)	TE	0.89	0.87	0.82	0.68	**0.31**
	TM	0.89	0.90	0.95	0.99	**0.83**
PC+EL (Cal.)	TE	0.91	0.90	0.85	0.72	**0.35**
	TM	0.91	0.92	0.96	0.99	**0.83**

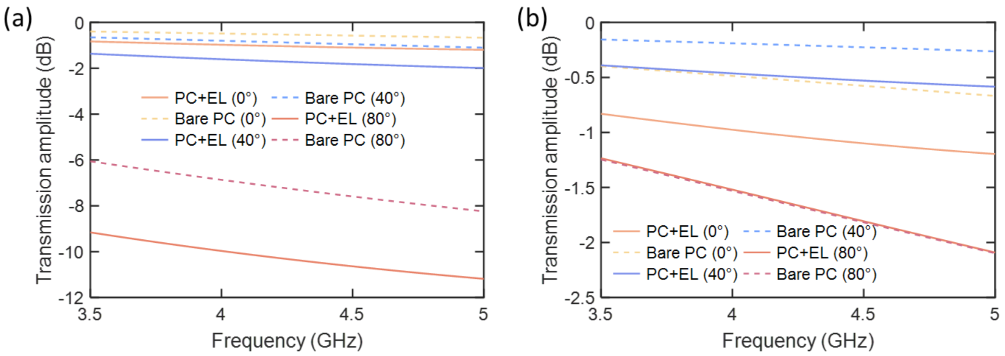

Fig. R20. The simulated transmission amplitude spectra of the PC plate with EL layers and the bare PC plate under (a) TE and (b) TM polarization, respectively.

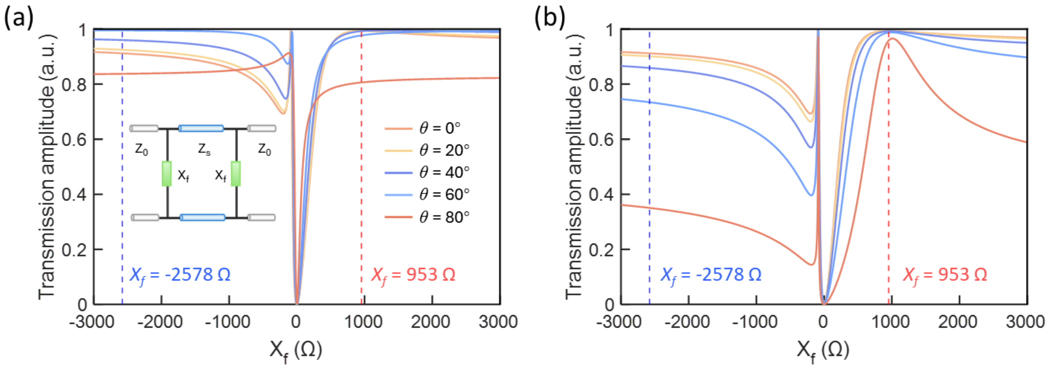

Fig. R21. The calculated transmission amplitudes based on the transmission line model under (a) TE and (b) TM polarization, respectively. The transmission line model is shown in the inset. The red and blue dotted lines correspond to the extracted surface reactance X_f of the proposed composite film coating and the EL layer.

Comment 6.5:

The REF number in the figure caption, main text and inside the figure are not consistent in Fig. 7b,e.

Our Response:

Thank you very much for your good comment. As noted by the reviewer, we have revised the typographical errors in the original Figs. 7b,e captions. In addition, we have reviewed the entire manuscript to ensure no similar errors remain.

Our Action:

We have unified the REF numbers in Figs. 7b,e in the revised manuscript.

References in the Response Letter

1 Xia, C., Lu, Z., Zhang, Y. & Tan, J. Broadband High Optical Transparent Intelligent Metasurface for Adaptive Electromagnetic Wave Manipulation. *Research* **7**, 0334 (2024).

2 Lu, Z., Xia, C., Zhang, Y. & Tan, J. Metasurface with Electrically Tunable Microwave Transmission Amplitude and Broadband High Optical Transparency. *ACS Appl. Mater. Interfaces* **15**, 29440-29448 (2023).

3 Cai, X. *et al.* Metasurface Window With Customized Transparency for Sunlight and Switchable Transmission for Microwaves. *IEEE Trans. Microw. Theory Tech*. **7**, 3670-3682 (2024).

4 Liang, J. C. et al. An Optically Transparent Reconfigurable Intelligent Surface with Low Angular Sensitivity. *Adv. Opt. Mater.* **12**, 2202081 (2024).

5 Zhang, H. *et al.* Electric field oriented deposition manufacturing of low loss, high gain flexible transparent antenna utilizing the skin effect. *Int. J. Extreme Manuf.* **73**, 055103 (2025).

6 Li, S. *et al.* Self-adaptive energy-efficient windows with enhanced synergistic regulation of broadband infrared thermal radiation. *Nano Energy* **129**, 110023 (2024).

7 Jiang, R. Z. et al. Optically Transparent Metasurface with High RF Transmittance and Wide-Angle Stability for Dual Bands and Dual Polarizations. *Adv. Opt. Mater.* **11**, 2300553 (2023).

8 Jiang, R. Z. et al. A Single-Layered Wideband and Wide-Angle Transparent Metasurface for Enhancing the EM-Wave Transmissions Through Glass. *IEEE Trans. Antennas Propag.* **71**, 6593-6605 (2023).

9 Lv, Q. *et al.* Ultrawide-Angle Ultralow-Reflection Phenomenon for Transverse Electric Mode in Anisotropic Metasurface. *Adv. Opt. Mater.* **10,** 2102400 (2022).

10 Zhou, Q. Y. *et al.* Decoupling Strategy of Metasurface to Realize Independent Sensing and Wave Manipulations at the Same Time. *Adv. Mater.* **37**, 2500266 (2025).

11 Jiang, R. Z. et al. Simultaneously Intelligent Sensing and Beamforming Based on an Adaptive

Information Metasurface. *Adv. Sci.* **11**, 2306181 (2023).

12 Liang, J. C. *et al.* An Angle-Insensitive 3-Bit Reconfigurable Intelligent Surface. *IEEE Trans. Antennas Propag.* **70**, 8798-8808 (2022).

13 Farooq, U., Iftikhar, A., Najam, A. I., Khan, S. A. & Shafique, M. F. An optically transparent dual-band frequency selective surface for polarization independent RF shielding. *Opt. Commun.* **546**, 129824 (2023).

14 Yang, Y., Li, W., Salama, K. N. & Shamim, A. Polarization Insensitive and Transparent Frequency Selective Surface for Dual Band GSM Shielding. *IEEE Trans. Antennas Propag.* **69**, 2779-2789 (2021).

15 Farooq, U., Shafique, M. F. & Mughal, M. J. Polarization Insensitive Dual Band Frequency Selective Surface for RF Shielding Through Glass Windows. *IEEE Trans. Electromagn. Compat.* **62**, 93-100 (2020).

16 Dewani, A. A., O'Keefe, S. G., Thiel, D. V. & Galehdar, A. Window RF Shielding Film Using Printed FSS. *IEEE Trans. Antennas Propag.* **66**, 790-796 (2018).

17 MUNK & Ben, A. *Frequency Selective Surfaces: Theory and Design*. (Frequency Selective Surfaces: Theory and Design, 2005).

18 Ferreira, D., Cuinas, I., Fernandes, T. R. & Caldeirinha, R. F. S. Multi-Semicircle-Based Single- and Dual-Band Frequency-Selective Surfaces: Achieving Narrower Bandwidth and Improved Oblique Incidence Angular Stability. *IEEE Antennas Propag. Mag.* **61**, 32-39 (2019).

19 Dey, S., Dey, S. & Koul, S. K. Second-Order, Single-Band and Dual-Band Bandstop Frequency Selective Surfaces at Millimeter Wave Regime. *IEEE Trans. Antennas Propag.* **70**, 7282-7287 (2022).

20 Darvish, A. & Kishk, A. A. X-Band Nearfield Shielding Metasurface With Adjustable Reflection/Transmission Zeroes. *IEEE Trans. Electromagn. Compat.* **64**, 1602-1613 (2022).

21 Zhu Q, et al. 3GPP TR 38.901 channel model. Wiley 5G Ref. (2021)

22 I.Rodriguez. et al. An Empirical Outdoor-to-Indoor Path Loss Model From Below 6 GHz to cm-Wave Frequency Bands. IEEE Antennas Wirel. Propag. Lett. 16, (2017)

23 B. Guo et al. Building entry loss model for 24 to 31GHz band. 2016 International Symposium on Antennas and Propagation (ISAP), (2016).

24 M. Inomata. et al. Outdoor-to-indoor path loss model for 8 to 37 GHz band. 2015 International Symposium on Antennas and Propagation (ISAP), (2015).

25 Safari, M., Kherani, N. P. & Eleftheriades, G. V. Multi-Functional Metasurface: Visibly and RF Transparent, NIR Control and Low Thermal Emissivity. *Adv. Opt. Mater.* **9**, 2100176 (2021).

26 Safari, M., He, Y., Kim, M., Kherani, N. P. & Eleftheriades, G. V. Optically and radio frequency (RF) transparent meta-glass. *Nanophotonics* **9**, 3889-3898 (2020).

27 Kang, Y. *et al.* Comprehensive photovoltaic system in roofs, opaque walls, and windows toward zero-energy buildings utilizing multi-objective optimization. *J. Build. Eng.* **104**, 112320 (2025).

28 Niu, Y. *et al.* Energy Saving and Energy Generation Smart Window with Active Control and Antifreezing Functions. *Adv. Sci.* **9**, 2105184 (2022).

29 Liu, Y. *et al.* Full-frame and high-contrast smart windows from halide-exchanged perovskites. *Nat. Commun.* **12**, 3360 (2021).
30 Koh, T. M. *et al.* Halide Perovskite Solar Cells for Building Integrated Photovoltaics: Transforming Building Façades into Power Generators. *Adv. Mater.* **34**, 2104661 (2022).
31 Wang, L., Wang, X. & Zhao, H. Highly Efficient Luminescent Solar Concentrators Based on Capped Carbon Quantum Dots with Unity Quantum Yield. *Adv. Funct. Mater.* **35**, 2423422 (2025).
32 Huang, S. C. *et al.* Integrated device of luminescent solar concentrators and electrochromic supercapacitors for self-powered smart window and display. *Nat. Commun.* **16**, 2085 (2025).
33 Zheng, J., Zheng, H., Pang, Y., Qu, B. & Xu, Z. A Metasurface Glass for Energy Saving and 5G Mobile Communication Signal Enhancement. *Small* **21**, 2408598 (2024).
34 Zheng, J., Zheng, H., Pang, Y., Qu, B. & Xu, Z. Metasurface Glass for Wireless Communication and Energy Saving. *Small* **20**, 2309050 (2024).
35 Wang, Y. *et al.* Detection and Anti-Detection with Microwave-Infrared Compatible Camouflage Using Asymmetric Composite Metasurface. *Adv. Sci.* **11**, 2410364 (2024).
36 Jiang, J. *et al.* Multi-spectral compatible metasurface with low infrared emissivity, independent microwave complex-amplitude control, and high visible transparency. *Opt. Lett.* **49**, 3174-3177 (2024).
37 Sun, H. T. *et al.* Noninvasive inset-integrated meta-atom for achieving single-layer metasurface simultaneously with coded microwave reflectivity and digitalized infrared emissivity. *Nanophotonics* **13**, 3113-3122 (2024).
38 Ma, D. *et al.* Solar Light Management Enabled by Dual-Responsive Smart Window. *ACS Appl Mater Interfaces* **14**, 56065-56073 (2022).
39 Cui, Y. *et al.* Visible Transparent Wideband Microwave Meta-Absorber with Designable Digital Infrared Camouflage. *Adv. Opt. Mater.* **12**, 2301712 (2023).
40 Du, X. *et al.* Visible transparent, infrared stealthy polymeric films with nanocoating of ITO@MXene enable efficient passive radiative heating and solar/electric thermal conversion. *Nano Res.* **16**, 3326-3332 (2022).
